# Supplementary material for: The transmission blocking activity of artemisinin-combination, non-artemisinin, and 8-aminoquinoline antimalarial therapies: A pooled analysis of individual participant data
Source: PLoS Med. 2025 Aug 14;22(8):e1004683. doi: 10.1371/journal.pmed.1004683 (PMC12352847; doi:10.1371/journal.pmed.1004683)
Supplement: S1 Appendix — Text A. Antimalarial treatment dosage. Dosing schedules for each treatment regimen. Table A. Antimalarial treatment suppliers. Suppliers of study drugs used in each study. Fig A. Original PQ03 molecular gametocyte densities. Bland-Altman plot presenting the agreement between gametocyte density measured by microscopy (x-axis) and RT-qPCR (y-axis) pre-treatment. The original PQ03 (2016) RT-qPCR data is presented here, which showed that the comparison of densities measured by both methods for this study was an outlier compared to the other studies. The solid black line indicates the overall mean difference including all studies, and dotted lines represent 1.96 x standard deviation of the differences. The dashed lines, coloured by study, represent group-specific mean differences. Only in 2016 (PQ03 study), there was a relevant difference between the two gametocyte measurements; molecular assays were repeated for this sample set prior to inclusion in the analyses. Table B. Baseline descriptives per treatment group. Study participants characteristics, parasite densities and infectivity prior to treatment. Fig B. Relation between oocyst density and prevalence. Association between oocyst prevalence and oocyst density, determined by a mixed-effects logistic regression with random effect for study, where the solid black line represents the association averaged over studies. Table C. Relative reduction in gametocyte prevalence. Relative reduction compared to baseline in gametocyte prevalence by microscopy and RT-qPCR, at three time points (Day 2, Day 7, Day 14), with 95% confidence intervals. Fig C. Relative reduction in gametocyte prevalence per study arm (ungrouped). Bar charts illustrating the relative reduction compared to baseline in gametocyte prevalence by microscopy and RT-qPCR for each study arm (ungrouped), over three time points (Day 2, Day 7, Day 14). Vertical bars depict the 95% confidence intervals for these estimates. Methods A. Network meta-analysis (NMA) metho [file pmed.1004683.s001.docx]

**S1 Appendix**

Table of Contents

[Text A. Antimalarial treatment dosage 3](#_Toc204582167)

[Table A. Antimalarial treatment suppliers 6](#_Toc204582168)

[Fig A. Original PQ03 molecular gametocyte densities 7](#_Toc204582169)

[Table B. Baseline descriptives per treatment group 8](#_Toc204582170)

[Fig B. Relation between oocyst density and prevalence 9](#_Toc204582171)

[Table C. Relative reduction in gametocyte prevalence 10](#_Toc204582172)

[Fig C. Relative reduction in gametocyte prevalence per study arm (ungrouped) 11](#_Toc204582173)

[Methods A. Network meta-analysis (NMA) methodology and assessment of validity 12](#_Toc204582174)

[Fig D. Forest plots of treatment comparisons of reduction in gametocyte prevalence by microscopy at days 2, 7, and 14. All comparisons are with ACT-PQ. 13](#_Toc204582175)

[Fig E. Forest plots of treatment comparisons of reduction in gametocyte prevalence by RT-qPCR at days 2, 7, and 14. All comparisons are with ACT-PQ. 14](#_Toc204582176)

[Table D. Relative reduction in gametocyte density 15](#_Toc204582177)

[Fig F. Relative reduction in gametocyte density per study arm (ungrouped) 16](#_Toc204582178)

[Table E. Treatment comparisons of reduction in gametocyte density by microscopy at day 2. 17](#_Toc204582179)

[Table F. Treatment comparisons of reduction in gametocyte density by microscopy at day 7. 18](#_Toc204582180)

[Table G. Treatment comparisons of reduction in gametocyte density by microscopy at day 14. 19](#_Toc204582181)

[Table H. Treatment comparisons of reduction in gametocyte density by RT-qPCR at day 2. 20](#_Toc204582182)

[Table I. Treatment comparisons of reduction in gametocyte density by RT-qPCR at day 7. 21](#_Toc204582183)

[Table J. Treatment comparisons of reduction in gametocyte density by RT-qPCR at day 14. 22](#_Toc204582184)

[Fig G. Forest plots of treatment comparisons of reduction in gametocyte density by microscopy at days 2, 7, and 14. All comparisons are with ACT-PQ. 23](#_Toc204582185)

[Fig H. Forest plots of treatment comparisons of reduction in gametocyte density by RT-qPCR at days 2, 7, and 14. 24](#_Toc204582186)

[Table K. Relative reduction in proportion infected mosquitoes 25](#_Toc204582187)

[Fig I. Forest plots of treatment comparisons of reduction in proportion infected mosquitoes at days 2, 7, and 14. All comparisons are with ACT-PQ. 26](#_Toc204582188)

[Fig J. Relative reduction in proportion infected mosquitoes per study arm (ungrouped) 27](#_Toc204582189)

[Fig K. Relative reduction in proportion infected mosquitoes comparing the same study arms across different studies 28](#_Toc204582190)

[Fig L. Relative reduction in the probability of infecting at least 1 mosquito 29](#_Toc204582191)

[Table L. Relative reduction in the probability of infecting at least 1 mosquito 30](#_Toc204582192)

[Fig M. Relative reduction in the probability of infecting at least 1 mosquito per study arm (ungrouped) 31](#_Toc204582193)

[Fig N. Forest plots of treatment comparisons of probability of infecting at least 1 mosquito at days 2, 7, and 14. All comparisons are with ACT-PQ. 32](#_Toc204582194)

[Fig O. Relative reduction in oocyst density 33](#_Toc204582195)

[Table M. Relative reduction in oocyst density 34](#_Toc204582196)

[Fig P. Relative reduction in oocyst density per study arm (ungrouped) 35](#_Toc204582197)

[Fig Q. Forest plots of treatment comparisons of reduction in oocyst density at days 2, 7, and 14. All comparisons are with ACT-PQ. 36](#_Toc204582198)

[Table N. Kaplan–Meier survival data for time to clearance of infectivity and gametocytes, by treatment arm 37](#_Toc204582199)

[Table O. Hazard ratios for infectivity survival curves (adjusted by baseline PCR gametocyte densities) 41](#_Toc204582200)

[Table P. Hazard ratios for gametocytes by microscopy survival curves 42](#_Toc204582201)

[Table Q. Hazard ratios for gametocytes by RT-qPCR survival curves 43](#_Toc204582202)

[Fig R. Forest plots of treatment comparisons of infectivity survival curves (adjusted by baseline RT-qPCR gametocyte densities) at days 2, 7, and 14. All comparisons are with ACT-PQ. 44](#_Toc204582203)

[Fig S. Forest plots of treatment comparisons of microscopy gametocyte density survival curves at days 2, 7, and 14. All comparisons are with ACT-PQ. 45](#_Toc204582204)

[Fig T. Forest plots of treatment comparisons of molecular gametocyte density survival curves at days 2, 7, and 14. All comparisons are with ACT-PQ. 46](#_Toc204582205)

[Fig U. Consistency assessment comparing direct and indirect treatment effects for proportion infected mosquitoes 47](#_Toc204582206)

## Text A. Antimalarial treatment dosage

Dosing schedules for each treatment regimen.

*Sulphadoxine-Pyrimethamine plus Amodiaquine*

SP tablets containing 500 mg sulfadoxine and 25 mg pyrimethamine and AQ tablets containing 150 mg amodiaquine were administered according to weight as per manufacturer guidelines shown below:

| **Body weight** | **500/50 mg sulfadoxine/pyrimethamine tablet** | | |
| --- | --- | --- | --- |
|  | **Day 0** | **Day 1** | **Day 2** |
| **11 to 20 kg** | 1x 1 tablet | 1x 1 tablet | 1x 1 tablet |
| **21 to 30 kg** | 1x 1·5 tablets | 1x 1·5 tablets | 1x 1·5 tablets |
| **31 to 45 kg** | 1x 2 tablets | 1x 2 tablets | 1x 2 tablets |
| **> 45 kg** | 1x 3 tablets | 1x 3 tablets | 1x 3 tablets |
| **Body weight** | **150 mg amodiaquine tablet** | | |
|  | **Day 0** | **Day 1** | **Day 2** |
| **15 to 18 kg** | 1x 1·5 tablets | 1x 1 tablet | 1x 1 tablet |
| **19 to 24 kg** | 1x 1·5 tablets | 1x 1·5 tablets | 1x 1·5 tablets |
| **25 to 35 kg** | 1x 2·5 tablets | 1x 2·5 tablets | 1x 2 tablets |
| **36 to 50 kg** | 1x 3 tablets | 1x 3 tablets | 1x 3 tablets |
| **> 50 kg** | 1x 4 tablets | 1x 4 tablets | 1x 3 tablets |

*Artemether-lumefantrine*

AL treatment tablets containing 20/120 mg artemether/lumefantrine or 80/480 mg artemether/lumefantrine were administered according to weight as per manufacturer guidelines shown below:

| **Body weight (kg)** | **20/120 mg artemether/lumefantrine tablet** | | | **80/480 mg artemether/lumefantrine tablet** | | |
| --- | --- | --- | --- | --- | --- | --- |
|  | **Day 0** | **Day 1** | **Day 2** | **Day 0** | **Day 1** | **Day 2** |
| **5 to < 15 kg** | 2x 1 tablet | 2x 1 tablet | 2x 1 tablet | - | - | - |
| **15 to < 25 kg** | 2x 2 tablets | 2x 2 tablets | 2x 2 tablets | - | - | - |
| **25 to < 35 kg** | 2x 3 tablets | 2x 3 tablets | 2x 3 tablets | - | - | - |
| **≥ 35 kg** | 2x 4 tablets | 2x 4 tablets | 2x 4 tablets | 2x 1 tablet | 2x 1 tablet | 2x 1 tablet |

*Dihydroartemisinin-Piperaquine*

Treatment tablets containing 160/320 mg piperaquine with 20/40 mg dihydroartemisinin tablets were administered according to weight as per manufacturer guidelines shown below:

| **Body weight (kg)** | **Total daily dose (mg)**  **(1x/day for 3 days)** | | **Tablet strength and number of tablets per dose** |
| --- | --- | --- | --- |
|  | Piperaquine | DHA |  |
| 5 to <7 | 80 | 10 | ½ x 160mg / 20mg |
| 7 to <13 | 160 | 20 | 1 x 160mg / 20mg |
| 13 to <24 | 320 | 40 | 1 x 320mg / 40mg |
| 24 to <36 | 640 | 80 | 2 x 320mg / 40mg |
| 36 to <75 | 960 | 120 | 3 x 320mg / 40mg |
| 75 to 80 | 1,280 | 160 | 4 x 320mg / 40mg |
| >80 | Not eligible | | |

*Artesunate-Amodiaquine*

Tablets contained 50mg/135 mg or 100mg/270 mg of artesunate/amodiaquine and were administered according to manufacturer guidelines, as shown below:

| **Weight** | **Tablets** | **D0** | **D1** | **D2** |
| --- | --- | --- | --- | --- |
| 9 to < 18 kg | 50 mg AS/135 mg AQ base | 1 tab | 1 tab | 1 tab |
| 18 to < 36 kg | 100 mg AS/270 mg AQ base | 1 tab | 1 tab | 1 tab |
|  | blister pack of 3 tab |  |  |  |
| ≥ 36 kg | 100 mg AS/270 mg AQ base | 2 tab | 2 tab | 2 tab |
|  | blister pack of 6 tab |  |  |  |

*Pyronaridine-Artesunate*

PY-AS granules containing 60 mg pyronaridine-tetraphosphate/20mg artesunate were administered to children <20kg, and PY-AS tablets containing 180 mg pyronaridine-tetraphosphate/60mg artesunate were administered to children and adults >20kg, according to weight as per manufacturer guidelines shown below:

| **Granules (Children <20kg)** | | | |
| --- | --- | --- | --- |
| **Body weight (kg)** | **Total daily dose (mg)**  **(1x/day for 3 days)** | | **Sachet strength and number of tablets per dose** |
|  | Pyronaridine-tetraphosphate | Artesunate |  |
| 5 - <8kg | 60 | 20 | 1 x 60mg/20mg |
| 8 - <15kg | 120 | 40 | 2 x 60mg/20mg |
| 15 - <20kg | 180 | 60 | 3 x 60mg/20mg |

| **Tablets (Children and adults >20kg)** | | | |
| --- | --- | --- | --- |
| **Body weight (kg)** | **Total daily dose (mg)**  **(1x/day for 3 days)** | | **Tablet strength and number of tablets per dose** |
|  | Pyronaridine-tetraphosphate | Artesunate |  |
| 20 - <24kg | 180 | 60 | 1 x 60mg/20mg |
| 24 - <45kg | 360 | 120 | 2 x 60mg/20mg |
| 45-<65kg | 540 | 180 | 3 x 60mg/20mg |
| >65kg | 720 | 240 | 4 x 180mg/60mg |

*Primaquine*

Primaquine tablets were dissolved to a 1 mg/mL solution in distilled water and administered orally to the nearest mL, according to bodyweight at 0·25 mg/kg. Primaquine was administered as a single dose immediately after the first dose of ACT.

*Tafenoquine*

100mg Tafenoquine tablets were available for this study, and were prepared into a 1mg/mL solution in water for weight-based dosing in 5 kg bands as follows:

**1.66 mg/kg Tafenoquine**

| **Weight min** | **Weight max** | **TQ 1mg/mL total (mL)** | **Water (mL)** | **Masking solution (mL)** |
| --- | --- | --- | --- | --- |
| 30 | 35 | 54.0 | 136.1 | 10 |
| 35.01 | 40 | 62.3 | 127.7 | 10 |
| 40.01 | 45 | 70.6 | 119.4 | 10 |
| 45.01 | 50 | 78.9 | 111.1 | 10 |
| 50.01 | 55 | 87.2 | 102.8 | 10 |
| 55.01 | 60 | 95.5 | 94.5 | 10 |
| 60.01 | 65 | 103.8 | 86.2 | 10 |
| 65.01 | 70 | 112.1 | 77.9 | 10 |
| 70.01 | 75 | 120.4 | 69.6 | 10 |
| 75.01 | 80 | 128.7 | 61.3 | 10 |

**0.83 mg/kg Tafenoquine**

| **Weight min** | **Weight max** | **TQ 1mg/mL total (mL)** | **Water (mL)** | **Masking solution (mL)** |
| --- | --- | --- | --- | --- |
| 30 | 35 | 27.0 | 163.0 | 10 |
| 35.01 | 40 | 31.1 | 158.9 | 10 |
| 40.01 | 45 | 35.3 | 154.7 | 10 |
| 45.01 | 50 | 39.4 | 150.6 | 10 |
| 50.01 | 55 | 43.6 | 146.4 | 10 |
| 55.01 | 60 | 47.7 | 142.3 | 10 |
| 60.01 | 65 | 51.9 | 138.1 | 10 |
| 65.01 | 70 | 56.0 | 134.0 | 10 |
| 70.01 | 75 | 60.2 | 129.8 | 10 |
| 75.01 | 80 | 64.3 | 125.7 | 10 |

###

## Table A. Antimalarial treatment suppliers

| **Study** | **Study drug** | **Supplier** |
| --- | --- | --- |
| **PQ01** | Primaquine | Sanofi, Laval, QC, Canada |
|  | Dihydroartemisinin-piperaquine (Eurartesim) | Sigma-Tau, Pomezia, Italy |
| **PQ03** | Primaquine | Sanofi, Laval, QC, Canada |
|  | Sulfadoxine-pyrimethamine (Fansidar) | Guilin Pharmaceutical, Shanghai, China |
|  | Amodiaquine | Guilin Pharmaceutical, Shanghai, China |
|  | Dihydroartemisinin-piperaquine (Eurartesim) | Sigma-Tau, Pomezia, Italy |
| **NECTAR1** | Pyronaridine-artesunate (Pyramax) | Shin Poong Pharmaceutical, Seoul, South Korea |
|  | Primaquine | ACE Pharmaceuticals, Zeewolde, the Netherlands |
|  | Dihydroartemisinin-piperaquine (Eurartesim) | Sigma Tau, Gaithersburg, MD, USA |
| **NECTAR2** | Dihydroartemisinin-piperaquine (Eurartesim) | Sigma Tau, Gaithersburg, MD, USA |
|  | Tafenoquine | 60° Pharmaceuticals Ltd, USA |
| **NECTAR3** | Artemether-lumefantrine (Coartem) | Novartis, Basel, Switzerland |
|  | Primaquine | ACE Pharmaceuticals, Zeewolde, the Netherlands |
|  | Sulfadoxine-pyrimethamine plus amodiaquine | Guilin Pharmaceutical, Shanghai, China |
|  | Tafenoquine | 60° Pharmaceuticals Ltd, USA |
| **NECTAR4** | Artemether-lumefantrine | Guilin Pharmaceutical, Shanghai, China |
|  | Amodiaquine | Guilin Pharmaceutical, Shanghai, China |
|  | Primaquine | ACE Pharmaceuticals, Zeewolde, The Netherlands |
|  | Artesunate-amodiaquine | Guilin Pharmaceutical, Shanghai, China |

Suppliers of study drugs used in each study

## Fig A. Original PQ03 molecular gametocyte densities

Bland-Altman plot presenting the agreement between gametocyte density measured by microscopy (x-axis) and RT-qPCR (y-axis) pre-treatment. The original PQ03 (2016) RT-qPCR data is presented here, which showed that the comparison of densities measured by both methods for this study was an outlier compared to the other studies. Following this, the molecular gametocyte quantification was repeated for this study.

The solid black line indicates the overall mean difference including all studies, and dotted lines represent 1.96 x standard deviation of the differences. The dashed lines, coloured by study, represent group-specific mean differences. Only in 2016 (PQ03 study), there was a relevant difference between the two gametocyte measurements; molecular assays were repeated for this sample set prior to inclusion in the analyses.

## Table B. Baseline descriptives per treatment group

| **Treatment** | **N (%)** | **Males** | **Age** | **Temperature** | **Total parasites/uL** | **Gametocytes/uL (microscopy)** | **Gametocytes/uL (pcr)** | **Female gametocytes/uL (pcr)** | **Proportion infected** | **Gametocyte prevalence** | **Asexual parasite prevalence** | **Proportion participants infectious** |
| --- | --- | --- | --- | --- | --- | --- | --- | --- | --- | --- | --- | --- |
| **DHA-PPQ** | 79 (18.8%) | 59 (74.7%) | 16.5 (10.3) | 36.5 (0.4) | 224.0 (76.0 - 840.0) | 48.0 (16.0 - 80.0) | 80.3 (35.6 - 128.4) | 60.0 (20.8 - 115.7) | 3.0 (0.0 - 14.7) | 77 (97.5%) | 53 (67.1%) | 50 (63.3%) |
| **SP-AQ** | 40 (9.5%) | 29 (72.5%) | 17.2 (11.4) | 36.6 (0.5) | 288.0 (106.0 - 868.0) | 48.0 (32.0 - 112.0) | 65.2 (30.3 - 183.3) | 44.5 (15.1 - 129.2) | 5.2 (0.0 - 10.4) | 40 (100.0%) | 26 (65.0%) | 28 (70.0%) |
| **PY-AS** | 25 (5.9%) | 16 (64.0%) | 13.0 (7.7) | 36.5 (0.6) | 216.0 (64.0 - 704.0) | 64.0 (32.0 - 96.0) | 83.2 (35.9 - 121.7) | 46.0 (25.6 - 74.5) | 3.0 (0.0 - 13.7) | 25 (100.0%) | 13 (52.0%) | 17 (68.0%) |
| **AS-AQ** | 20 (4.7%) | 9 (45.0%) | 14.4 (6.3) | 36.4 (0.3) | 386.3 (162.0 - 1498.4) | 79.0 (41.5 - 232.1) | 52.5 (34.2 - 107.3) | 28.1 (11.3 - 38.9) | 3.9 (1.6 - 17.9) | 20 (100.0%) | 8 (40.0%) | 17 (85.0%) |
| **AL** | 60 (14.2%) | 27 (45.0%) | 18.9 (11.1) | 36.5 (0.4) | 154.5 (48.0 - 411.0) | 48.0 (31.7 - 124.8) | 32.2 (12.8 - 95.8) | 15.6 (6.6 - 38.6) | 3.1 (0.0 - 11.7) | 60 (100.0%) | 29 (48.3%) | 35 (58.3%) |
| **Non-ACT-PQ** | 20 (4.7%) | 20 (100.0%) | 10.3 (3.6) | 36.8 (0.4) | 112.0 (80.0 - 233.3) | 72.0 (32.0 - 96.0) | 198.8 (63.3 - 435.7) | 171.0 (55.1 - 332.6) | 21.6 (4.3 - 33.1) | 20 (100.0%) | 12 (60.0%) | 19 (95.0%) |
| **ACT-PQ** | 138 (32.7%) | 81 (58.7%) | 15.0 (10.0) | 36.4 (0.4) | 328.0 (96.0 - 1473.9) | 70.5 (32.0 - 144.0) | 66.3 (24.8 - 165.6) | 35.5 (12.7 - 109.3) | 5.2 (0.0 - 16.3) | 138 (100.0%) | 83 (60.1%) | 96 (69.6%) |
| **ACT-TQ** | 40 (9.5%) | 28 (70.0%) | 20.2 (10.9) | 36.5 (0.4) | 156.0 (64.0 - 1092.0) | 48.0 (32.0 - 64.0) | 45.2 (19.1 - 204.2) | 22.3 (11.0 - 88.5) | 5.2 (0.0 - 18.8) | 40 (100.0%) | 23 (57.5%) | 28 (70.0%) |

Study participants characteristics, parasite densities and infectivity prior to treatment.

## Fig B. Relation between oocyst density and prevalence

Association between oocyst prevalence and oocyst density, determined by a mixed-effects logistic regression with random effect for study, where the solid black line represents the association averaged over studies.

## Table C. Relative reduction in gametocyte prevalence

| **Treatment** | **Microscopy** | | | **RT-qPCR** | | |
| --- | --- | --- | --- | --- | --- | --- |
|  | **Day 2** | **Day 7** | **Day 14** | **Day 2** | **Day 7** | **Day 14** |
| **DHA-PPQ** | 8.45% (-28.72%, 34.89%), p=0.6115 | 20.20% (-14.17%, 44.23%), p=0.2169 | 44.50% (16.55%, 63.08%), p=0.0047 | 0.00% (-37.01%, 27.01%), p>0.9999 | 0.00% (-38.22%, 27.65%), p>0.9999 | 0.00% (-37.74%, 27.40%), p>0.9999 |
| **SP-AQ** | 15.00% (-34.27%, 46.19%), p=0.4860 | 26.32% (-19.43%, 54.54%), p=0.2152 | 38.46% (-2.08%, 62.90%), p=0.0601 | 0.00% (-58.98%, 37.10%), p>0.9999 | 0.00% (-58.98%, 37.10%), p>0.9999 | 0.00% (-59.42%, 37.27%), p>0.9999 |
| **PY-AS** | 8.00% (-62.08%, 47.78%), p=0.7729 | 20.00% (-44.03%, 55.57%), p=0.4570 | 44.00% (-7.73%, 70.89%), p=0.0824 | 0.00% (-78.24%, 43.90%), p>0.9999 | 0.00% (-77.17%, 43.56%), p>0.9999 | 4.17% (-70.82%, 46.23%), p=0.8852 |
| **AS-AQ** | 5.00% (-78.00%, 49.30%), p=0.8728 | 10.00% (-70.14%, 52.39%), p=0.7457 | 31.58% (-37.55%, 65.96%), p=0.2868 | 0.00% (-85.86%, 46.20%), p>0.9999 | 0.00% (-85.86%, 46.20%), p>0.9999 | 5.26% (-79.09%, 49.89%), p=0.8678 |
| **AL** | 31.03% (-2.89%, 53.77%), p=0.0687 | 50.88% (23.07%, 68.63%), p=0.0019 | 75.44% (56.06%, 86.27%), p<0.0001 | 0.00% (-43.46%, 30.30%), p>0.9999 | 0.00% (-43.70%, 30.41%), p>0.9999 | 5.26% (-36.83%, 34.41%), p=0.7732 |
| **Non-ACT-PQ** | 0.00% (-84.48%, 45.79%), p>0.9999 | 68.42% (21.76%, 87.25%), p=0.0128 | 94.44% (58.70%, 99.25%), p=0.0047 | 0.00% (-90.54%, 47.52%), p>0.9999 | 0.00% (-90.54%, 47.52%), p>0.9999 | 55.56% (-2.22%, 80.67%), p=0.0563 |
| **ACT-PQ** | 19.63% (-5.21%, 38.60%), p=0.1118 | 90.57% (82.08%, 95.03%), p<0.0001 | 98.11% (92.38%, 99.53%), p<0.0001 | 0.00% (-27.06%, 21.30%), p>0.9999 | 42.24% (22.61%, 56.89%), p=0.0002 | 70.54% (56.90%, 79.86%), p<0.0001 |
| **ACT-TQ** | 27.50% (-16.93%, 55.05%), p=0.1873 | 61.54% (30.38%, 78.75%), p=0.0016 | 89.19% (69.78%, 96.13%), p<0.0001 | 0.00% (-55.44%, 35.67%), p>0.9999 | 0.00% (-56.32%, 36.03%), p>0.9999 | 25.00% (-22.51%, 54.09%), p=0.2505 |

Relative reduction compared to baseline in gametocyte prevalence by microscopy and RT-qPCR, at three time points (Day 2, Day 7, Day 14), with 95% confidence intervals.

## Fig C. Relative reduction in gametocyte prevalence per study arm (ungrouped)

Bar charts illustrating the relative reduction compared to baseline in gametocyte prevalence by microscopy and RT-qPCR for each study arm (ungrouped), over three time points (Day 2, Day 7, Day 14). Vertical bars depict the 95% confidence intervals for these estimates.

**
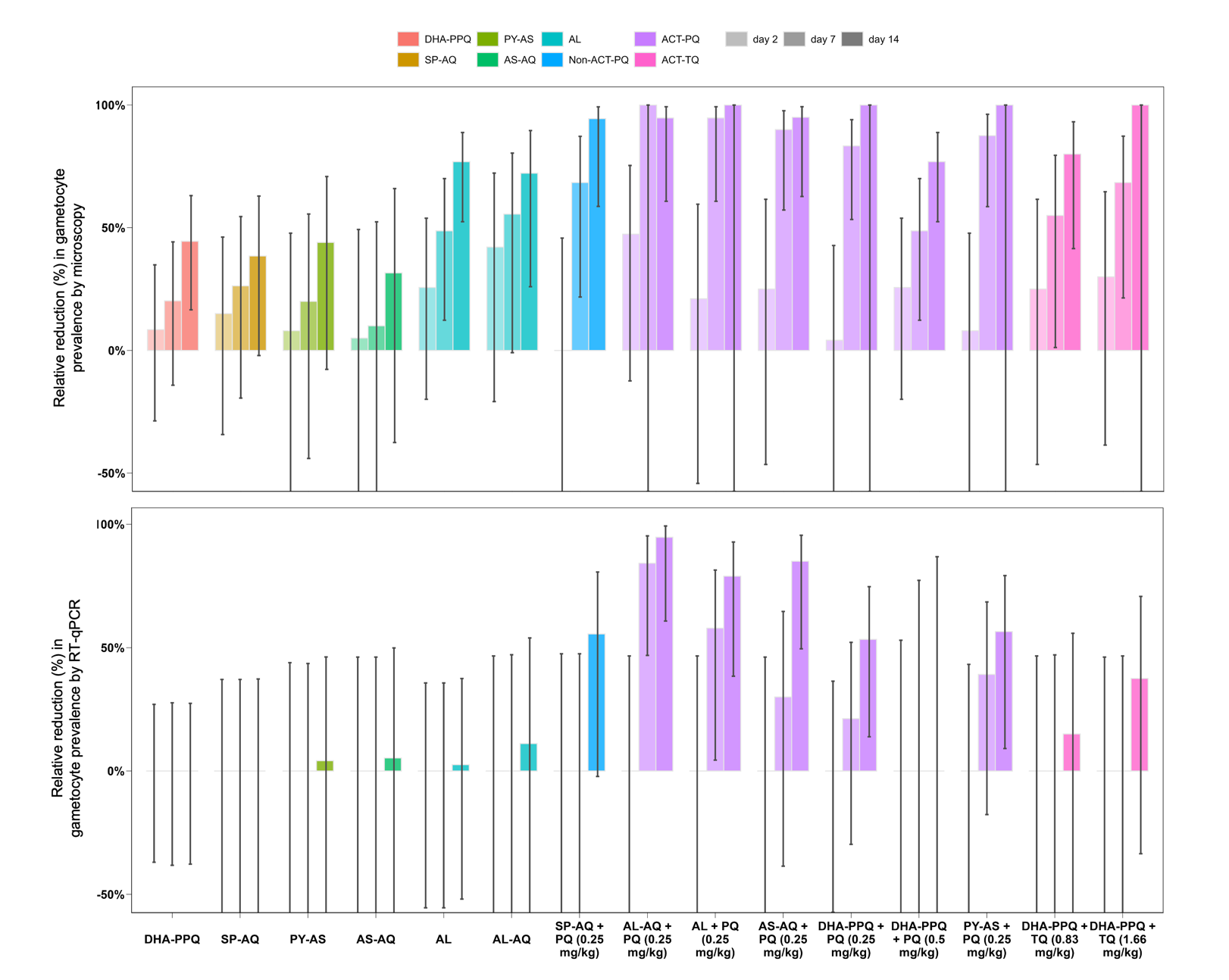
**

## Methods A. Network meta-analysis (NMA) methodology and assessment of validity

This section outlines the NMA, which integrated direct and indirect comparisons across studies to estimate relative treatment effects. It details the assessment of key NMA assumptions, transitivity and consistency, including the comparability of participant characteristics and outcomes across studies, and the agreement between direct and indirect treatment effects.

In network meta-analysis, transitivity means that you can validly compare treatments indirectly (e.g., A vs C through B) because the different comparisons are similar in distribution of effect modifiers across studies. The assumption of transitivity in the NMA was supported by the overall similarity in participant characteristics and baseline transmission potential across the included studies. All participants were microscopically confirmed gametocyte carriers at enrolment, and the distribution of gametocyte densities and infectivity to mosquitoes was broadly comparable across trials, with the exception of one study (PQ01) that included clinical malaria cases and showed higher baseline parasite densities. Despite this, the relationship between gametocyte density and mosquito infectivity was consistent across studies. Importantly, for longitudinal outcomes, primary endpoints were defined as relative reductions from each individual's baseline, which helped minimize the impact of baseline heterogeneity across studies. This approach further supports the assumption of transitivity within the treatment comparison network.

In NMA, consistency refers to the agreement between direct and indirect treatment comparisons. When consistency holds, the effect estimate obtained by comparing two treatments directly is similar to that obtained indirectly through a common comparator. Visual assessment of consistency indicated that direct and indirect estimates were largely aligned, as evidenced by overlapping confidence intervals. (supplementary figure 21, appendix 1, p 43). This indicates that the assumption of consistency was reasonably maintained across the treatment networks included in the analysis.

## Fig D. Forest plots of treatment comparisons of reduction in gametocyte prevalence by microscopy at days 2, 7, and 14. All comparisons are with ACT-PQ.


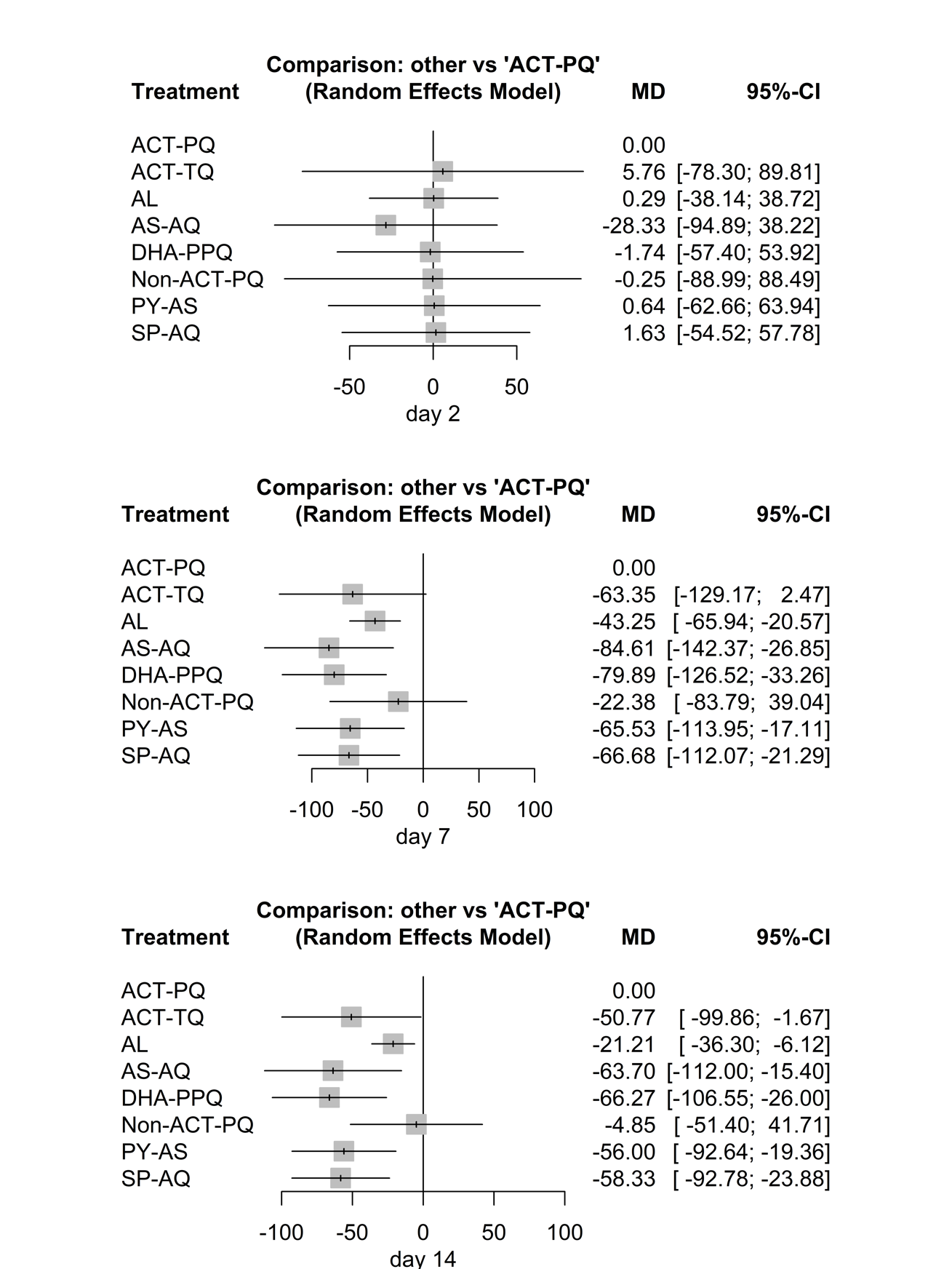


Results from the network meta-analysis are shown as mean differences (MD) in relative reductions from baseline in gametocyte prevalence by microscopy, with 95% confidence intervals. Each treatment is compared to ACT-PQ, the reference treatment. Negative values indicate a smaller reduction than ACT-PQ, while positive values indicate a larger reduction. Point estimates are plotted as squares proportional to study weight, and horizontal lines denote confidence intervals.

Fig E. Forest plots of treatment comparisons of reduction in gametocyte prevalence by RT-qPCR at days 2, 7, and 14. All comparisons are with ACT-PQ.

Results from the network meta-analysis are shown as mean differences (MD) in relative reductions from baseline in gametocyte prevalence by RT-qPCR, with 95% confidence intervals. Each treatment is compared to ACT-PQ, the reference treatment. Negative values indicate a smaller reduction than ACT-PQ, while positive values indicate a larger reduction. Point estimates are plotted as squares proportional to study weight, and horizontal lines denote confidence intervals.

**
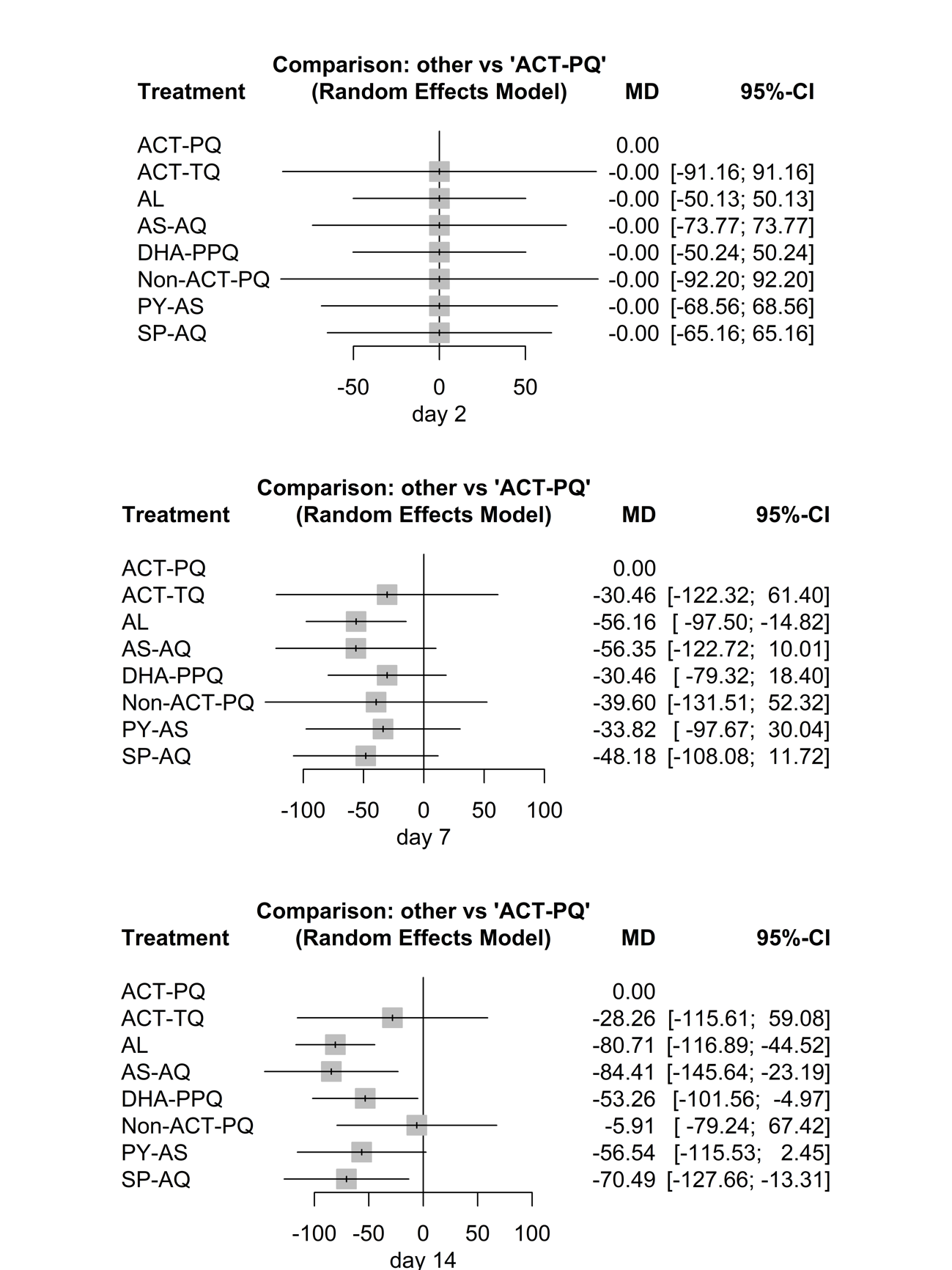
**

## Table D. Relative reduction in gametocyte density

| **Treatment** | **Microscopy** | | | **RT-qPCR** | | |
| --- | --- | --- | --- | --- | --- | --- |
|  | **Day 2** | **Day 7** | **Day 14** | **Day 2** | **Day 7** | **Day 14** |
| **DHA-PPQ** | 38.56% (-12.82%, 66.54%), p=0.1162 | 76.45% (56.49%, 87.26%), p<0.0001 | 94.84% (90.40%, 97.22%), p<0.0001 | 19.13% (-13.35%, 42.30%), p=0.2177 | 54.07% (34.95%, 67.57%), p<0.0001 | 73.45% (62.55%, 81.18%), p<0.0001 |
| **SP-AQ** | 36.17% (-42.00%, 71.31%), p=0.2711 | 75.47% (44.74%, 89.11%), p=0.0007 | 91.82% (81.68%, 96.34%), p<0.0001 | 24.29% (-25.14%, 54.19%), p=0.2778 | 51.74% (20.20%, 70.82%), p=0.0045 | 70.30% (50.80%, 82.07%), p<0.0001 |
| **PY-AS** | 58.86% (-13.10%, 85.04%), p=0.0852 | 82.89% (52.96%, 93.78%), p=0.0006 | 96.17% (89.47%, 98.61%), p<0.0001 | 15.84% (-57.08%, 54.91%), p=0.5880 | 55.12% (16.95%, 75.75%), p=0.0107 | 75.99% (55.58%, 87.03%), p<0.0001 |
| **AS-AQ** | 38.28% (-91.21%, 80.08%), p=0.4029 | 66.54% (-3.65%, 89.20%), p=0.0577 | 92.30% (75.73%, 97.56%), p<0.0001 | 25.76% (-43.79%, 61.67%), p=0.3771 | 65.68% (33.53%, 82.28%), p=0.0015 | 82.86% (66.43%, 91.25%), p<0.0001 |
| **AL** | 85.47% (71.88%, 92.49%), p<0.0001 | 96.43% (93.07%, 98.16%), p<0.0001 | 99.30% (98.65%, 99.64%), p<0.0001 | 51.05% (27.92%, 66.76%), p=0.0003 | 75.02% (63.13%, 83.07%), p<0.0001 | 93.39% (90.25%, 95.52%), p<0.0001 |
| **Non-ACT-PQ** | -38.40% (-323.74%, 54.79%), p=0.5692 | 99.03% (96.98%, 99.69%), p<0.0001 | 99.77% (99.27%, 99.93%), p<0.0001 | 31.73% (-37.66%, 66.15%), p=0.2861 | 99.08% (98.14%, 99.54%), p<0.0001 | 99.98% (99.96%, 99.99%), p<0.0001 |
| **ACT-PQ** | 78.12% (65.02%, 86.32%), p<0.0001 | 99.78% (99.65%, 99.86%), p<0.0001 | 99.85% (99.76%, 99.91%), p<0.0001 | 58.23% (45.98%, 67.70%), p<0.0001 | 99.79% (99.72%, 99.84%), p<0.0001 | 99.95% (99.93%, 99.96%), p<0.0001 |
| **ACT-TQ** | 80.21% (55.97%, 91.10%), p<0.0001 | 98.16% (95.88%, 99.18%), p<0.0001 | 99.61% (99.11%, 99.83%), p<0.0001 | 31.37% (-9.95%, 57.16%), p=0.1174 | 83.87% (73.95%, 90.02%), p<0.0001 | 99.45% (99.11%, 99.66%), p<0.0001 |

Relative reduction compared to baseline in gametocyte density by microscopy and RT-qPCR, at three time points (Day 2, Day 7, Day 14), with 95% confidence intervals.

Fig F. Relative reduction in gametocyte density per study arm (ungrouped)**
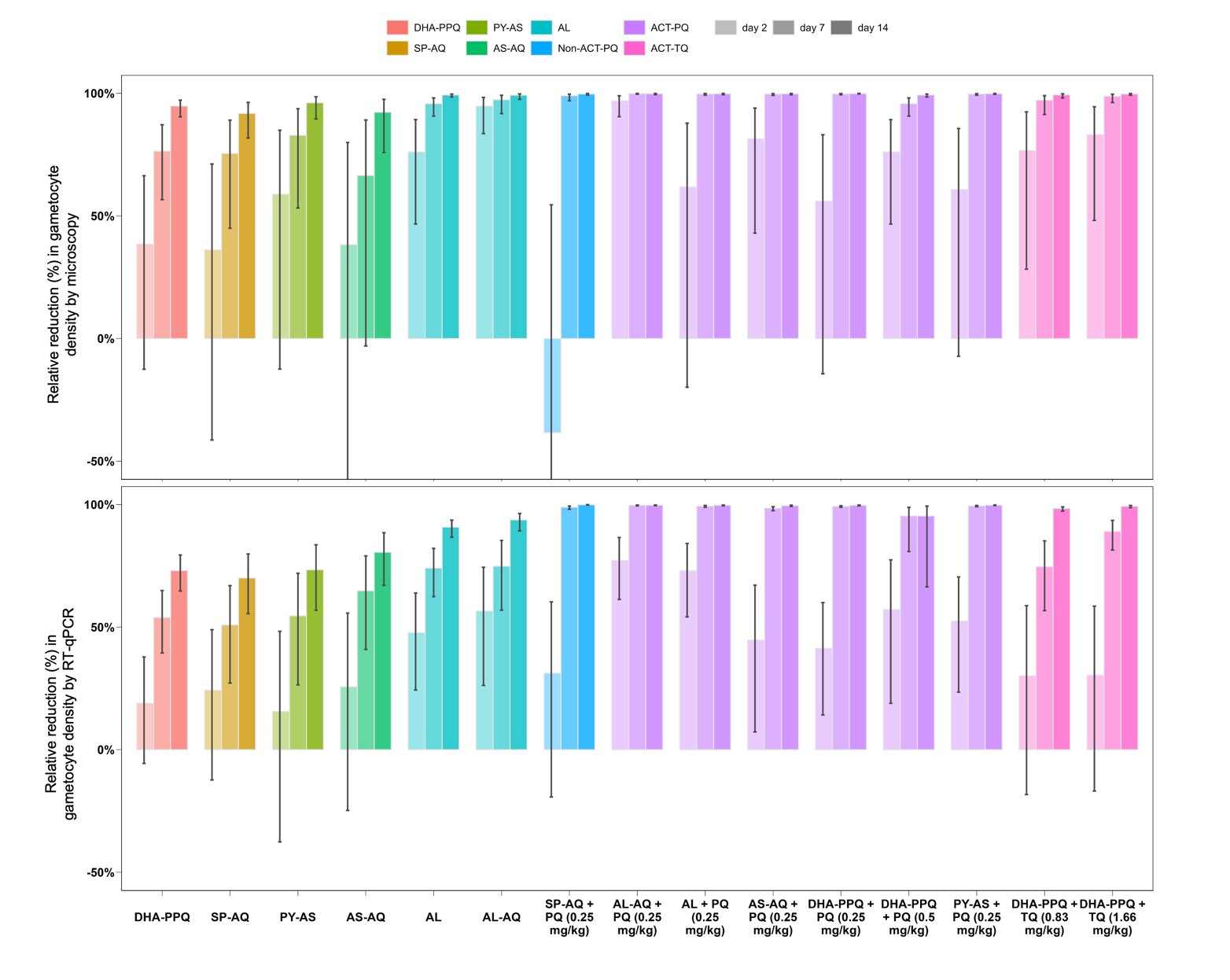
**

Bar charts illustrating the relative reduction compared to baseline in gametocyte prevalence by microscopy and RT-qPCR for each study arm (ungrouped), over three time points (Day 2, Day 7, Day 14). Vertical bars depict the 95% confidence intervals for these estimates.

## Table E. Treatment comparisons of reduction in gametocyte density by microscopy at day 2.

| reference | DHA-PPQ | SP-AQ | PY-AS | AS-AQ | AL | Non-ACT-PQ | ACT-PQ | ACT-TQ |
| --- | --- | --- | --- | --- | --- | --- | --- | --- |
| DHA-PPQ |  | -1.83% (-108.05%, 104.39%), p=0.9731 | 23.58% (-61.38%, 108.55%), p=0.5864 | -31.07% (-146.36%, 84.23%), p=0.5974 | 19.56% (-58.26%, 97.38%), p=0.6223 | -58.20% (-285.86%, 169.46%), p=0.6164 | 22.63% (-54.16%, 99.41%), p=0.5636 | 16.78% (-37.61%, 71.17%), p=0.5454 |
| SP-AQ | 1.83% (-104.39%, 108.05%), p=0.9731 |  | 25.41% (-77.72%, 128.54%), p=0.6291 | -29.24% (-148.38%, 89.90%), p=0.6305 | 21.39% (-61.05%, 103.83%), p=0.6111 | -56.37% (-266.76%, 154.02%), p=0.5995 | 24.46% (-58.38%, 107.29%), p=0.5628 | 18.61% (-100.72%, 137.95%), p=0.7599 |
| PY-AS | -23.58% (-108.55%, 61.38%), p=0.5864 | -25.41% (-128.54%, 77.72%), p=0.6291 |  | -54.65% (-162.02%, 52.72%), p=0.3185 | -4.03% (-69.64%, 61.59%), p=0.9043 | -81.78% (-310.86%, 147.30%), p=0.4841 | -0.96% (-65.18%, 63.27%), p=0.9767 | -6.80% (-107.69%, 94.08%), p=0.8949 |
| AS-AQ | 31.07% (-84.23%, 146.36%), p=0.5974 | 29.24% (-89.90%, 148.38%), p=0.6305 | 54.65% (-52.72%, 162.02%), p=0.3185 |  | 50.63% (-35.89%, 137.14%), p=0.2514 | -27.13% (-264.72%, 210.47%), p=0.8229 | 53.69% (-32.36%, 139.75%), p=0.2214 | 47.85% (-79.63%, 175.33%), p=0.4619 |
| AL | -19.56% (-97.38%, 58.26%), p=0.6223 | -21.39% (-103.83%, 61.05%), p=0.6111 | 4.03% (-61.59%, 69.64%), p=0.9043 | -50.63% (-137.14%, 35.89%), p=0.2514 |  | -77.75% (-299.29%, 143.79%), p=0.4915 | 3.07% (-10.63%, 16.77%), p=0.6606 | -2.78% (-97.72%, 92.17%), p=0.9543 |
| Non-ACT-PQ | 58.20% (-169.46%, 285.86%), p=0.6164 | 56.37% (-154.02%, 266.76%), p=0.5995 | 81.78% (-147.30%, 310.86%), p=0.4841 | 27.13% (-210.47%, 264.72%), p=0.8229 | 77.75% (-143.79%, 299.29%), p=0.4915 |  | 80.82% (-140.78%, 302.43%), p=0.4747 | 74.98% (-159.09%, 309.05%), p=0.5301 |
| ACT-PQ | -22.63% (-99.41%, 54.16%), p=0.5636 | -24.46% (-107.29%, 58.38%), p=0.5628 | 0.96% (-63.27%, 65.18%), p=0.9767 | -53.69% (-139.75%, 32.36%), p=0.2214 | -3.07% (-16.77%, 10.63%), p=0.6606 | -80.82% (-302.43%, 140.78%), p=0.4747 |  | -5.85% (-99.94%, 88.25%), p=0.9031 |
| ACT-TQ | -16.78% (-71.17%, 37.61%), p=0.5454 | -18.61% (-137.95%, 100.72%), p=0.7599 | 6.80% (-94.08%, 107.69%), p=0.8949 | -47.85% (-175.33%, 79.63%), p=0.4619 | 2.78% (-92.17%, 97.72%), p=0.9543 | -74.98% (-309.05%, 159.09%), p=0.5301 | 5.85% (-88.25%, 99.94%), p=0.9031 |  |

Results from the network meta-analysis are shown as the absolute difference between treatment groups in the relative reduction from baseline in gametocyte density by microscopy at day 2, with 95% confidence intervals and corresponding p-values. For example, the absolute difference between DHA-PPQ and AL in the relative reduction in microscopical gametocyte density at day 2 is -19.56% (-97.38%, 58.26%), and the difference between these arms (19.56% lower reduction for DHA-PPQ) is not statistically significant (p=0.6223).

## Table F. Treatment comparisons of reduction in gametocyte density by microscopy at day 7.

| reference | DHA-PPQ | SP-AQ | PY-AS | AS-AQ | AL | Non-ACT-PQ | ACT-PQ | ACT-TQ |
| --- | --- | --- | --- | --- | --- | --- | --- | --- |
| DHA-PPQ |  | 20.17% (-34.99%, 75.33%), p=0.4736 | 34.35% (-17.07%, 85.77%), p=0.1905 | 17.91% (-48.06%, 83.88%), p=0.5946 | 47.87% (0.96%, 94.78%), p=0.0455 | 35.29% (-19.69%, 90.28%), p=0.2084 | 51.19% (4.36%, 98.01%), p=0.0321 | 5.13% (-4.73%, 15.00%), p=0.3077 |
| SP-AQ | -20.17% (-75.33%, 34.99%), p=0.4736 |  | 14.18% (-39.22%, 67.58%), p=0.6027 | -2.26% (-69.78%, 65.27%), p=0.9478 | 27.70% (-21.37%, 76.77%), p=0.2685 | 15.13% (-5.39%, 35.64%), p=0.1484 | 31.02% (-17.97%, 80.01%), p=0.2145 | -15.03% (-71.07%, 41.00%), p=0.5990 |
| PY-AS | -34.35% (-85.77%, 17.07%), p=0.1905 | -14.18% (-67.58%, 39.22%), p=0.6027 |  | -16.44% (-67.54%, 34.67%), p=0.5284 | 13.52% (-7.93%, 34.97%), p=0.2167 | 0.95% (-54.17%, 56.06%), p=0.9732 | 16.84% (-4.42%, 38.10%), p=0.1205 | -29.21% (-81.57%, 23.14%), p=0.2741 |
| AS-AQ | -17.91% (-83.88%, 48.06%), p=0.5946 | 2.26% (-65.27%, 69.78%), p=0.9478 | 16.44% (-34.67%, 67.54%), p=0.5284 |  | 29.96% (-16.60%, 76.52%), p=0.2073 | 17.38% (-51.50%, 86.27%), p=0.6209 | 33.28% (-13.19%, 79.75%), p=0.1605 | -12.78% (-79.48%, 53.93%), p=0.7073 |
| AL | -47.87% (-94.78%, -0.96%), p=0.0455 | -27.70% (-76.77%, 21.37%), p=0.2685 | -13.52% (-34.97%, 7.93%), p=0.2167 | -29.96% (-76.52%, 16.60%), p=0.2073 |  | -12.58% (-63.50%, 38.35%), p=0.6284 | 3.32% (0.44%, 6.20%), p=0.0238 | -42.74% (-90.67%, 5.20%), p=0.0806 |
| Non-ACT-PQ | -35.29% (-90.28%, 19.69%), p=0.2084 | -15.13% (-35.64%, 5.39%), p=0.1484 | -0.95% (-56.06%, 54.17%), p=0.9732 | -17.38% (-86.27%, 51.50%), p=0.6209 | 12.58% (-38.35%, 63.50%), p=0.6284 |  | 15.89% (-34.95%, 66.74%), p=0.5401 | -30.16% (-86.03%, 25.71%), p=0.2900 |
| ACT-PQ | -51.19% (-98.01%, -4.36%), p=0.0321 | -31.02% (-80.01%, 17.97%), p=0.2145 | -16.84% (-38.10%, 4.42%), p=0.1205 | -33.28% (-79.75%, 13.19%), p=0.1605 | -3.32% (-6.20%, -0.44%), p=0.0238 | -15.89% (-66.74%, 34.95%), p=0.5401 |  | -46.06% (-93.91%, 1.80%), p=0.0592 |
| ACT-TQ | -5.13% (-15.00%, 4.73%), p=0.3077 | 15.03% (-41.00%, 71.07%), p=0.5990 | 29.21% (-23.14%, 81.57%), p=0.2741 | 12.78% (-53.93%, 79.48%), p=0.7073 | 42.74% (-5.20%, 90.67%), p=0.0806 | 30.16% (-25.71%, 86.03%), p=0.2900 | 46.06% (-1.80%, 93.91%), p=0.0592 |  |

Results from the network meta-analysis are shown as the absolute difference between treatment groups in the relative reduction from baseline in gametocyte density by microscopy at day 7, with 95% confidence intervals and corresponding p-values. For example, the absolute difference between DHA-PPQ and AL in the relative reduction in microscopical gametocyte density at day 7 is -47.87% (-94.78%, -0.96%), and the difference between these arms (47.87% lower reduction for DHA-PPQ) is statistically significant (p=0.0455).

## Table G. Treatment comparisons of reduction in gametocyte density by microscopy at day 14.

| reference | DHA-PPQ | SP-AQ | PY-AS | AS-AQ | AL | Non-ACT-PQ | ACT-PQ | ACT-TQ |
| --- | --- | --- | --- | --- | --- | --- | --- | --- |
| DHA-PPQ |  | 1.68% (-13.29%, 16.66%), p=0.8255 | 4.33% (-11.49%, 20.16%), p=0.5916 | 0.51% (-18.05%, 19.08%), p=0.9568 | 7.58% (-7.53%, 22.68%), p=0.3255 | 7.95% (-0.87%, 16.78%), p=0.0773 | 8.03% (-7.06%, 23.13%), p=0.2970 | 0.84% (-0.94%, 2.61%), p=0.3547 |
| SP-AQ | -1.68% (-16.66%, 13.29%), p=0.8255 |  | 2.65% (-6.07%, 11.36%), p=0.5515 | -1.17% (-14.22%, 11.87%), p=0.8602 | 5.89% (-1.43%, 13.21%), p=0.1146 | 6.27% (-8.06%, 20.60%), p=0.3912 | 6.35% (-0.95%, 13.65%), p=0.0883 | -0.85% (-15.93%, 14.23%), p=0.9124 |
| PY-AS | -4.33% (-20.16%, 11.49%), p=0.5916 | -2.65% (-11.36%, 6.07%), p=0.5515 |  | -3.82% (-15.63%, 7.99%), p=0.5261 | 3.25% (-1.54%, 8.03%), p=0.1842 | 3.62% (-11.95%, 19.19%), p=0.6485 | 3.70% (-1.06%, 8.46%), p=0.1274 | -3.49% (-19.42%, 12.43%), p=0.6672 |
| AS-AQ | -0.51% (-19.08%, 18.05%), p=0.9568 | 1.17% (-11.87%, 14.22%), p=0.8602 | 3.82% (-7.99%, 15.63%), p=0.5261 |  | 7.07% (-3.76%, 17.89%), p=0.2007 | 7.44% (-10.91%, 25.79%), p=0.4267 | 7.52% (-3.29%, 18.33%), p=0.1727 | 0.33% (-18.33%, 18.98%), p=0.9727 |
| AL | -7.58% (-22.68%, 7.53%), p=0.3255 | -5.89% (-13.21%, 1.43%), p=0.1146 | -3.25% (-8.03%, 1.54%), p=0.1842 | -7.07% (-17.89%, 3.76%), p=0.2007 |  | 0.38% (-14.46%, 15.21%), p=0.9604 | 0.46% (-0.10%, 1.01%), p=0.1057 | -6.74% (-21.95%, 8.47%), p=0.3851 |
| Non-ACT-PQ | -7.95% (-16.78%, 0.87%), p=0.0773 | -6.27% (-20.60%, 8.06%), p=0.3912 | -3.62% (-19.19%, 11.95%), p=0.6485 | -7.44% (-25.79%, 10.91%), p=0.4267 | -0.38% (-15.21%, 14.46%), p=0.9604 |  | 0.08% (-14.74%, 14.90%), p=0.9916 | -7.12% (-16.12%, 1.89%), p=0.1213 |
| ACT-PQ | -8.03% (-23.13%, 7.06%), p=0.2970 | -6.35% (-13.65%, 0.95%), p=0.0883 | -3.70% (-8.46%, 1.06%), p=0.1274 | -7.52% (-18.33%, 3.29%), p=0.1727 | -0.46% (-1.01%, 0.10%), p=0.1057 | -0.08% (-14.90%, 14.74%), p=0.9916 |  | -7.19% (-22.39%, 8.01%), p=0.3535 |
| ACT-TQ | -0.84% (-2.61%, 0.94%), p=0.3547 | 0.85% (-14.23%, 15.93%), p=0.9124 | 3.49% (-12.43%, 19.42%), p=0.6672 | -0.33% (-18.98%, 18.33%), p=0.9727 | 6.74% (-8.47%, 21.95%), p=0.3851 | 7.12% (-1.89%, 16.12%), p=0.1213 | 7.19% (-8.01%, 22.39%), p=0.3535 |  |

Results from the network meta-analysis are shown as the absolute difference between treatment groups in the relative reduction from baseline in gametocyte density by microscopy at day 14, with 95% confidence intervals and corresponding p-values. For example, the absolute difference between DHA-PPQ and AL in the relative reduction in microscopical gametocyte density at day 14 is -7.58% (-22.68%, 7.53%), and the difference between these arms (7.58% lower reduction for DHA-PPQ) is not statistically significant (p=0.3255).

## Table H. Treatment comparisons of reduction in gametocyte density by RT-qPCR at day 2.

| reference | DHA-PPQ | SP-AQ | PY-AS | AS-AQ | AL | Non-ACT-PQ | ACT-PQ | ACT-TQ |
| --- | --- | --- | --- | --- | --- | --- | --- | --- |
| DHA-PPQ |  | -6.83% (-84.55%, 70.90%), p=0.8633 | -6.60% (-104.76%, 91.56%), p=0.8952 | -12.02% (-111.32%, 87.27%), p=0.8124 | 12.51% (-56.34%, 81.35%), p=0.7218 | 15.81% (-87.01%, 118.64%), p=0.7631 | 28.76% (-31.10%, 88.62%), p=0.3464 | 7.25% (-84.62%, 99.11%), p=0.8771 |
| SP-AQ | 6.83% (-70.90%, 84.55%), p=0.8633 |  | 0.23% (-104.10%, 104.56%), p=0.9966 | -5.20% (-106.46%, 96.07%), p=0.9199 | 19.34% (-51.06%, 89.73%), p=0.5903 | 22.64% (-83.27%, 128.55%), p=0.6752 | 35.59% (-28.34%, 99.51%), p=0.2752 | 14.07% (-106.26%, 134.41%), p=0.8187 |
| PY-AS | 6.60% (-91.56%, 104.76%), p=0.8952 | -0.23% (-104.56%, 104.10%), p=0.9966 |  | -5.42% (-120.82%, 109.97%), p=0.9266 | 19.11% (-71.64%, 109.85%), p=0.6798 | 22.41% (-112.00%, 156.83%), p=0.7438 | 35.36% (-48.41%, 119.13%), p=0.4080 | 13.85% (-120.60%, 148.29%), p=0.8400 |
| AS-AQ | 12.02% (-87.27%, 111.32%), p=0.8124 | 5.20% (-96.07%, 106.46%), p=0.9199 | 5.42% (-109.97%, 120.82%), p=0.9266 |  | 24.53% (-56.76%, 105.82%), p=0.5542 | 27.84% (-105.99%, 161.67%), p=0.6835 | 40.78% (-38.61%, 120.17%), p=0.3140 | 19.27% (-116.00%, 154.54%), p=0.7801 |
| AL | -12.51% (-81.35%, 56.34%), p=0.7218 | -19.34% (-89.73%, 51.06%), p=0.5903 | -19.11% (-109.85%, 71.64%), p=0.6798 | -24.53% (-105.82%, 56.76%), p=0.5542 |  | 3.31% (-109.43%, 116.04%), p=0.9542 | 16.25% (-18.78%, 51.29%), p=0.3633 | -5.26% (-120.06%, 109.54%), p=0.9284 |
| Non-ACT-PQ | -15.81% (-118.64%, 87.01%), p=0.7631 | -22.64% (-128.55%, 83.27%), p=0.6752 | -22.41% (-156.83%, 112.00%), p=0.7438 | -27.84% (-161.67%, 105.99%), p=0.6835 | -3.31% (-116.04%, 109.43%), p=0.9542 |  | 12.95% (-95.14%, 121.03%), p=0.8144 | -8.57% (-146.45%, 129.32%), p=0.9031 |
| ACT-PQ | -28.76% (-88.62%, 31.10%), p=0.3464 | -35.59% (-99.51%, 28.34%), p=0.2752 | -35.36% (-119.13%, 48.41%), p=0.4080 | -40.78% (-120.17%, 38.61%), p=0.3140 | -16.25% (-51.29%, 18.78%), p=0.3633 | -12.95% (-121.03%, 95.14%), p=0.8144 |  | -21.51% (-131.16%, 88.14%), p=0.7006 |
| ACT-TQ | -7.25% (-99.11%, 84.62%), p=0.8771 | -14.07% (-134.41%, 106.26%), p=0.8187 | -13.85% (-148.29%, 120.60%), p=0.8400 | -19.27% (-154.54%, 116.00%), p=0.7801 | 5.26% (-109.54%, 120.06%), p=0.9284 | 8.57% (-129.32%, 146.45%), p=0.9031 | 21.51% (-88.14%, 131.16%), p=0.7006 |  |

Results from the network meta-analysis are shown as the absolute difference between treatment groups in the relative reduction from baseline in gametocyte density by RT-qPCR at day 2, with 95% confidence intervals and corresponding p-values. For example, the absolute difference between DHA-PPQ and AL in the relative reduction in molecular gametocyte density at day 2 is -12.51% (-81.35%, 56.34%), and the difference between these arms (12.51% lower reduction for DHA-PPQ) is not statistically significant (p=0.7218).

## Table I. Treatment comparisons of reduction in gametocyte density by RT-qPCR at day 7.

| reference | DHA-PPQ | SP-AQ | PY-AS | AS-AQ | AL | Non-ACT-PQ | ACT-PQ | ACT-TQ |
| --- | --- | --- | --- | --- | --- | --- | --- | --- |
| DHA-PPQ |  | 0.81% (-44.17%, 45.78%), p=0.9720 | -1.48% (-54.54%, 51.58%), p=0.9564 | 9.23% (-38.68%, 57.15%), p=0.7056 | 18.50% (-17.49%, 54.50%), p=0.3137 | 51.41% (7.71%, 95.12%), p=0.0211 | 43.39% (10.60%, 76.18%), p=0.0095 | 26.34% (-19.12%, 71.80%), p=0.2561 |
| SP-AQ | -0.81% (-45.78%, 44.17%), p=0.9720 |  | -2.29% (-57.97%, 53.39%), p=0.9359 | 8.43% (-42.37%, 59.23%), p=0.7450 | 17.70% (-22.06%, 57.45%), p=0.3830 | 50.61% (1.61%, 99.60%), p=0.0429 | 42.58% (5.71%, 79.46%), p=0.0236 | 25.53% (-38.41%, 89.48%), p=0.4338 |
| PY-AS | 1.48% (-51.58%, 54.54%), p=0.9564 | 2.29% (-53.39%, 57.97%), p=0.9359 |  | 10.71% (-43.70%, 65.13%), p=0.6996 | 19.98% (-24.30%, 64.26%), p=0.3765 | 52.89% (-10.89%, 116.67%), p=0.1041 | 44.87% (3.15%, 86.59%), p=0.0350 | 27.82% (-42.05%, 97.69%), p=0.4352 |
| AS-AQ | -9.23% (-57.15%, 38.68%), p=0.7056 | -8.43% (-59.23%, 42.37%), p=0.7450 | -10.71% (-65.13%, 43.70%), p=0.6996 |  | 9.27% (-28.70%, 47.23%), p=0.6324 | 42.18% (-17.39%, 101.74%), p=0.1652 | 34.16% (-0.79%, 69.10%), p=0.0554 | 17.10% (-48.94%, 83.15%), p=0.6118 |
| AL | -18.50% (-54.50%, 17.49%), p=0.3137 | -17.70% (-57.45%, 22.06%), p=0.3830 | -19.98% (-64.26%, 24.30%), p=0.3765 | -9.27% (-47.23%, 28.70%), p=0.6324 |  | 32.91% (-17.57%, 83.38%), p=0.2013 | 24.89% (10.04%, 39.74%), p=0.0010 | 7.84% (-50.14%, 65.82%), p=0.7911 |
| Non-ACT-PQ | -51.41% (-95.12%, -7.71%), p=0.0211 | -50.61% (-99.60%, -1.61%), p=0.0429 | -52.89% (-116.67%, 10.89%), p=0.1041 | -42.18% (-101.74%, 17.39%), p=0.1652 | -32.91% (-83.38%, 17.57%), p=0.2013 |  | -8.02% (-56.26%, 40.22%), p=0.7445 | -25.07% (-88.13%, 37.99%), p=0.4358 |
| ACT-PQ | -43.39% (-76.18%, -10.60%), p=0.0095 | -42.58% (-79.46%, -5.71%), p=0.0236 | -44.87% (-86.59%, -3.15%), p=0.0350 | -34.16% (-69.10%, 0.79%), p=0.0554 | -24.89% (-39.74%, -10.04%), p=0.0010 | 8.02% (-40.22%, 56.26%), p=0.7445 |  | -17.05% (-73.10%, 39.00%), p=0.5510 |
| ACT-TQ | -26.34% (-71.80%, 19.12%), p=0.2561 | -25.53% (-89.48%, 38.41%), p=0.4338 | -27.82% (-97.69%, 42.05%), p=0.4352 | -17.10% (-83.15%, 48.94%), p=0.6118 | -7.84% (-65.82%, 50.14%), p=0.7911 | 25.07% (-37.99%, 88.13%), p=0.4358 | 17.05% (-39.00%, 73.10%), p=0.5510 |  |

Results from the network meta-analysis are shown as the absolute difference between treatment groups in the relative reduction from baseline in gametocyte density by RT-qPCR at day 7, with 95% confidence intervals and corresponding p-values. For example, the absolute difference between DHA-PPQ and AL in the relative reduction in molecular gametocyte density at day 7 is -18.50% (-54.50%, 17.49%), and the difference between these arms (18.50% lower reduction for DHA-PPQ) is not statistically significant (p=0.3137).

## Table J. Treatment comparisons of reduction in gametocyte density by RT-qPCR at day 14.

| reference | DHA-PPQ | SP-AQ | PY-AS | AS-AQ | AL | Non-ACT-PQ | ACT-PQ | ACT-TQ |
| --- | --- | --- | --- | --- | --- | --- | --- | --- |
| DHA-PPQ |  | -4.74% (-31.87%, 22.39%), p=0.7322 | 1.92% (-27.51%, 31.35%), p=0.8981 | 8.72% (-17.41%, 34.85%), p=0.5132 | 19.35% (-0.36%, 39.05%), p=0.0543 | 23.67% (2.44%, 44.90%), p=0.0289 | 25.84% (6.52%, 45.16%), p=0.0087 | 25.26% (-1.21%, 51.72%), p=0.0614 |
| SP-AQ | 4.74% (-22.39%, 31.87%), p=0.7322 |  | 6.66% (-26.63%, 39.95%), p=0.6951 | 13.46% (-16.96%, 43.88%), p=0.3860 | 24.08% (-1.03%, 49.20%), p=0.0602 | 28.40% (1.83%, 54.97%), p=0.0361 | 30.58% (5.77%, 55.39%), p=0.0157 | 29.99% (-7.91%, 67.90%), p=0.1209 |
| PY-AS | -1.92% (-31.35%, 27.51%), p=0.8981 | -6.66% (-39.95%, 26.63%), p=0.6951 |  | 6.80% (-21.53%, 35.13%), p=0.6382 | 17.43% (-5.11%, 39.97%), p=0.1297 | 21.75% (-12.25%, 55.74%), p=0.2099 | 23.92% (1.72%, 46.12%), p=0.0347 | 23.34% (-16.24%, 62.91%), p=0.2479 |
| AS-AQ | -8.72% (-34.85%, 17.41%), p=0.5132 | -13.46% (-43.88%, 16.96%), p=0.3860 | -6.80% (-35.13%, 21.53%), p=0.6382 |  | 10.63% (-7.40%, 28.65%), p=0.2478 | 14.95% (-16.23%, 46.13%), p=0.3474 | 17.12% (-0.48%, 34.72%), p=0.0566 | 16.54% (-20.66%, 53.73%), p=0.3835 |
| AL | -19.35% (-39.05%, 0.36%), p=0.0543 | -24.08% (-49.20%, 1.03%), p=0.0602 | -17.43% (-39.97%, 5.11%), p=0.1297 | -10.63% (-28.65%, 7.40%), p=0.2478 |  | 4.32% (-21.71%, 30.35%), p=0.7451 | 6.49% (2.60%, 10.38%), p=0.0011 | 5.91% (-27.09%, 38.91%), p=0.7256 |
| Non-ACT-PQ | -23.67% (-44.90%, -2.44%), p=0.0289 | -28.40% (-54.97%, -1.83%), p=0.0361 | -21.75% (-55.74%, 12.25%), p=0.2099 | -14.95% (-46.13%, 16.23%), p=0.3474 | -4.32% (-30.35%, 21.71%), p=0.7451 |  | 2.17% (-23.57%, 27.91%), p=0.8686 | 1.59% (-32.34%, 35.52%), p=0.9268 |
| ACT-PQ | -25.84% (-45.16%, -6.52%), p=0.0087 | -30.58% (-55.39%, -5.77%), p=0.0157 | -23.92% (-46.12%, -1.72%), p=0.0347 | -17.12% (-34.72%, 0.48%), p=0.0566 | -6.49% (-10.38%, -2.60%), p=0.0011 | -2.17% (-27.91%, 23.57%), p=0.8686 |  | -0.58% (-33.35%, 32.18%), p=0.9722 |
| ACT-TQ | -25.26% (-51.72%, 1.21%), p=0.0614 | -29.99% (-67.90%, 7.91%), p=0.1209 | -23.34% (-62.91%, 16.24%), p=0.2479 | -16.54% (-53.73%, 20.66%), p=0.3835 | -5.91% (-38.91%, 27.09%), p=0.7256 | -1.59% (-35.52%, 32.34%), p=0.9268 | 0.58% (-32.18%, 33.35%), p=0.9722 |  |

Results from the network meta-analysis are shown as the absolute difference between treatment groups in the relative reduction from baseline in gametocyte density by RT-qPCR at day 14, with 95% confidence intervals and corresponding p-values. For example, the absolute difference between DHA-PPQ and AL in the relative reduction in molecular gametocyte density at day 14 is -19.35% (-39.05%, 0.36%), and the difference between these arms (19.35% lower reduction for DHA-PPQ) is nearly statistically significant (p=0.0543).

## Fig G. Forest plots of treatment comparisons of reduction in gametocyte density by microscopy at days 2, 7, and 14. All comparisons are with ACT-PQ.


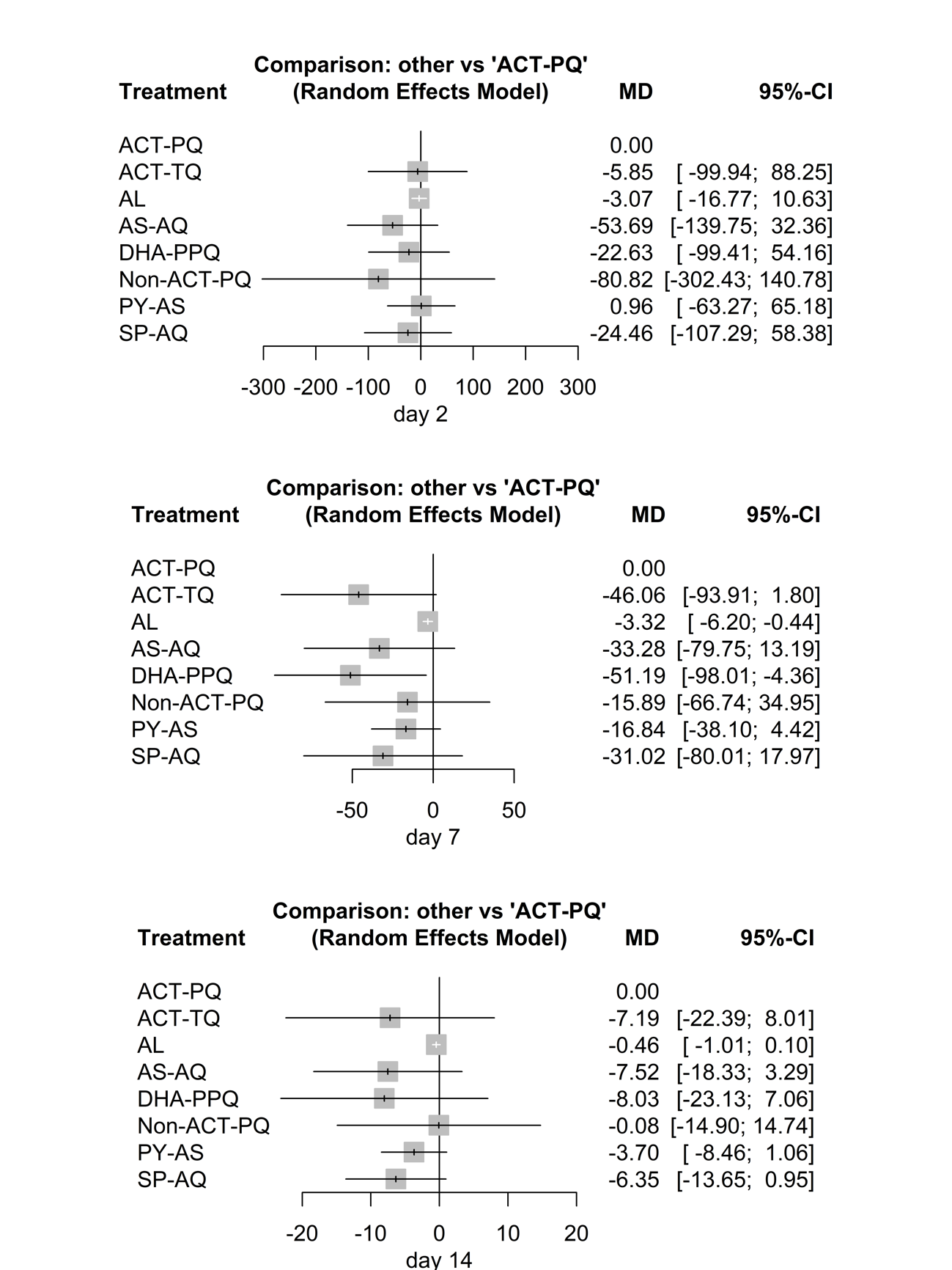


Results from the network meta-analysis are shown as mean differences (MD) in relative reductions from baseline in gametocyte density by microscopy, with 95% confidence intervals. Each treatment is compared to ACT-PQ, the reference treatment. Negative values indicate a smaller reduction than ACT-PQ, while positive values indicate a larger reduction. Point estimates are plotted as squares proportional to study weight, and horizontal lines denote confidence intervals.

Fig H. Forest plots of treatment comparisons of reduction in gametocyte density by RT-qPCR at days 2, 7, and 14. All comparisons are with ACT-PQ.

Results from the network meta-analysis are shown as mean differences (MD) in relative reductions from baseline in gametocyte density by RT-qPCR, with 95% confidence intervals. Each treatment is compared to ACT-PQ, the reference treatment. Negative values indicate a smaller reduction than ACT-PQ, while positive values indicate a larger reduction. Point estimates are plotted as squares proportional to study weight, and horizontal lines denote confidence intervals.


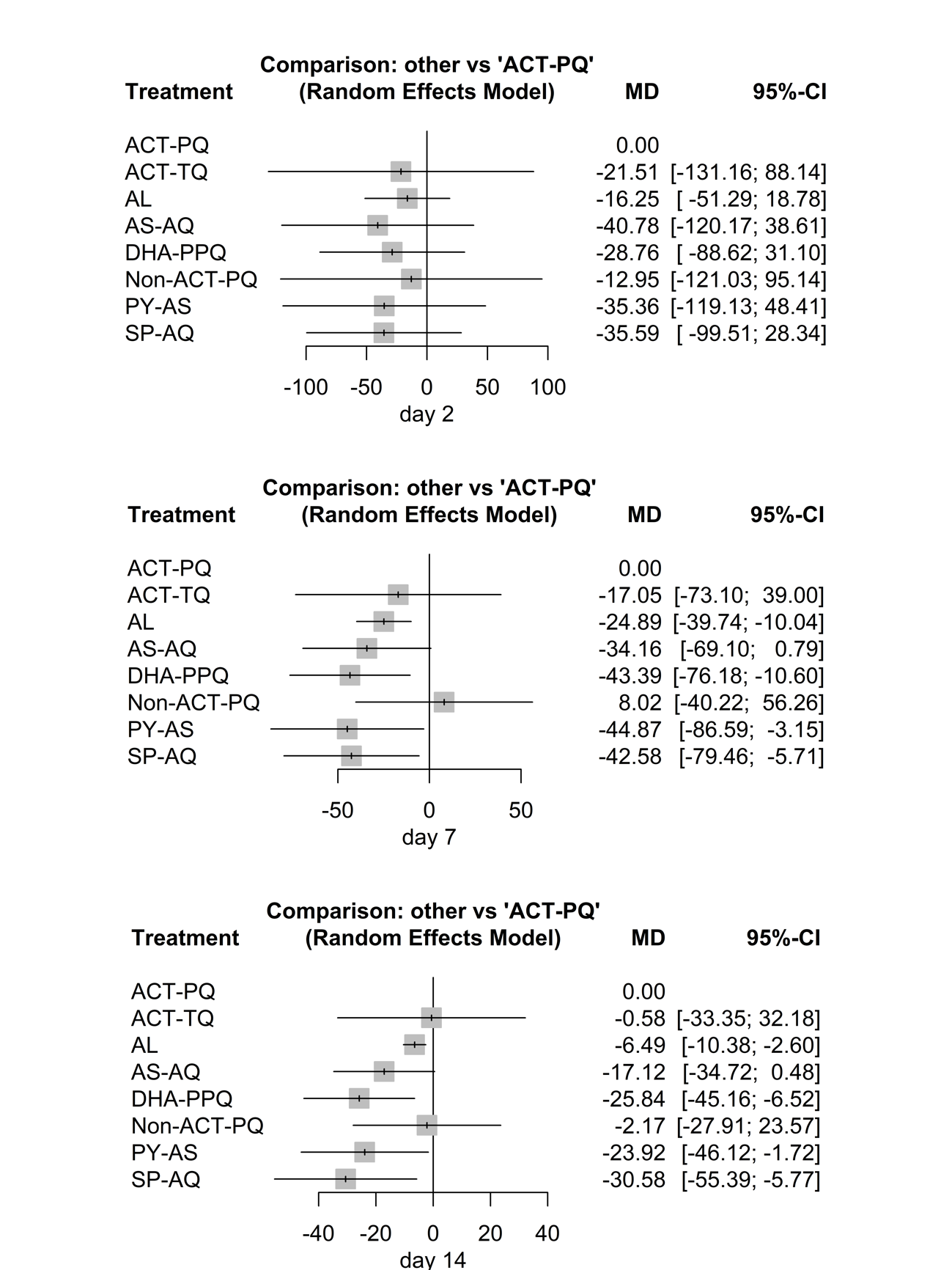


## Table K. Relative reduction in proportion infected mosquitoes

| **Treatment** | **Day 2** | **Day 7** | **Day 14** |
| --- | --- | --- | --- |
| **DHA-PPQ** | 15.64% (5.15%, 24.97%), p=0.0045 | 43.25% (35.33%, 50.20%), p<0.0001 | 67.65% (60.67%, 73.40%), p<0.0001 |
| **SP-AQ** | 1.01% (-13.77%, 13.88%), p=0.8858 | 9.89% (-3.76%, 21.73%), p=0.1479 | 81.07% (73.63%, 86.41%), p<0.0001 |
| **PY-AS** | -4.46% (-29.01%, 15.42%), p=0.6855 | -2.84% (-26.20%, 16.20%), p=0.7890 | 70.80% (60.37%, 78.49%), p<0.0001 |
| **AS-AQ** | -7.40% (-34.62%, 14.32%), p=0.5356 | 62.28% (48.02%, 72.62%), p<0.0001 | 82.25% (71.61%, 88.90%), p<0.0001 |
| **AL** | 97.15% (94.94%, 98.40%), p<0.0001 | 99.76% (98.27%, 99.97%), p<0.0001 | 99.60% (97.19%, 99.94%), p<0.0001 |
| **Non-ACT-PQ** | 84.44% (79.80%, 88.02%), p<0.0001 | 99.88% (98.02%, 99.99%), p<0.0001 | No Data |
| **ACT-PQ** | 98.93% (98.20%, 99.37%), p<0.0001 | 99.69% (99.17%, 99.88%), p<0.0001 | 99.22% (96.89%, 99.81%), p<0.0001 |
| **ACT-TQ** | -2.19% (-18.31%, 11.73%), p=0.7719 | 99.42% (97.69%, 99.86%), p<0.0001 | 99.80% (96.83%, 99.99%), p<0.0001 |

Relative reduction compared to baseline in proportion infected mosquitoes, at three time points (Day 2, Day 7, Day 14), with 95% confidence intervals and corresponding p-values.

## Fig I. Forest plots of treatment comparisons of reduction in proportion infected mosquitoes at days 2, 7, and 14. All comparisons are with ACT-PQ.


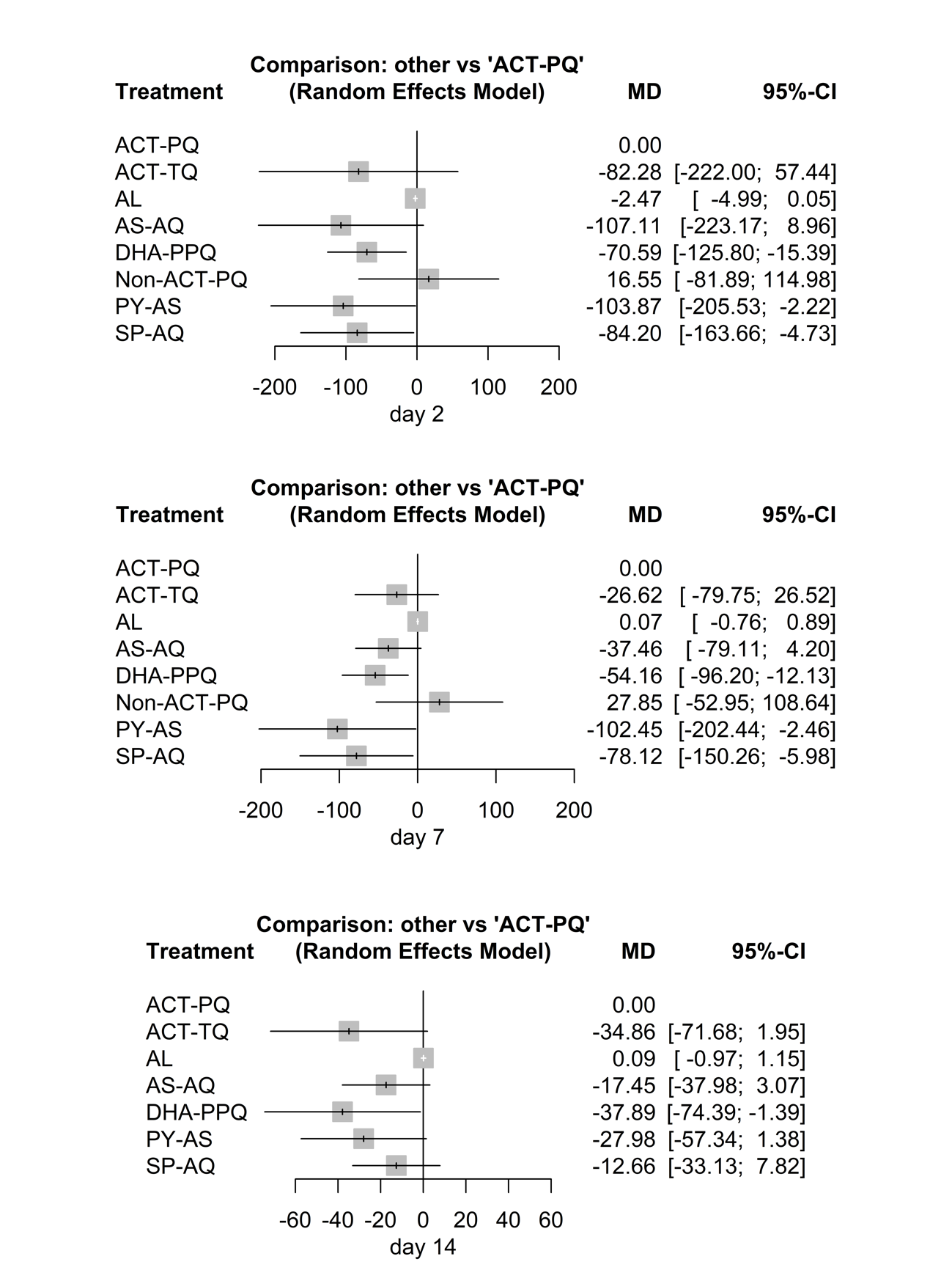


Results from the network meta-analysis are shown as mean differences (MD) in relative reductions from baseline in proportion infected mosquitoes, with 95% confidence intervals. Each treatment is compared to ACT-PQ, the reference treatment. Negative values indicate a smaller reduction than ACT-PQ, while positive values indicate a larger reduction. Point estimates are plotted as squares proportional to study weight, and horizontal lines denote confidence intervals.

## Fig J. Relative reduction in proportion infected mosquitoes per study arm (ungrouped)


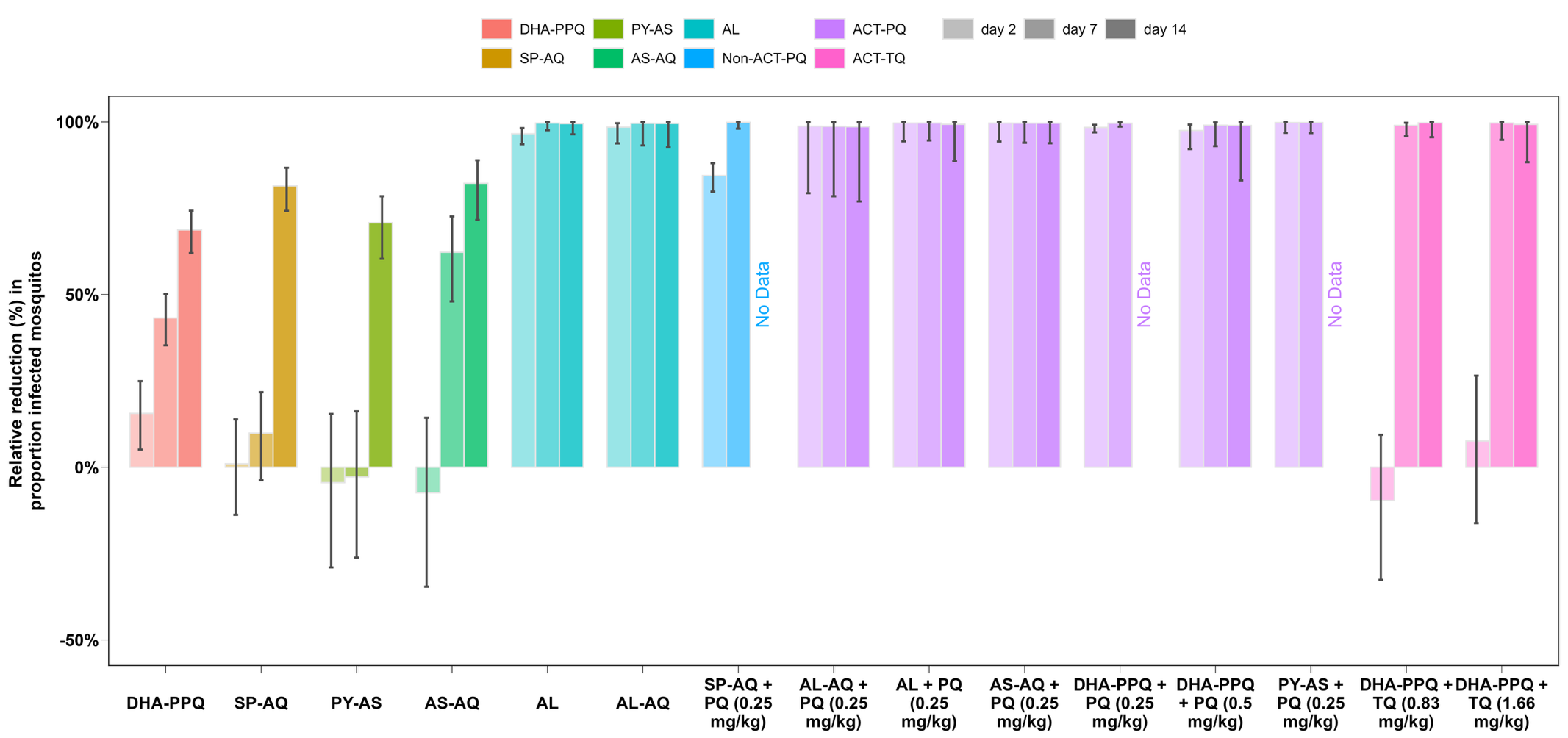


## Fig K. Relative reduction in proportion infected mosquitoes comparing the same study arms across different studies

Bar charts illustrating the relative reduction compared to baseline in the proportion infected mosquitoes for each study arm (ungrouped), over three time points (Day 2, Day 7, Day 14). Vertical bars depict the 95% confidence intervals for these estimates.


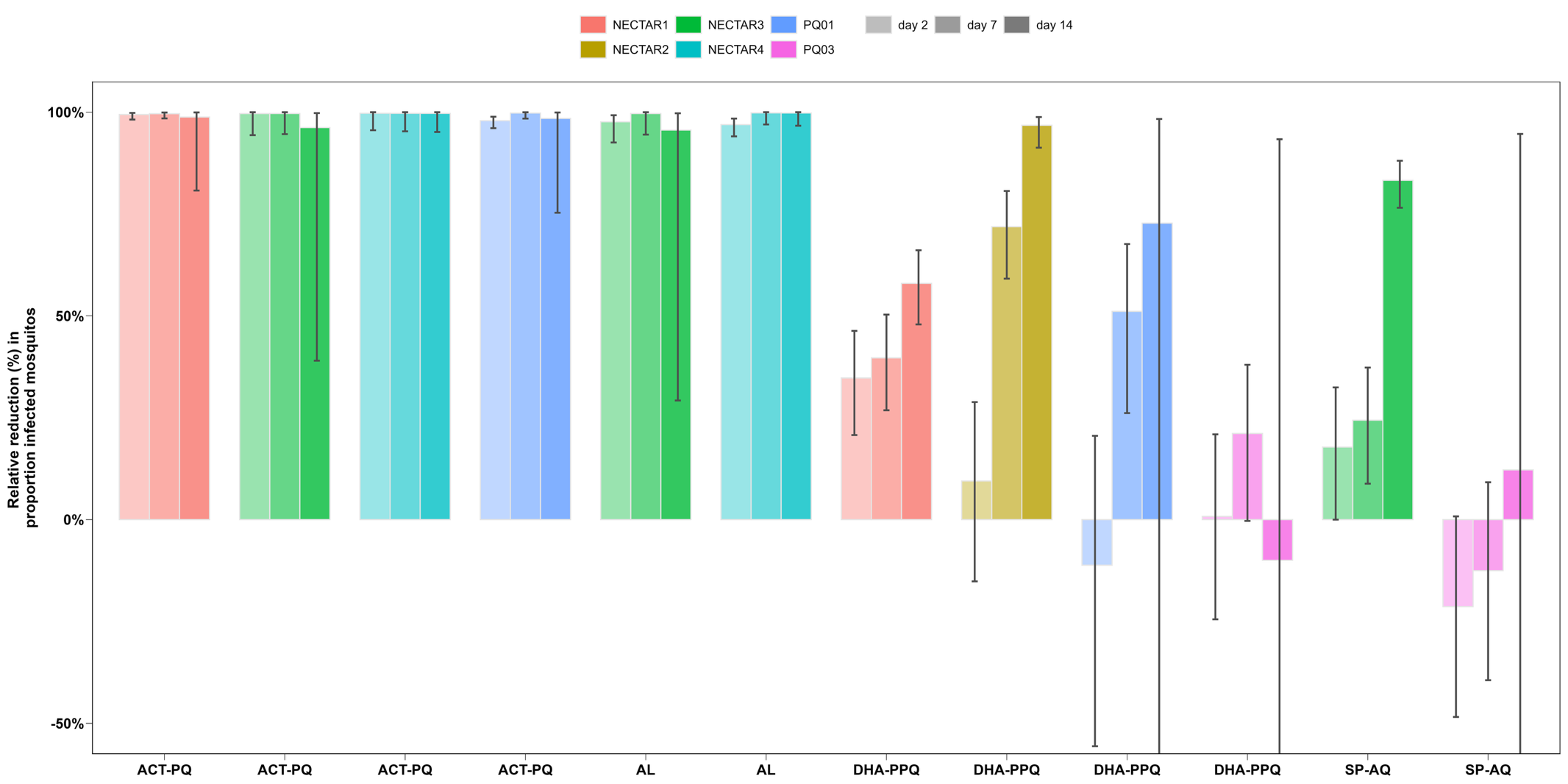


Bar charts illustrating the relative reduction compared to baseline in the proportion infected mosquitoes for each study arm over three time points (Day 2, Day 7, Day 14) and for each study that it was evaluated in. Vertical bars depict the 95% confidence intervals for these estimates.

## Fig L. Relative reduction in the probability of infecting at least 1 mosquito

**
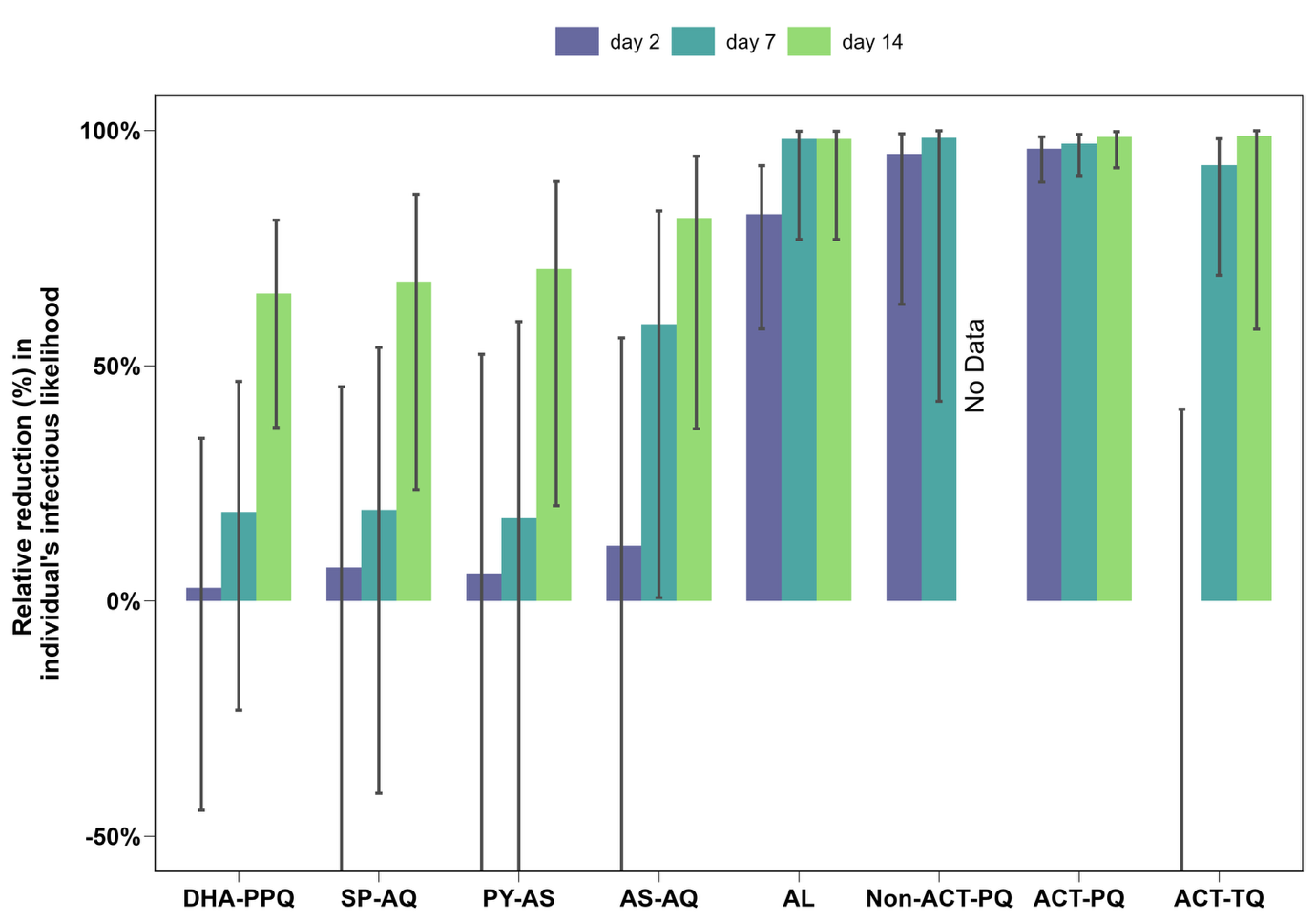
**

Relative reduction compared to baseline in the probability of infecting at least 1 mosquito, at three time points (Day 2, Day 7, Day 14), with 95% confidence intervals.

## Table L. Relative reduction in the probability of infecting at least 1 mosquito

| **Treatment** | **Day 2** | **Day 7** | **Day 14** |
| --- | --- | --- | --- |
| **DHA-PPQ** | 2.77% (-44.48%, 34.57%), p=0.8895 | 18.92% (-23.24%, 46.66%), p=0.3262 | 65.34% (36.89%, 80.97%), p=0.0005 |
| **SP-AQ** | 7.14% (-58.36%, 45.55%), p=0.7855 | 19.41% (-40.86%, 53.90%), p=0.4487 | 67.86% (23.72%, 86.46%), p=0.0101 |
| **PY-AS** | 5.88% (-86.28%, 52.45%), p=0.8618 | 17.65% (-67.07%, 59.41%), p=0.5906 | 70.59% (20.28%, 89.15%), p=0.0162 |
| **AS-AQ** | 11.76% (-76.68%, 55.93%), p=0.7238 | 58.82% (0.71%, 82.92%), p=0.0482 | 81.42% (36.61%, 94.56%), p=0.0072 |
| **AL** | 82.27% (57.84%, 92.54%), p<0.0001 | 98.20% (76.85%, 99.86%), p=0.0020 | 98.20% (76.85%, 99.86%), p=0.0020 |
| **Non-ACT-PQ** | 95.02% (63.07%, 99.33%), p=0.0033 | 98.43% (42.45%, 99.96%), p=0.0238 | No Data |
| **ACT-PQ** | 96.17% (89.02%, 98.66%), p<0.0001 | 97.21% (90.44%, 99.19%), p<0.0001 | 98.69% (92.09%, 99.78%), p<0.0001 |
| **ACT-TQ** | 0.00% (-68.85%, 40.78%), p>0.9999 | 92.67% (69.25%, 98.25%), p=0.0004 | 98.84% (57.78%, 99.97%), p=0.0151 |

Relative reduction compared to baseline in the probability of infecting at least 1 mosquito, at three time points (Day 2, Day 7, Day 14), with 95% confidence intervals and corresponding p-values.

## Fig M. Relative reduction in the probability of infecting at least 1 mosquito per study arm (ungrouped)

**
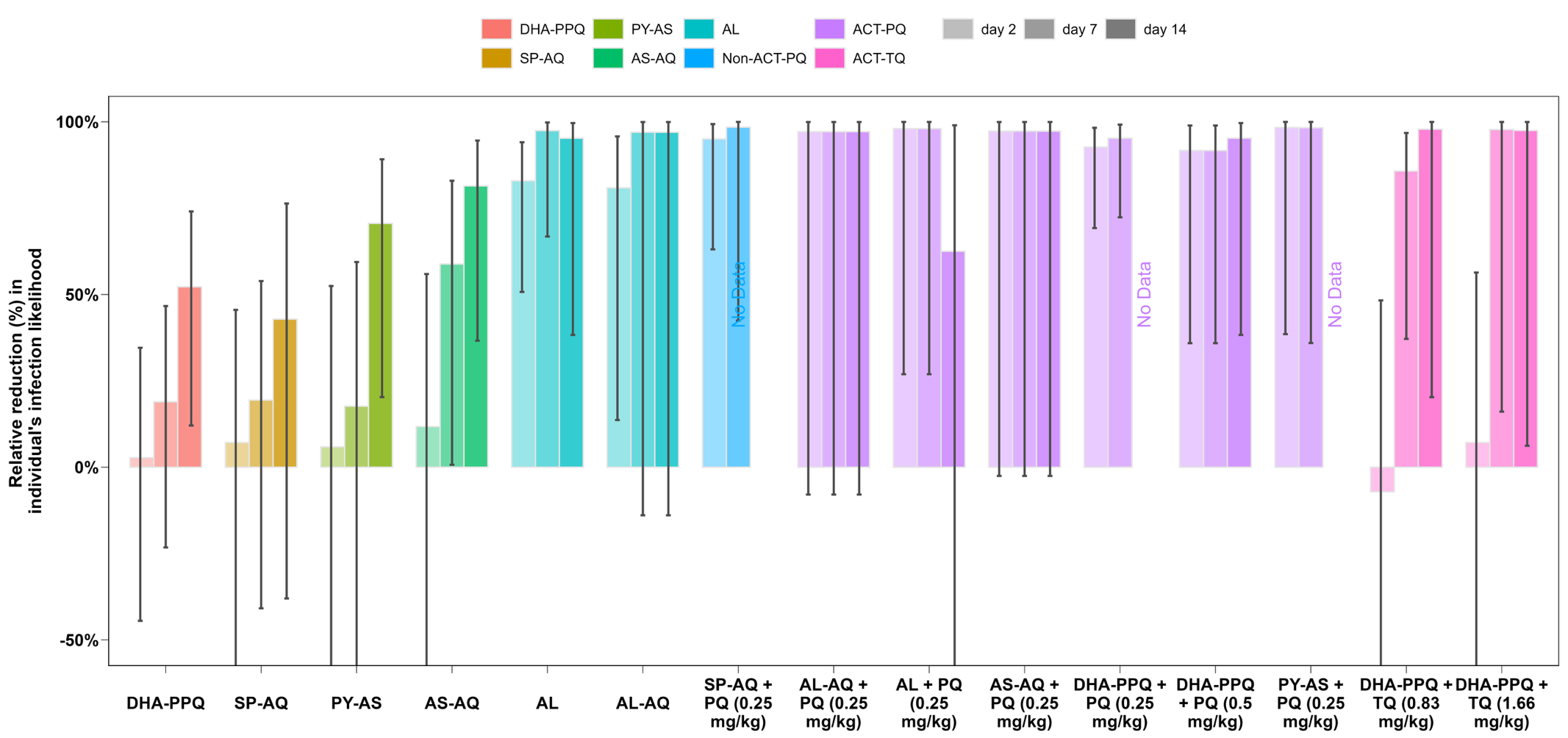
**

Bar charts illustrating the relative reduction compared to baseline in the probability of infecting at least 1 mosquito for each study arm (ungrouped), over three time points (Day 2, Day 7, Day 14). Vertical bars depict the 95% confidence intervals for these estimates.

## Fig N. Forest plots of treatment comparisons of probability of infecting at least 1 mosquito at days 2, 7, and 14. All comparisons are with ACT-PQ.

Legend

**
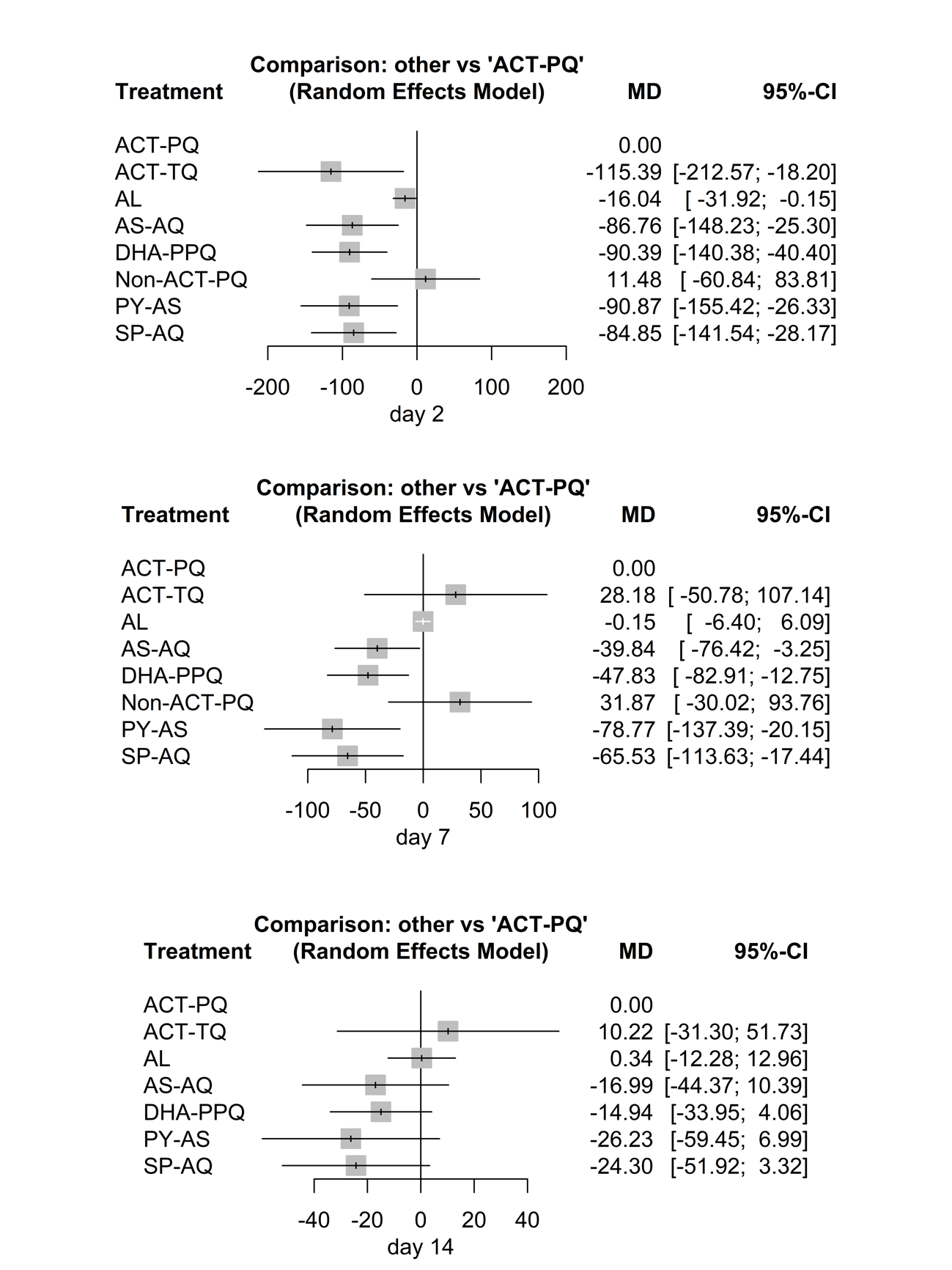
**

Results from the network meta-analysis are shown as mean differences (MD) in relative reductions from baseline in infectiousness, with 95% confidence intervals. Each treatment is compared to ACT-PQ, the reference treatment. Negative values indicate a smaller reduction than ACT-PQ, while positive values indicate a larger reduction. Point estimates are plotted as squares proportional to study weight, and horizontal lines denote confidence intervals.

## Fig O. Relative reduction in oocyst density

**
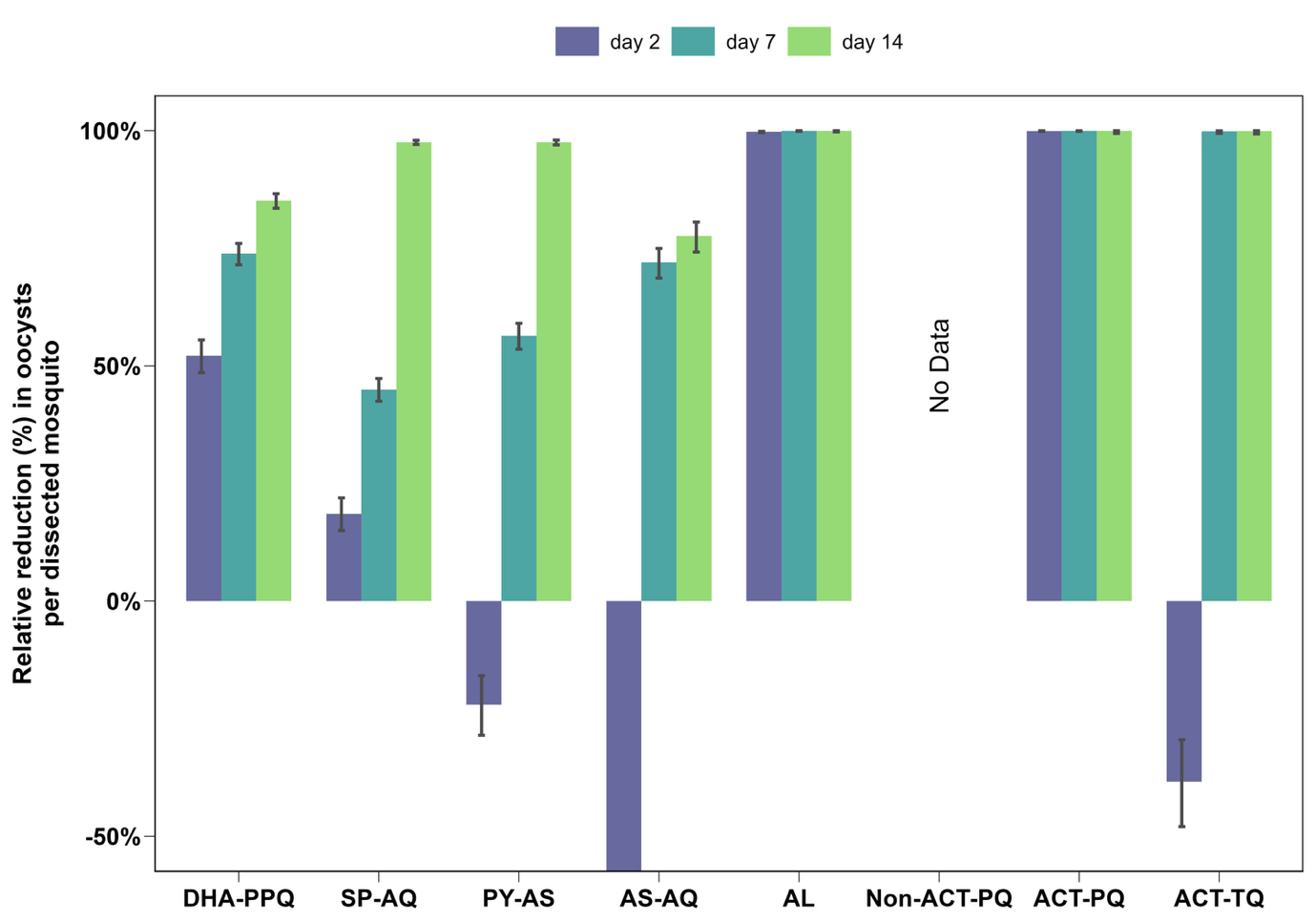
**

Relative reduction compared to baseline in oocyst density, at three time points (Day 2, Day 7, Day 14), with 95% confidence intervals.

## Table M. Relative reduction in oocyst density

| **Treatment** | **Day 2** | **Day 7** | **Day 14** |
| --- | --- | --- | --- |
| **DHA-PPQ** | 52.16% (48.55%, 55.51%), p<0.0001 | 73.86% (71.50%, 76.03%), p<0.0001 | 85.14% (83.51%, 86.62%), p<0.0001 |
| **SP-AQ** | 18.54% (15.00%, 21.93%), p<0.0001 | 44.95% (42.48%, 47.31%), p<0.0001 | 97.55% (97.07%, 97.95%), p<0.0001 |
| **PY-AS** | -22.03% (-28.53%, -15.86%), p<0.0001 | 56.38% (53.54%, 59.04%), p<0.0001 | 97.54% (96.97%, 98.01%), p<0.0001 |
| **AS-AQ** | -96.49% (-108.67%, -85.02%), p<0.0001 | 72.00% (68.66%, 74.98%), p<0.0001 | 77.62% (74.19%, 80.58%), p<0.0001 |
| **AL** | 99.76% (99.57%, 99.86%), p<0.0001 | 99.97% (99.85%, 100.00%), p<0.0001 | 99.98% (99.74%, 100.00%), p<0.0001 |
| **ACT-PQ** | 99.94% (99.85%, 99.98%), p<0.0001 | 99.96% (99.87%, 99.99%), p<0.0001 | 99.95% (99.43%, 100.00%), p<0.0001 |
| **ACT-TQ** | -38.42% (-47.95%, -29.50%), p<0.0001 | 99.86% (99.44%, 99.96%), p<0.0001 | 99.94% (99.32%, 99.99%), p<0.0001 |

Relative reduction compared to baseline in oocyst density, at three time points (Day 2, Day 7, Day 14), with 95% confidence intervals and corresponding p-values.

## Fig P. Relative reduction in oocyst density per study arm (ungrouped)

**
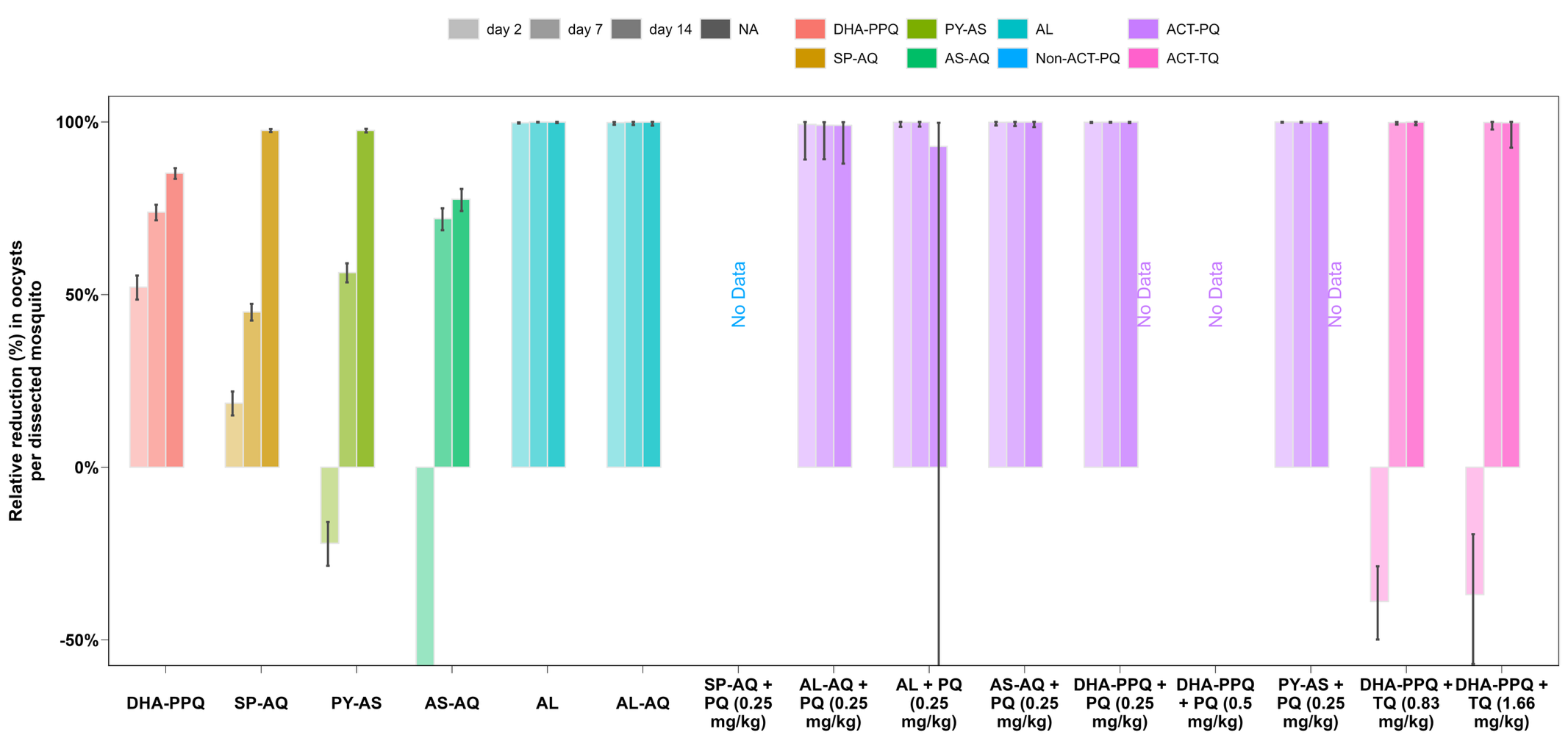
**

Bar charts illustrating the relative reduction compared to baseline in oocyst density for each study arm (ungrouped), over three time points (Day 2, Day 7, Day 14). Vertical bars depict the 95% confidence intervals for these estimates.

## Fig Q. Forest plots of treatment comparisons of reduction in oocyst density at days 2, 7, and 14. All comparisons are with ACT-PQ.

**
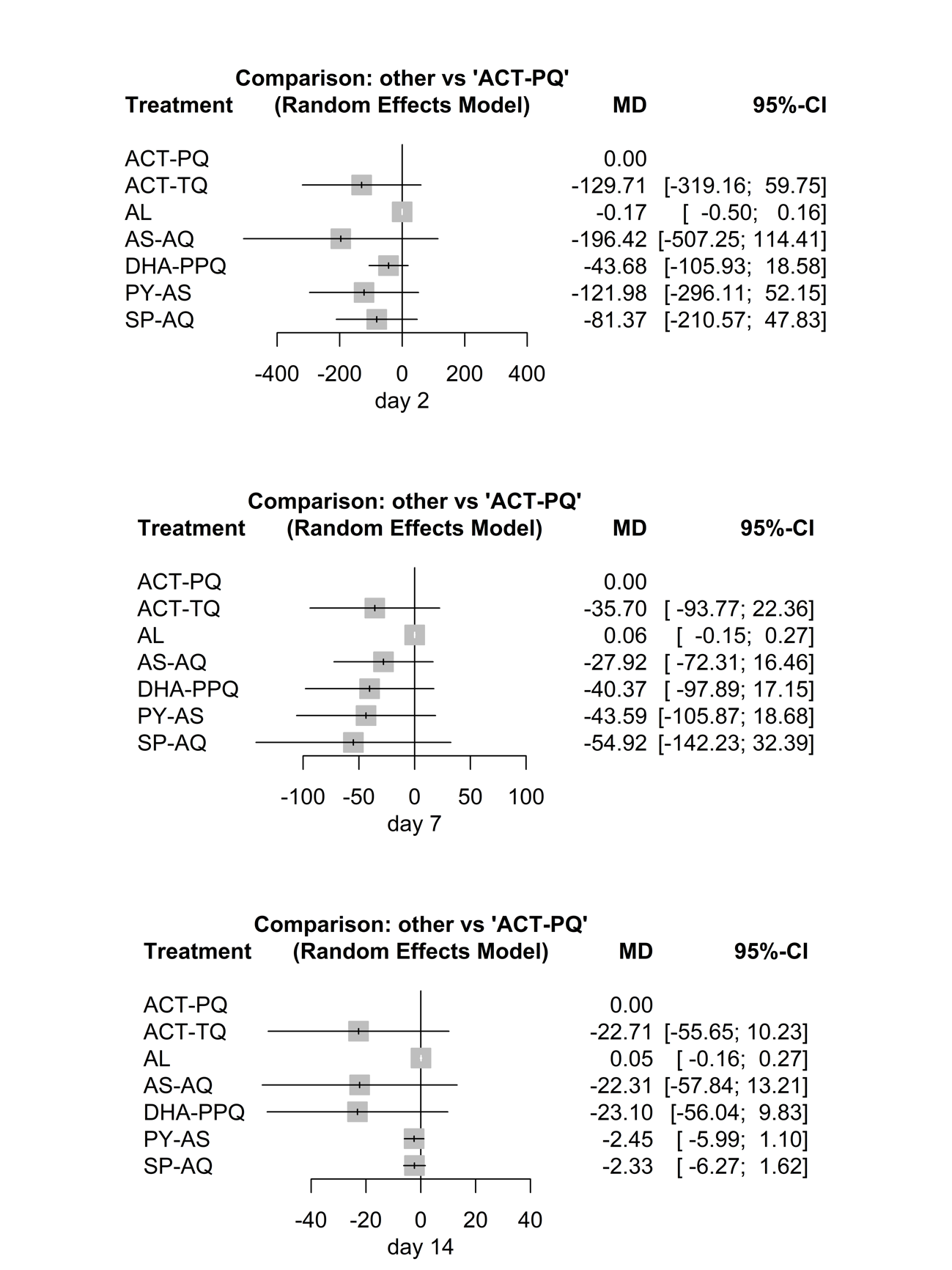
**

Results from the network meta-analysis are shown as mean differences (MD) in relative reductions from baseline in oocyst density, with 95% confidence intervals. Each treatment is compared to ACT-PQ, the reference treatment. Negative values indicate a smaller reduction than ACT-PQ, while positive values indicate a larger reduction. Point estimates are plotted as squares proportional to study weight, and horizontal lines denote confidence intervals.

## Table N. Kaplan–Meier survival data for time to clearance of infectivity and gametocytes, by treatment arm

| time | n.risk | n.event | n.censor | surv | upper | lower | arm | type |
| --- | --- | --- | --- | --- | --- | --- | --- | --- |
| 1 | 84 | 0 | 1 | 1 | 1 | 1 | DHA-PPQ | gametocytes (pcr) |
| 2 | 83 | 0 | 2 | 1 | 1 | 1 | DHA-PPQ | gametocytes (pcr) |
| 10 | 81 | 1 | 1 | 0.987654 | 1 | 0.963897 | DHA-PPQ | gametocytes (pcr) |
| 14 | 79 | 1 | 2 | 0.975152 | 1 | 0.941731 | DHA-PPQ | gametocytes (pcr) |
| 21 | 76 | 2 | 0 | 0.94949 | 0.998987 | 0.902446 | DHA-PPQ | gametocytes (pcr) |
| 28 | 74 | 8 | 31 | 0.846843 | 0.930498 | 0.770708 | DHA-PPQ | gametocytes (pcr) |
| 35 | 35 | 4 | 0 | 0.750061 | 0.872998 | 0.644436 | DHA-PPQ | gametocytes (pcr) |
| 42 | 31 | 19 | 6 | 0.290346 | 0.463731 | 0.181788 | DHA-PPQ | gametocytes (pcr) |
| 49 | 6 | 4 | 2 | 0.096782 | 0.329341 | 0.028441 | DHA-PPQ | gametocytes (pcr) |
| 2 | 40 | 0 | 1 | 1 | 1 | 1 | SP-AQ | gametocytes (pcr) |
| 28 | 39 | 0 | 25 | 1 | 1 | 1 | SP-AQ | gametocytes (pcr) |
| 42 | 14 | 7 | 7 | 0.5 | 0.844235 | 0.296126 | SP-AQ | gametocytes (pcr) |
| 10 | 25 | 1 | 0 | 0.96 | 1 | 0.886178 | PY-AS | gametocytes (pcr) |
| 14 | 24 | 0 | 3 | 0.96 | 1 | 0.886178 | PY-AS | gametocytes (pcr) |
| 28 | 21 | 1 | 1 | 0.914286 | 1 | 0.807101 | PY-AS | gametocytes (pcr) |
| 35 | 19 | 6 | 0 | 0.625564 | 0.870091 | 0.449758 | PY-AS | gametocytes (pcr) |
| 42 | 13 | 6 | 0 | 0.336842 | 0.614864 | 0.184533 | PY-AS | gametocytes (pcr) |
| 49 | 7 | 5 | 2 | 0.096241 | 0.359136 | 0.02579 | PY-AS | gametocytes (pcr) |
| 7 | 22 | 0 | 1 | 1 | 1 | 1 | AS-AQ | gametocytes (pcr) |
| 14 | 21 | 1 | 0 | 0.952381 | 1 | 0.865518 | AS-AQ | gametocytes (pcr) |
| 21 | 20 | 2 | 0 | 0.857143 | 1 | 0.719817 | AS-AQ | gametocytes (pcr) |
| 28 | 18 | 1 | 17 | 0.809524 | 0.996163 | 0.657853 | AS-AQ | gametocytes (pcr) |
| 2 | 67 | 0 | 1 | 1 | 1 | 1 | AL | gametocytes (pcr) |
| 5 | 66 | 1 | 0 | 0.984848 | 1 | 0.955814 | AL | gametocytes (pcr) |
| 7 | 65 | 1 | 0 | 0.969697 | 1 | 0.929211 | AL | gametocytes (pcr) |
| 14 | 64 | 4 | 0 | 0.909091 | 0.981161 | 0.842315 | AL | gametocytes (pcr) |
| 21 | 60 | 9 | 0 | 0.772727 | 0.880742 | 0.677959 | AL | gametocytes (pcr) |
| 28 | 51 | 15 | 36 | 0.545455 | 0.679838 | 0.437635 | AL | gametocytes (pcr) |
| 2 | 21 | 0 | 1 | 1 | 1 | 1 | Non-ACT-PQ | gametocytes (pcr) |
| 7 | 20 | 0 | 1 | 1 | 1 | 1 | Non-ACT-PQ | gametocytes (pcr) |
| 14 | 19 | 11 | 0 | 0.421053 | 0.713381 | 0.248514 | Non-ACT-PQ | gametocytes (pcr) |
| 28 | 8 | 4 | 0 | 0.210526 | 0.502878 | 0.088135 | Non-ACT-PQ | gametocytes (pcr) |
| 42 | 4 | 4 | 0 | 0 |  |  | Non-ACT-PQ | gametocytes (pcr) |
| 1 | 153 | 0 | 1 | 1 | 1 | 1 | ACT-PQ | gametocytes (pcr) |
| 2 | 152 | 0 | 4 | 1 | 1 | 1 | ACT-PQ | gametocytes (pcr) |
| 3 | 148 | 0 | 11 | 1 | 1 | 1 | ACT-PQ | gametocytes (pcr) |
| 5 | 137 | 3 | 0 | 0.978102 | 1 | 0.9539 | ACT-PQ | gametocytes (pcr) |
| 7 | 134 | 50 | 6 | 0.613139 | 0.700365 | 0.536776 | ACT-PQ | gametocytes (pcr) |
| 14 | 78 | 45 | 4 | 0.259405 | 0.347123 | 0.193853 | ACT-PQ | gametocytes (pcr) |
| 21 | 29 | 15 | 2 | 0.12523 | 0.201615 | 0.077784 | ACT-PQ | gametocytes (pcr) |
| 28 | 12 | 6 | 6 | 0.062615 | 0.131175 | 0.029889 | ACT-PQ | gametocytes (pcr) |
| 7 | 42 | 0 | 2 | 1 | 1 | 1 | ACT-TQ | gametocytes (pcr) |
| 14 | 40 | 9 | 0 | 0.775 | 0.915839 | 0.655819 | ACT-TQ | gametocytes (pcr) |
| 21 | 31 | 7 | 0 | 0.6 | 0.772753 | 0.465867 | ACT-TQ | gametocytes (pcr) |
| 28 | 24 | 10 | 14 | 0.35 | 0.533923 | 0.229434 | ACT-TQ | gametocytes (pcr) |
| 1 | 89 | 6 | 1 | 0.932584 | 0.986159 | 0.88192 | DHA-PPQ | infectivity |
| 2 | 82 | 26 | 1 | 0.636887 | 0.74568 | 0.543967 | DHA-PPQ | infectivity |
| 7 | 55 | 19 | 11 | 0.416871 | 0.534451 | 0.32516 | DHA-PPQ | infectivity |
| 10 | 25 | 5 | 0 | 0.333497 | 0.457646 | 0.243027 | DHA-PPQ | infectivity |
| 14 | 20 | 7 | 3 | 0.216773 | 0.340372 | 0.138056 | DHA-PPQ | infectivity |
| 21 | 10 | 6 | 0 | 0.086709 | 0.209687 | 0.035856 | DHA-PPQ | infectivity |
| 28 | 4 | 3 | 0 | 0.021677 | 0.146879 | 0.003199 | DHA-PPQ | infectivity |
| 35 | 1 | 1 | 0 | 0 |  |  | DHA-PPQ | infectivity |
| 2 | 44 | 14 | 1 | 0.681818 | 0.834315 | 0.557195 | SP-AQ | infectivity |
| 5 | 29 | 3 | 0 | 0.611285 | 0.774536 | 0.482443 | SP-AQ | infectivity |
| 7 | 26 | 6 | 9 | 0.470219 | 0.645473 | 0.34255 | SP-AQ | infectivity |
| 14 | 11 | 5 | 0 | 0.256483 | 0.47946 | 0.137204 | SP-AQ | infectivity |
| 21 | 6 | 3 | 1 | 0.128242 | 0.354108 | 0.046443 | SP-AQ | infectivity |
| 28 | 2 | 2 | 0 | 0 |  |  | SP-AQ | infectivity |
| 2 | 32 | 9 | 0 | 0.71875 | 0.892699 | 0.578696 | PY-AS | infectivity |
| 7 | 23 | 8 | 0 | 0.46875 | 0.677847 | 0.324154 | PY-AS | infectivity |
| 10 | 15 | 7 | 0 | 0.25 | 0.455582 | 0.137187 | PY-AS | infectivity |
| 14 | 8 | 4 | 0 | 0.125 | 0.312625 | 0.04998 | PY-AS | infectivity |
| 21 | 4 | 2 | 0 | 0.0625 | 0.239143 | 0.016334 | PY-AS | infectivity |
| 28 | 2 | 1 | 0 | 0.03125 | 0.215103 | 0.00454 | PY-AS | infectivity |
| 35 | 1 | 1 | 0 | 0 |  |  | PY-AS | infectivity |
| 2 | 22 | 5 | 0 | 0.772727 | 0.969272 | 0.616037 | AS-AQ | infectivity |
| 7 | 17 | 10 | 0 | 0.318182 | 0.586584 | 0.172592 | AS-AQ | infectivity |
| 14 | 7 | 5 | 0 | 0.090909 | 0.34079 | 0.024251 | AS-AQ | infectivity |
| 21 | 2 | 1 | 0 | 0.045455 | 0.308467 | 0.006698 | AS-AQ | infectivity |
| 28 | 1 | 0 | 1 | 0.045455 | 0.308467 | 0.006698 | AS-AQ | infectivity |
| 2 | 59 | 52 | 0 | 0.118644 | 0.237839 | 0.059185 | AL | infectivity |
| 5 | 7 | 3 | 0 | 0.067797 | 0.174634 | 0.02632 | AL | infectivity |
| 7 | 4 | 4 | 0 | 0 |  |  | AL | infectivity |
| 2 | 21 | 20 | 0 | 0.047619 | 0.322454 | 0.007032 | Non-ACT-PQ | infectivity |
| 7 | 1 | 1 | 0 | 0 |  |  | Non-ACT-PQ | infectivity |
| 1 | 138 | 19 | 0 | 0.862319 | 0.921767 | 0.806705 | ACT-PQ | infectivity |
| 2 | 119 | 115 | 0 | 0.028986 | 0.076132 | 0.011036 | ACT-PQ | infectivity |
| 5 | 4 | 1 | 0 | 0.021739 | 0.066575 | 0.007099 | ACT-PQ | infectivity |
| 7 | 3 | 3 | 0 | 0 |  |  | ACT-PQ | infectivity |
| 2 | 40 | 12 | 0 | 0.7 | 0.857444 | 0.571466 | ACT-TQ | infectivity |
| 7 | 28 | 26 | 0 | 0.05 | 0.193028 | 0.012951 | ACT-TQ | infectivity |
| 14 | 2 | 2 | 0 | 0 |  |  | ACT-TQ | infectivity |
| 1 | 82 | 7 | 0 | 0.914634 | 0.977158 | 0.856111 | DHA-PPQ | gametocytes (microscopy) |
| 2 | 75 | 6 | 0 | 0.841463 | 0.92435 | 0.766009 | DHA-PPQ | gametocytes (microscopy) |
| 3 | 69 | 2 | 1 | 0.817073 | 0.905186 | 0.737538 | DHA-PPQ | gametocytes (microscopy) |
| 7 | 66 | 12 | 0 | 0.668514 | 0.779071 | 0.573647 | DHA-PPQ | gametocytes (microscopy) |
| 10 | 54 | 3 | 0 | 0.631375 | 0.745498 | 0.534722 | DHA-PPQ | gametocytes (microscopy) |
| 14 | 51 | 15 | 0 | 0.445676 | 0.5682 | 0.349573 | DHA-PPQ | gametocytes (microscopy) |
| 21 | 36 | 7 | 0 | 0.359017 | 0.480336 | 0.26834 | DHA-PPQ | gametocytes (microscopy) |
| 28 | 29 | 21 | 0 | 0.099039 | 0.191165 | 0.05131 | DHA-PPQ | gametocytes (microscopy) |
| 35 | 8 | 3 | 0 | 0.061899 | 0.144659 | 0.026487 | DHA-PPQ | gametocytes (microscopy) |
| 42 | 5 | 5 | 0 | 0 |  |  | DHA-PPQ | gametocytes (microscopy) |
| 1 | 45 | 1 | 0 | 0.977778 | 1 | 0.935644 | SP-AQ | gametocytes (microscopy) |
| 2 | 44 | 5 | 0 | 0.866667 | 0.971902 | 0.772826 | SP-AQ | gametocytes (microscopy) |
| 3 | 39 | 1 | 1 | 0.844444 | 0.957264 | 0.744921 | SP-AQ | gametocytes (microscopy) |
| 5 | 37 | 3 | 0 | 0.775976 | 0.908573 | 0.66273 | SP-AQ | gametocytes (microscopy) |
| 7 | 34 | 3 | 0 | 0.707508 | 0.854922 | 0.585512 | SP-AQ | gametocytes (microscopy) |
| 14 | 31 | 7 | 0 | 0.547748 | 0.716282 | 0.418868 | SP-AQ | gametocytes (microscopy) |
| 21 | 24 | 7 | 0 | 0.387988 | 0.562439 | 0.267646 | SP-AQ | gametocytes (microscopy) |
| 28 | 17 | 9 | 4 | 0.182583 | 0.341507 | 0.097615 | SP-AQ | gametocytes (microscopy) |
| 42 | 4 | 4 | 0 | 0 |  |  | SP-AQ | gametocytes (microscopy) |
| 2 | 33 | 2 | 0 | 0.939394 | 1 | 0.861413 | PY-AS | gametocytes (microscopy) |
| 7 | 31 | 7 | 0 | 0.727273 | 0.896263 | 0.590145 | PY-AS | gametocytes (microscopy) |
| 10 | 24 | 4 | 0 | 0.606061 | 0.797956 | 0.460313 | PY-AS | gametocytes (microscopy) |
| 14 | 20 | 7 | 1 | 0.393939 | 0.601476 | 0.258012 | PY-AS | gametocytes (microscopy) |
| 21 | 12 | 1 | 0 | 0.361111 | 0.569902 | 0.228813 | PY-AS | gametocytes (microscopy) |
| 28 | 11 | 8 | 0 | 0.098485 | 0.28639 | 0.033867 | PY-AS | gametocytes (microscopy) |
| 35 | 3 | 3 | 0 | 0 |  |  | PY-AS | gametocytes (microscopy) |
| 2 | 24 | 1 | 0 | 0.958333 | 1 | 0.881631 | AS-AQ | gametocytes (microscopy) |
| 7 | 23 | 3 | 1 | 0.833333 | 0.996604 | 0.696811 | AS-AQ | gametocytes (microscopy) |
| 14 | 19 | 7 | 0 | 0.526316 | 0.775213 | 0.357332 | AS-AQ | gametocytes (microscopy) |
| 21 | 12 | 5 | 0 | 0.307018 | 0.568055 | 0.165934 | AS-AQ | gametocytes (microscopy) |
| 28 | 7 | 6 | 1 | 0.04386 | 0.297989 | 0.006456 | AS-AQ | gametocytes (microscopy) |
| 2 | 71 | 18 | 1 | 0.746479 | 0.854847 | 0.651848 | AL | gametocytes (microscopy) |
| 5 | 52 | 5 | 0 | 0.674702 | 0.793331 | 0.573812 | AL | gametocytes (microscopy) |
| 7 | 47 | 16 | 0 | 0.445016 | 0.578062 | 0.342592 | AL | gametocytes (microscopy) |
| 14 | 31 | 17 | 0 | 0.200975 | 0.320871 | 0.125879 | AL | gametocytes (microscopy) |
| 21 | 14 | 10 | 0 | 0.057421 | 0.14866 | 0.02218 | AL | gametocytes (microscopy) |
| 28 | 4 | 2 | 2 | 0.028711 | 0.112506 | 0.007327 | AL | gametocytes (microscopy) |
| 3 | 21 | 2 | 1 | 0.904762 | 1 | 0.787535 | Non-ACT-PQ | gametocytes (microscopy) |
| 7 | 18 | 12 | 0 | 0.301587 | 0.588134 | 0.15465 | Non-ACT-PQ | gametocytes (microscopy) |
| 14 | 6 | 5 | 0 | 0.050265 | 0.33937 | 0.007445 | Non-ACT-PQ | gametocytes (microscopy) |
| 42 | 1 | 1 | 0 | 0 |  |  | Non-ACT-PQ | gametocytes (microscopy) |
| 2 | 108 | 21 | 1 | 0.805556 | 0.883765 | 0.734268 | ACT-PQ | gametocytes (microscopy) |
| 5 | 86 | 15 | 0 | 0.665052 | 0.760604 | 0.581503 | ACT-PQ | gametocytes (microscopy) |
| 7 | 71 | 61 | 0 | 0.093669 | 0.168973 | 0.051925 | ACT-PQ | gametocytes (microscopy) |
| 14 | 10 | 10 | 0 | 0 |  |  | ACT-PQ | gametocytes (microscopy) |
| 1 | 48 | 7 | 0 | 0.854167 | 0.960082 | 0.759936 | ACT-TQ | gametocytes (microscopy) |
| 2 | 41 | 10 | 0 | 0.645833 | 0.796347 | 0.523767 | ACT-TQ | gametocytes (microscopy) |
| 7 | 31 | 16 | 1 | 0.3125 | 0.475424 | 0.205409 | ACT-TQ | gametocytes (microscopy) |
| 14 | 14 | 11 | 0 | 0.066964 | 0.198628 | 0.022576 | ACT-TQ | gametocytes (microscopy) |
| 21 | 3 | 3 | 0 | 0 |  |  | ACT-TQ | gametocytes (microscopy) |

Kaplan-Meier survival data showing the cumulative probability of remaining uncleared of gametocytes detected by microscopy, gametocytes detected by RT-qPCR and infectivity to mosquitos over time stratified across different antimalarial treatment categories (DHA-PPQ, SP-AQ, PY-AS, AS-AQ, AL, Non-ACT-PQ, ACT-PQ, ACT-TQ). The number of individuals at risk per timepoint is presented (n.risk), as well as the number of clearance events (n.event) and the number of censored observations (n.censor).

## Table O. Hazard ratios for infectivity survival curves (adjusted by baseline PCR gametocyte densities)

| Reference | DHA-PPQ | SP-AQ | PY-AS | AS-AQ | AL | Non-ACT-PQ | ACT-PQ | ACT-TQ |
| --- | --- | --- | --- | --- | --- | --- | --- | --- |
| DHA-PPQ |  | 1.09 (0.47 - 2.51) p=0.8491 | 1.32 (0.61 - 2.87) p=0.4766 | ᵃ1.04 (0.35 - 3.12) p=0.9402 | ᵃ6.12 (2.69 - 13.92) p<0.0001 | 6.90 (2.42 - 19.66) p=0.0003 | 7.72 (4.13 - 14.44) p<0.0001 | 1.58 (0.68 - 3.68) p=0.2856 |
| SP-AQ | 0.92 (0.40 - 2.14) p=0.8491 |  | ᵃ1.22 (0.42 - 3.55) p=0.7146 | ᵃ0.96 (0.29 - 3.20) p=0.9484 | 5.64 (2.24 - 14.22) p=0.0002 | 6.36 (2.29 - 17.66) p=0.0004 | 7.12 (3.04 - 16.63) p<0.0001 | ᵃ1.46 (0.44 - 4.80) p=0.5342 |
| PY-AS | 0.76 (0.35 - 1.64) p=0.4766 | ᵃ0.82 (0.28 - 2.38) p=0.7146 |  | ᵃ0.79 (0.23 - 2.65) p=0.6995 | ᵃ4.62 (1.74 - 12.29) p=0.0021 | ᵃ5.21 (1.47 - 18.50) p=0.0106 | 5.83 (2.61 - 13.04) p<0.0001 | ᵃ1.20 (0.38 - 3.75) p=0.7600 |
| AS-AQ | ᵃ0.96 (0.32 - 2.87) p=0.9402 | ᵃ1.04 (0.31 - 3.46) p=0.9484 | ᵃ1.27 (0.38 - 4.27) p=0.6995 |  | 5.87 (2.40 - 14.34) p=0.0001 | ᵃ6.62 (1.58 - 27.70) p=0.0096 | 7.40 (2.96 - 18.53) p<0.0001 | ᵃ1.52 (0.38 - 6.05) p=0.5542 |
| AL | ᵃ0.16 (0.07 - 0.37) p<0.0001 | 0.18 (0.07 - 0.45) p=0.0002 | ᵃ0.22 (0.08 - 0.58) p=0.0021 | 0.17 (0.07 - 0.42) p=0.0001 |  | ᵃ1.13 (0.33 - 3.82) p=0.8468 | 1.26 (0.70 - 2.26) p=0.4357 | ᵃ0.26 (0.08 - 0.84) p=0.0243 |
| Non-ACT-PQ | 0.14 (0.05 - 0.41) p=0.0003 | 0.16 (0.06 - 0.44) p=0.0004 | ᵃ0.19 (0.05 - 0.68) p=0.0106 | ᵃ0.15 (0.04 - 0.63) p=0.0096 | ᵃ0.89 (0.26 - 3.00) p=0.8468 |  | ᵃ1.12 (0.36 - 3.47) p=0.8465 | ᵃ0.23 (0.06 - 0.88) p=0.0317 |
| ACT-PQ | 0.13 (0.07 - 0.24) p<0.0001 | 0.14 (0.06 - 0.33) p<0.0001 | 0.17 (0.08 - 0.38) p<0.0001 | 0.14 (0.05 - 0.34) p<0.0001 | 0.79 (0.44 - 1.42) p=0.4357 | ᵃ0.89 (0.29 - 2.78) p=0.8465 |  | ᵃ0.21 (0.07 - 0.59) p=0.0031 |
| ACT-TQ | 0.63 (0.27 - 1.47) p=0.2856 | ᵃ0.69 (0.21 - 2.25) p=0.5342 | ᵃ0.84 (0.27 - 2.63) p=0.7600 | ᵃ0.66 (0.17 - 2.63) p=0.5542 | ᵃ3.87 (1.19 - 12.55) p=0.0243 | ᵃ4.36 (1.14 - 16.72) p=0.0317 | ᵃ4.88 (1.71 - 13.94) p=0.0031 |  |

Hazard ratios for between-arm comparisons of infectivity clearance, with corresponding 95% CI and p-value.

E.g. Hazard ratio of infectivity clearance for AL compared to DHA-PPQ is 6.12, meaning that clearance is 6.12 times more likely to take place in the AL group compared to the DHA-PPQ group, and this is significantly different (p<0.0001).

^a^ = Indirect comparison in the network meta-analysis.

## Table P. Hazard ratios for gametocytes by microscopy survival curves

| Reference | DHA-PPQ | SP-AQ | PY-AS | AS-AQ | AL | Non-ACT-PQ | ACT-PQ | ACT-TQ |
| --- | --- | --- | --- | --- | --- | --- | --- | --- |
| DHA-PPQ |  | 1.25 (0.74 - 2.10) p=0.4048 | 1.64 (1.00 - 2.69) p=0.0505 | ᵃ1.21 (0.58 - 2.52) p=0.6129 | ᵃ2.60 (1.46 - 4.65) p=0.0012 | 3.14 (1.68 - 5.85) p=0.0003 | 8.26 (4.96 - 13.73) p<0.0001 | 1.73 (1.05 - 2.86) p=0.0330 |
| SP-AQ | 0.80 (0.48 - 1.35) p=0.4048 |  | ᵃ1.31 (0.70 - 2.48) p=0.3999 | ᵃ0.97 (0.48 - 1.97) p=0.9301 | 2.09 (1.23 - 3.53) p=0.0062 | 2.52 (1.34 - 4.71) p=0.0040 | 6.61 (3.90 - 11.23) p<0.0001 | ᵃ1.39 (0.67 - 2.86) p=0.3784 |
| PY-AS | 0.61 (0.37 - 1.00) p=0.0505 | ᵃ0.76 (0.40 - 1.44) p=0.3999 |  | ᵃ0.74 (0.35 - 1.57) p=0.4307 | ᵃ1.59 (0.86 - 2.94) p=0.1396 | ᵃ1.92 (0.90 - 4.09) p=0.0934 | 5.04 (3.01 - 8.44) p<0.0001 | ᵃ1.05 (0.52 - 2.14) p=0.8822 |
| AS-AQ | ᵃ0.83 (0.40 - 1.73) p=0.6129 | ᵃ1.03 (0.51 - 2.10) p=0.9301 | ᵃ1.36 (0.64 - 2.89) p=0.4307 |  | 2.15 (1.28 - 3.63) p=0.0039 | ᵃ2.60 (1.07 - 6.28) p=0.0341 | 6.83 (3.83 - 12.17) p<0.0001 | ᵃ1.43 (0.59 - 3.49) p=0.4317 |
| AL | ᵃ0.38 (0.22 - 0.69) p=0.0012 | 0.48 (0.28 - 0.81) p=0.0062 | ᵃ0.63 (0.34 - 1.16) p=0.1396 | 0.46 (0.28 - 0.78) p=0.0039 |  | ᵃ1.21 (0.57 - 2.55) p=0.6247 | 3.17 (2.15 - 4.67) p<0.0001 | ᵃ0.66 (0.31 - 1.43) p=0.2959 |
| Non-ACT-PQ | 0.32 (0.17 - 0.59) p=0.0003 | 0.40 (0.21 - 0.74) p=0.0040 | ᵃ0.52 (0.24 - 1.12) p=0.0934 | ᵃ0.39 (0.16 - 0.93) p=0.0341 | ᵃ0.83 (0.39 - 1.76) p=0.6247 |  | ᵃ2.63 (1.28 - 5.42) p=0.0088 | ᵃ0.55 (0.25 - 1.23) p=0.1442 |
| ACT-PQ | 0.12 (0.07 - 0.20) p<0.0001 | 0.15 (0.09 - 0.26) p<0.0001 | 0.20 (0.12 - 0.33) p<0.0001 | 0.15 (0.08 - 0.26) p<0.0001 | 0.32 (0.21 - 0.46) p<0.0001 | ᵃ0.38 (0.18 - 0.78) p=0.0088 |  | ᵃ0.21 (0.10 - 0.43) p<0.0001 |
| ACT-TQ | 0.58 (0.35 - 0.96) p=0.0330 | ᵃ0.72 (0.35 - 1.49) p=0.3784 | ᵃ0.95 (0.47 - 1.92) p=0.8822 | ᵃ0.70 (0.29 - 1.71) p=0.4317 | ᵃ1.51 (0.70 - 3.25) p=0.2959 | ᵃ1.82 (0.82 - 4.04) p=0.1442 | ᵃ4.78 (2.33 - 9.77) p<0.0001 |  |

Hazard ratios for between-arm comparisons of microscopical gametocyte clearance, with corresponding 95% CI and p-value.

E.g. Hazard ratio of microscopical gametocyte clearance for AL compared to DHA-PPQ is 2.60, meaning that clearance is 2.60 times more likely to take place in the AL group compared to the DHA-PPQ group, and this is significantly different (p=0.0012).

^a^ = Indirect comparison in the network meta-analysis.

## Table Q. Hazard ratios for gametocytes by RT-qPCR survival curves

| Reference | DHA-PPQ | SP-AQ | PY-AS | AS-AQ | AL | Non-ACT-PQ | ACT-PQ | ACT-TQ |
| --- | --- | --- | --- | --- | --- | --- | --- | --- |
| DHA-PPQ |  | 0.70 (0.25 - 1.92) p=0.4863 | 0.81 (0.44 - 1.48) p=0.4944 | ᵃ 0.48 (0.13 - 1.80) p=0.2765 | ᵃ 0.92 (0.36 - 2.36) p=0.8575 | 6.86 (2.93 - 16.03) p<0.0001 | 13.63 (6.36 - 29.21) p<0.0001 | 19.73 (4.46 - 87.35) p<0.0001 |
| SP-AQ | 1.43 (0.52 - 3.95) p=0.4863 |  | ᵃ 1.16 (0.36 - 3.78) p=0.8022 | ᵃ 0.69 (0.13 - 3.65) p=0.6587 | ᵃ 1.31 (0.33 - 5.27) p=0.6993 | 9.83 (3.61 - 26.80) p<0.0001 | ᵃ19.54 (5.49 - 69.49) p<0.0001 | ᵃ28.29 (4.67 - 171.24) p=0.0003 |
| PY-AS | 1.23 (0.68 - 2.25) p=0.4944 | ᵃ 0.86 (0.26 - 2.80) p=0.8022 |  | ᵃ 0.59 (0.15 - 2.29) p=0.4455 | ᵃ 1.13 (0.42 - 3.03) p=0.8069 | ᵃ 8.46 (2.99 - 23.94) p<0.0001 | 16.81 (7.49 - 37.74) p<0.0001 | ᵃ24.33 (4.89 - 121.08) p<0.0001 |
| AS-AQ | ᵃ 2.09 (0.55 - 7.87) p=0.2765 | ᵃ 1.46 (0.27 - 7.74) p=0.6587 | ᵃ 1.69 (0.44 - 6.56) p=0.4455 |  | 1.92 (0.65 - 5.68) p=0.2416 | ᵃ14.33 (2.97 - 69.23) p=0.0009 | 28.47 (9.61 - 84.35) p<0.0001 | ᵃ41.22 (5.61 - 302.58) p=0.0003 |
| AL | ᵃ 1.09 (0.42 - 2.81) p=0.8575 | ᵃ 0.76 (0.19 - 3.05) p=0.6993 | ᵃ 0.88 (0.33 - 2.37) p=0.8069 | 0.52 (0.18 - 1.55) p=0.2416 |  | ᵃ 7.48 (2.10 - 26.69) p=0.0019 | 14.87 (8.47 - 26.09) p<0.0001 | ᵃ21.52 (3.69 - 125.55) p=0.0006 |
| Non-ACT-PQ | 0.15 (0.06 - 0.34) p<0.0001 | 0.10 (0.04 - 0.28) p<0.0001 | ᵃ 0.12 (0.04 - 0.33) p<0.0001 | ᵃ 0.07 (0.01 - 0.34) p=0.0009 | ᵃ 0.13 (0.04 - 0.48) p=0.0019 |  | ᵃ 1.99 (0.63 - 6.22) p=0.2382 | ᵃ 2.88 (0.52 - 15.95) p=0.2267 |
| ACT-PQ | 0.07 (0.03 - 0.16) p<0.0001 | ᵃ 0.05 (0.01 - 0.18) p<0.0001 | 0.06 (0.03 - 0.13) p<0.0001 | 0.04 (0.01 - 0.10) p<0.0001 | 0.07 (0.04 - 0.12) p<0.0001 | ᵃ 0.50 (0.16 - 1.58) p=0.2382 |  | ᵃ 1.45 (0.27 - 7.70) p=0.6645 |
| ACT-TQ | 0.05 (0.01 - 0.22) p<0.0001 | ᵃ 0.04 (0.01 - 0.21) p=0.0003 | ᵃ 0.04 (0.01 - 0.20) p<0.0001 | ᵃ 0.02 (0.00 - 0.18) p=0.0003 | ᵃ 0.05 (0.01 - 0.27) p=0.0006 | ᵃ 0.35 (0.06 - 1.93) p=0.2267 | ᵃ 0.69 (0.13 - 3.68) p=0.6645 |  |

Hazard ratios for between-arm comparisons of molecular gametocyte clearance, with corresponding 95% CI and p-value.

E.g. Hazard ratio of molecular gametocyte clearance for AL compared to DHA-PPQ is 0.92, meaning that clearance is 0.92 times more likely to take place in the AL group compared to the DHA-PPQ group, and this is significantly different (p=0.8575).

^a^ = Indirect comparison in the network meta-analysis.

## Fig R. Forest plots of treatment comparisons of infectivity survival curves (adjusted by baseline RT-qPCR gametocyte densities) at days 2, 7, and 14. All comparisons are with ACT-PQ.

**
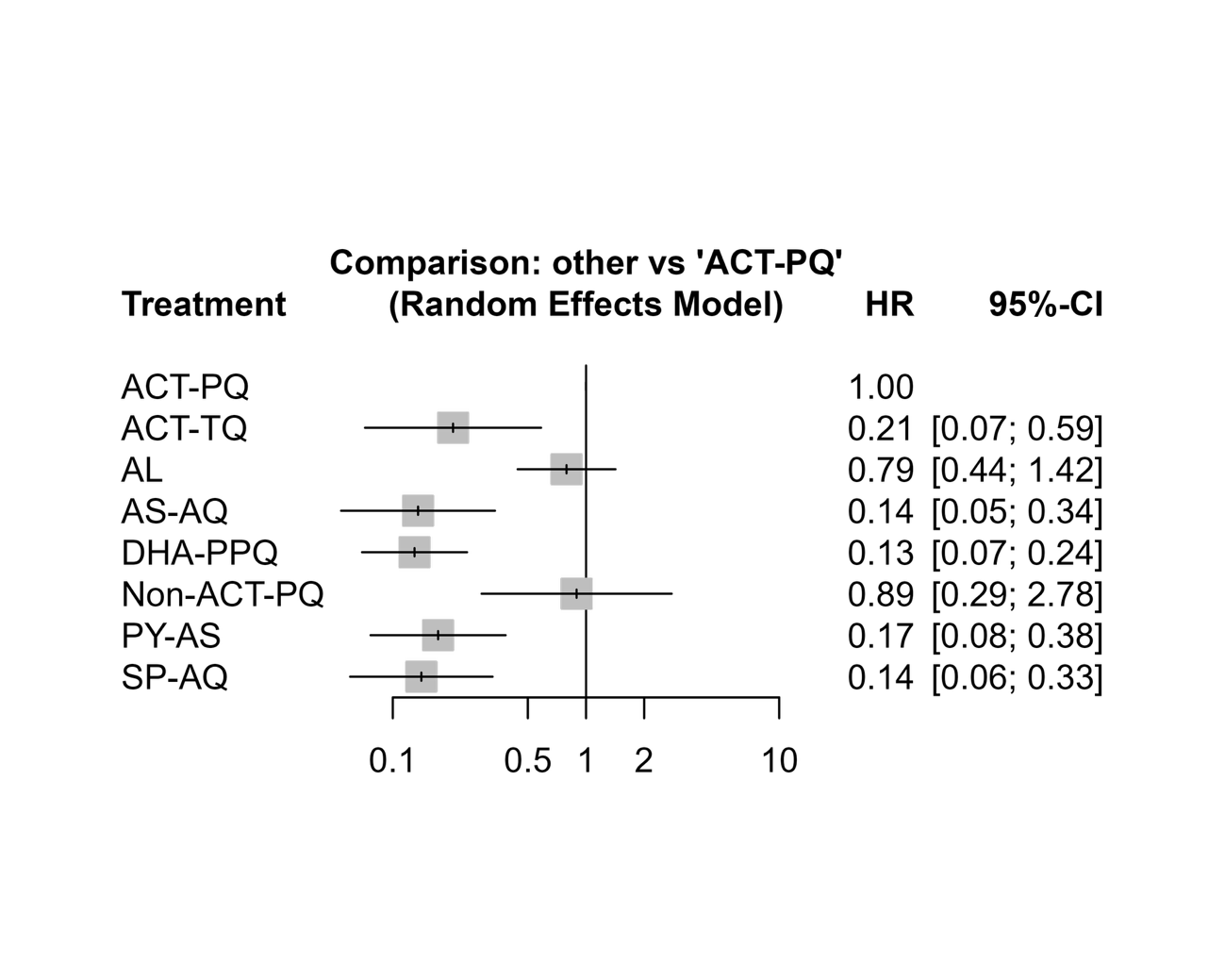
**

Results from the network meta-analysis are shown as mean differences (MD) in relative reductions from baseline in infectivity survival curve, with 95% confidence intervals. Each treatment is compared to ACT-PQ, the reference treatment. Negative values indicate a smaller reduction than ACT-PQ, while positive values indicate a larger reduction. Point estimates are plotted as squares proportional to study weight, and horizontal lines denote confidence intervals.

## Fig S. Forest plots of treatment comparisons of microscopy gametocyte density survival curves at days 2, 7, and 14. All comparisons are with ACT-PQ.


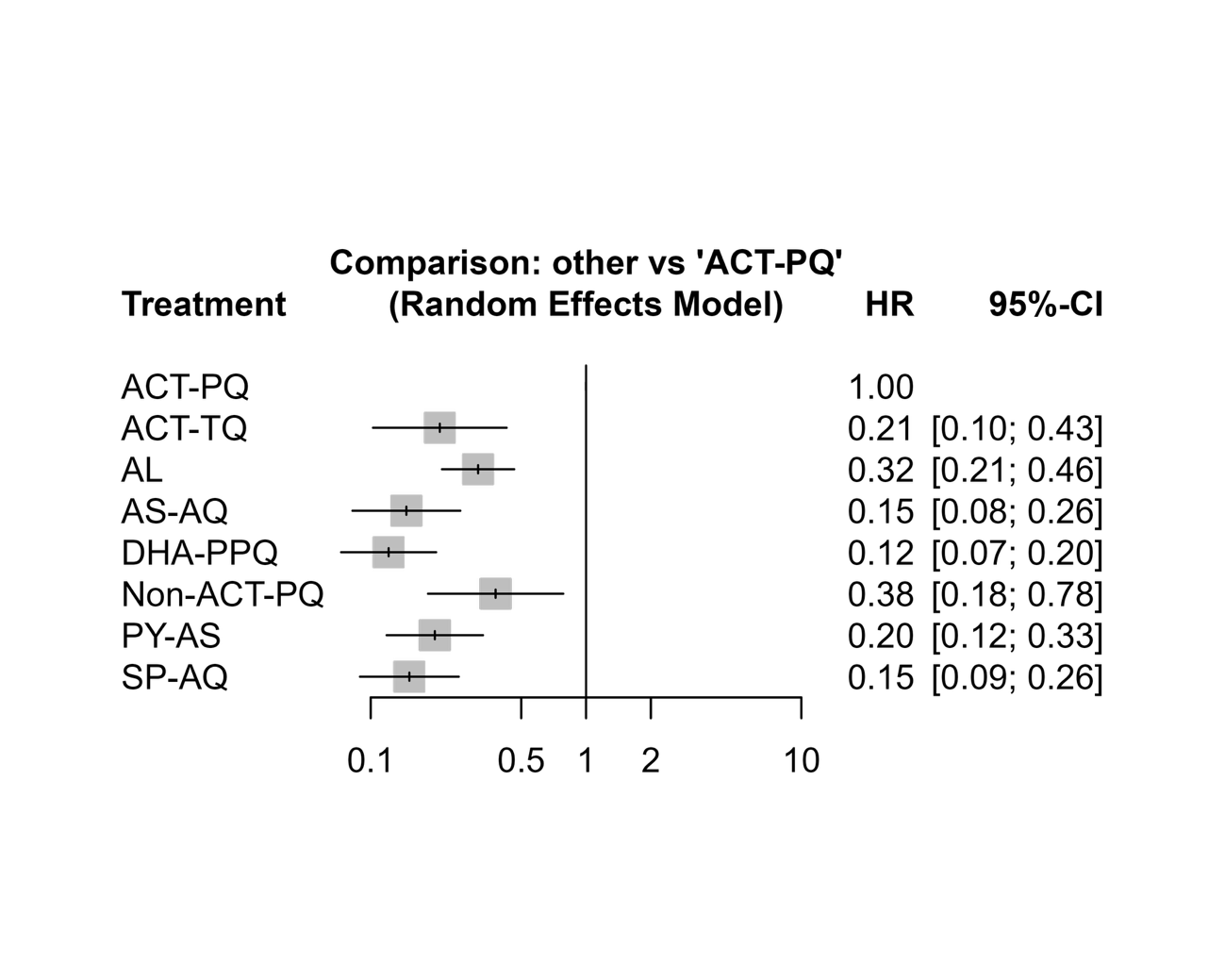


Results from the network meta-analysis are shown as mean differences (MD) in relative reductions from baseline in gametocyte density survival curve, with 95% confidence intervals. Each treatment is compared to ACT-PQ, the reference treatment. Negative values indicate a smaller reduction than ACT-PQ, while positive values indicate a larger reduction. Point estimates are plotted as squares proportional to study weight, and horizontal lines denote confidence intervals.

## Fig T. Forest plots of treatment comparisons of molecular gametocyte density survival curves at days 2, 7, and 14. All comparisons are with ACT-PQ.


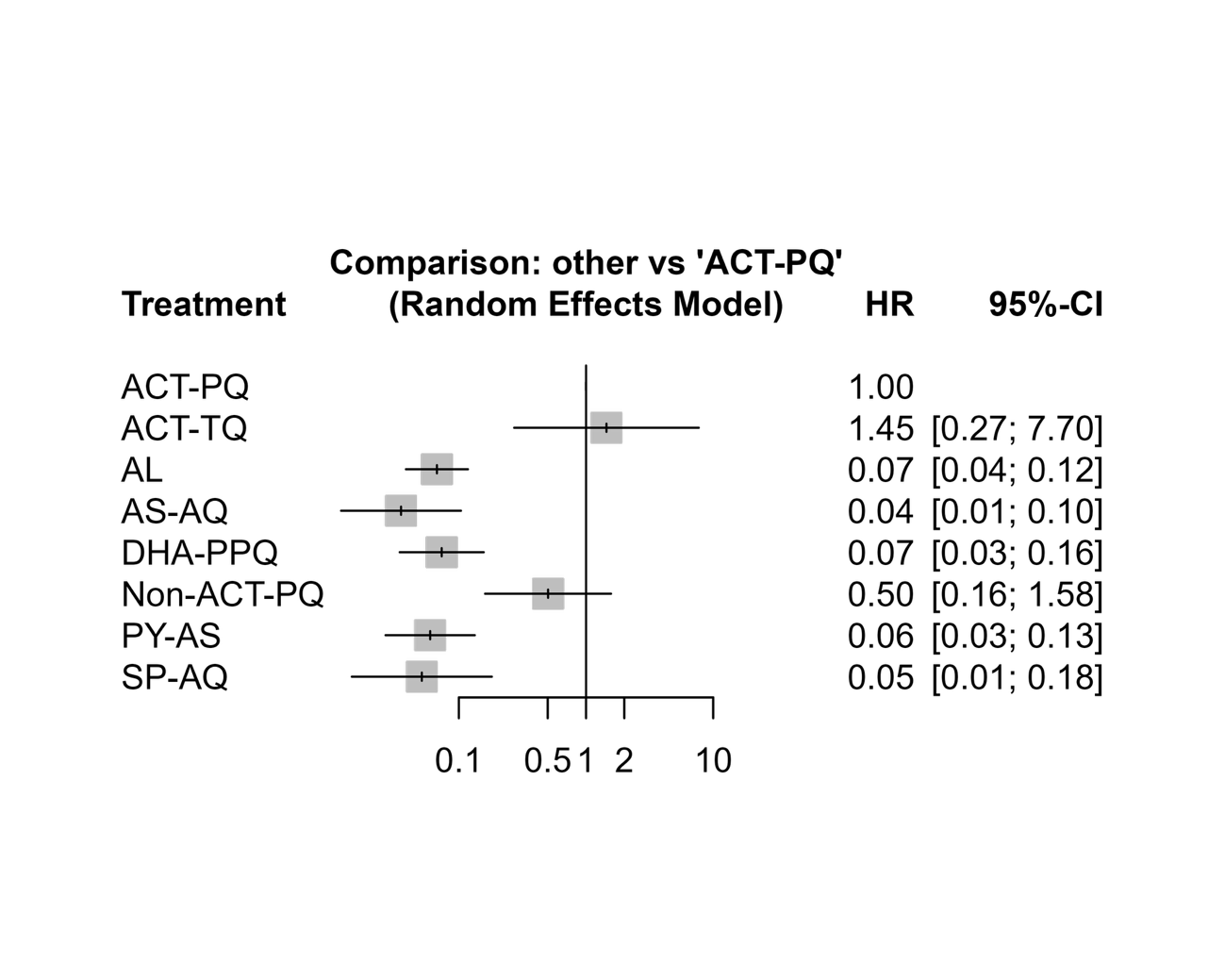


Results from the network meta-analysis are shown as mean differences (MD) in relative reductions from baseline in molecular gametocyte density survival curve, with 95% confidence intervals. Each treatment is compared to ACT-PQ, the reference treatment. Negative values indicate a smaller reduction than ACT-PQ, while positive values indicate a larger reduction. Point estimates are plotted as squares proportional to study weight, and horizontal lines denote confidence intervals.

## Fig U. Consistency assessment comparing direct and indirect treatment effects for proportion infected mosquitoes


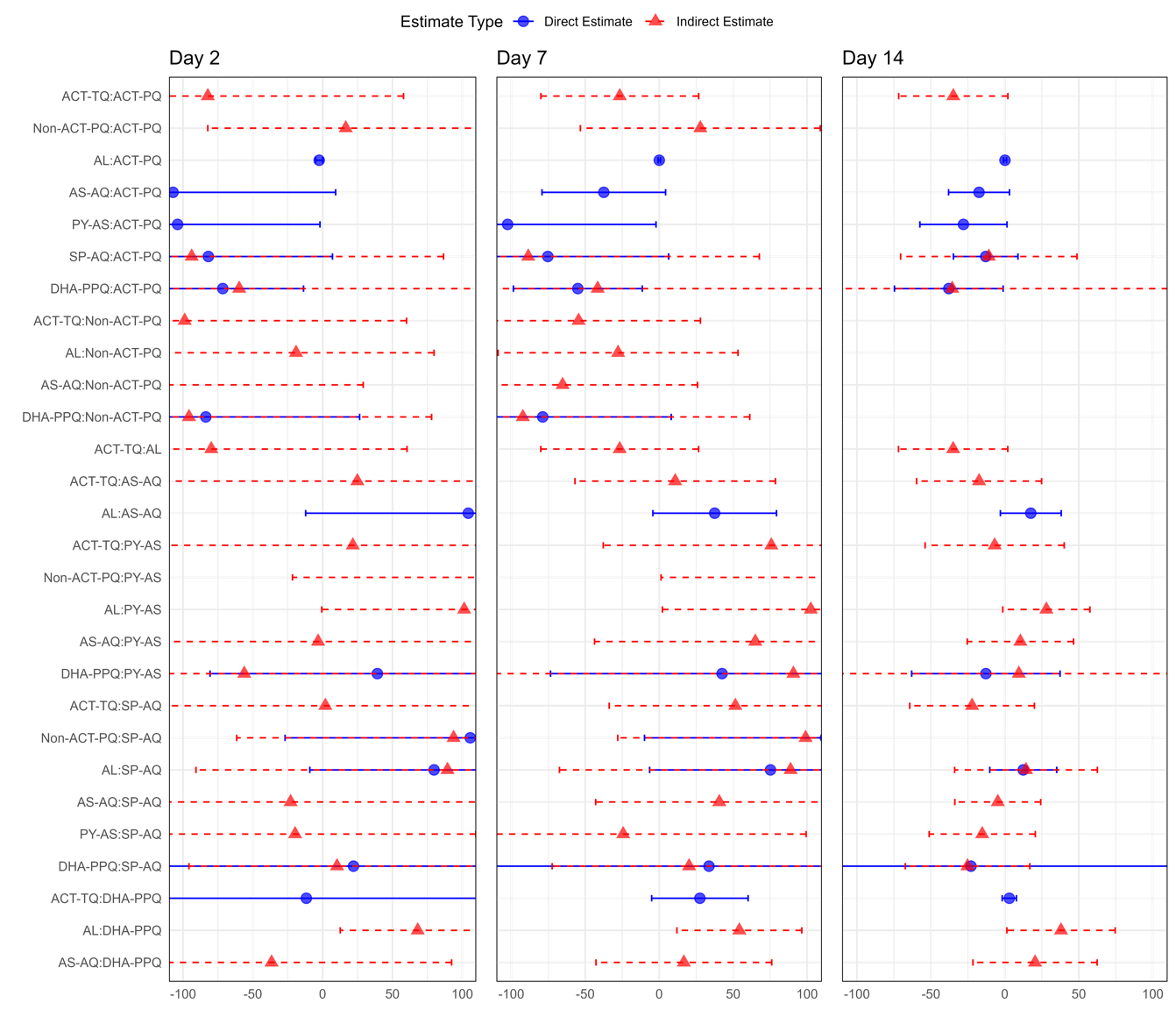


**Consistency assessment comparing direct (blue) and indirect (red) treatment effects for proportion infected mosquitoes.** For each pairwise comparison, the direct effect estimate (based on head-to-head trials) and the indirect effect estimate (derived from the network via a common comparator) are plotted with their corresponding 95% confidence intervals. Overlapping intervals suggest consistency between direct and indirect evidence, supporting the validity of the network meta-analysis assumptions.
