# Supplementary material for: The transmission blocking activity of artemisinin-combination, non-artemisinin, and 8-aminoquinoline antimalarial therapies: A pooled analysis of individual participant data
Source: PLoS Med. 2025 Aug 14;22(8):e1004683. doi: 10.1371/journal.pmed.1004683 (PMC12352847; doi:10.1371/journal.pmed.1004683)
Supplement: Attachment — Submitted filename: vanheer.pdf [file pmed.1004683.s006.pdf]

This paper describes a collaborative reanalysis of six trials of blocking of transmission of malaria.

I was asked for a methodological report and I interpret that to include all aspects of the design and conduct of the study. I see that I initially reviewed this paper some time back. I understand that the editorial decision was to reject it but to be open to a new substantially changed version. I have therefore decided to review it from scratch and only then to check whether any of my original points are still relevant and if so whether they have been answered. The version I received did not have page numbers. My numbering has the abstract starting on page 3

1 Review of current submission

1.1 Points of detail

Page 10 Choosing not to evaluate arms which do not correspond to current practice risks missing some novel effect. Conventional wisdom is not always right. I think this is more an issue for the clinical reviewers. If they agree then omitting those arms is fine.

Page 13 and Figures 2A and B I do not understand why some of these are truncated below and indeed in 2A why the first one is truncated above.

Page 13 and Figure 2C I find it hard to see what I am being told here. If this is just presenting proportion infected per participant why was a logistic regression needed? Given the numbers of participants per trial why not just plot the proportions per participant?

Page 14 and Figure 3A I agree this shows excellent agreement between the two methods.

Page 14 '[...] could be observed in the ACT–PQ group only at seven days [...]' is ambiguous. Does it mean that was the only time that group achieved statistical significance or does it mean that was the only group that achieved statistical significance at that time point. The English word 'only' is a slippery customer. Perhaps re–write the sentence?

Page 14 and Figures 3B to D Are the authors trying, by comparing above and below some level of statistical significance, to imply that there is a difference? If so that is not the way to do it (Gelman and Stern, 2006).

Page 14 and Figures 3B to D I do not understand the comment in the caption about truncating the y–axis. Why were the standard errors

inflated? This must tell us something about either the data or the model.

Page 16 and Figure 4B I do not see the PY–AS group failing to show a difference at day 14 as the confidence interval does not include 0.

Pages 16 and 17 Is this supposed to be a report of the NMA? I would make that explicit. In Figure 5 it would be much better to have the effect size in the cells rather than a p–value. Are these the P–scores produced by the netrank function in netmeta perhaps?

Page 19 The NMA is discussed here but it should have been presented in the results section.

1.2 Point of more substance

As it stands the paper does not seem to have taken full advantage of the NMA unless I have missed something. It is discussed but without any attempt at presenting it fully. Since comparing the treatment regimes and recommending a best buy seems a goal I would have thought the analysis known in the Bayesain community as SUCRA was almost mandatory. A frequentist analogue is provided in netmeta.

In addition for an example of the sort of plots I would have expected to see one might look at Barth et al. (2013). The relevant PRISMA guidelines (Hutton et al., 2015) are also relevant.

1.3 Summary

Given the careful and meticulous presentation of the remainder of the analysis I find the almost total absence of the NMA results quite baffling. It is almost as though there is a whole section which was written but then left out.

2 Response to rebuttal comments

These do not seem to me to add any important issues which I have not addressed above.

3 Overall summary

Clearly a high quality piece of work. There are a few points which I have rasied for clarification but other than those it is thoroughly explained except for the NMA.

Michael Dewey

References

J Barth, T Munder, H Gerger, E Nüesch, S Trelle, H Znoj, P Jüni, and P Cuijpers. Comparative efficacy of seven psychotherapeutic interventions for patients with depression: a network meta–analysis. *PLOS Medicine*, 10(5), 2013.

A Gelman and H Stern. The difference between 'significant' and 'not significant' is not itself statistically significant. *The American Statistician*, 60: 328–331, 2006.

B Hutton, G Salanti, D Caldwell, A Chaimani, C H Schmid, C Cameron, J P A Ioannidis, S Straus, K Thorlund, J Jansen, C Mulrow, F Catalá-López, P C Gøtzsche, K Dickersin, I Boutron, D G Altman, and D Moher. The PRISMA extension statement for reporting of systematic reviews incorporating network meta–analyses of health care interventions: checklist and explanations. *Annals of Internal Medicine*, 162:777–784, 2015.
