# Supplementary material for: The transmission blocking activity of artemisinin-combination, non-artemisinin, and 8-aminoquinoline antimalarial therapies: A pooled analysis of individual participant data
Source: PLoS Med. 2025 Aug 14;22(8):e1004683. doi: 10.1371/journal.pmed.1004683 (PMC12352847; doi:10.1371/journal.pmed.1004683)
Supplement: Attachment — Submitted filename: Response_Reviewers_final.docx [file pmed.1004683.s004.docx]

Dear Richard Turner,

We appreciate the rigorous review of our manuscript PMEDICINE-D-24-03072R1 and the invitation to submit a revised version of our manuscript (email 27-11-2024; Case Number: 08778544). The main editorial comment was to re-analyse the data based on the comments from the statistical reviewer. We have taken this comment very seriously and have re-analysed our data with the suggested methodology. Our original analysis was inspired by the analytical approach used by the WorldWide Antimalarial Resistance Network (WWARN) that regularly presents pooled analyses of individual data without using Network Meta-Analysis (NMA). We agree with the statistical reviewer that there is considerable benefit in NMA that provides more precise estimates of indirect treatment effects. We hereby submit out re-analysed data using the proposed methodology; our findings have not changed substantially but we agree that this additional work has greatly improved our work.

In addition to the three reviewers that were invited by PLoS Medicine, we also received two (very positive) reviews by Rapid Reviews Rapid Infectious Diseases in response to the MedRxiv submission (<https://rrid.mitpress.mit.edu/pub/sl98ip2v/release/1>). To do justice to all reviewers, we have included their comments in our revision and highlight these below. Below, you will find a point-by-point response to all the comments raised.

Yours sincerely,

Teun Bousema, on behalf of all authors.

**Reviewers' comments:**

***** Reviewer #1:** 

This study is well-written with well-produced Figures and provides an interesting analysis of differing transmission-blocking properties of various antimalarial drugs. The quantification of AL having increased gametocytocidal activity compared to other ACTs is particularly notable. 

However, some aspects of the text and data appeared unclear or incompletely explored, and comments requesting clarification of these are below (organised by section).


Introduction:
L74: The authors mention "schizonticidal" drugs, but is this still a suitable terminology for drugs targeting the asexual blood stages? Some drugs affect asexual blood stages other than schizonts, and therefore terminology describing this more precisely would be clearer.

Response: whilst schizonticidal is commonly used to describe anti-blood stage activity (as opposed to gametocytocidal), we agree that this is an imprecise term. We have now replaced this with ‘anti-asexual’ drugs in text and figure.

L177-178: It is understandable that the authors chose not to focus on arms which would not be used as treatment, such as methylene blue, but why was SP-AQ with SLD TQ not included in the analysis? This could have been interesting, and the authors should clarify why this decision was made.

Response: In the current analysis, we focused on a limited number of treatment regimens that are used or considered for use. SLD TQ was, at the time of conducting the analysis, not supported for Pf. After the initial study where we performed dose finding for SLD TQ, there was a clear tendency at the Bill & Melinda Gates Foundation and Medicine for Malaria Venture that TQ was considered for P. vivax only. We nevertheless included SLD TQ in combination with ACTs to formally analyse whether there is evidence for a delayed effect of TQ. We acknowledge that this is somewhat arbitrary and not supported by published strategies or policies. Whilst we consider our selection of drugs justifiable and appropriate to focus the story, we would be willing to include this arm in the either the main text or the supplemental information if this is considered essential.

In the current revision, we have clarified our decision in the methods:

“The combination of SP-AQ with an SLD TQ (n=20) was also not considered in the analysis since SP-AQ is currently only considered for seasonal malaria chemoprevention (SMC) and the addition of SLD TQ is not considered for this purpose.”

Methods:
No suggested revisions/comments.

Results: 
L248: The authors state that "[p]rior to treatment, 68.8% (190/422) of individuals were infectious to mosquitoes (Figure 2C)". Figure 2C is a graph of the proportion of mosquitoes infected after feeding on venous blood prior to treatment, and does not comment on the number of individuals involved, so if it is to be cited here the text should be changed to more correctly reflect the Figure, or vice versa.

Response: We have now changed this in the text to refer to supplementary table 2, where the proportion of infection individuals is indicated.

Comments on Figures cited in Results: 

-       Figure 2: The authors describe that 99.5% of patients have gametocytes (L245), however they show in Fig 2C that only 10-15% of mosquitoes become infected. How usual is this as a ratio of infectivity - is this reflective of infection rates in other studies? This should be commented on in the text.

Response: Our findings on pre-treatment transmission potential are similar to other studies assessing infectiousness of individuals with comparable gametocyte densities. For example, in Goncalves 2016 in Burkina Faso (PMID: 26952094), recruiting gametocyte positive individuals, the proportion of infected mosquitoes was 20.0% (IQR 8.0-39.0). Similarly, in Ahmad et al. 2021 in the Gambia (PMID: 34107048), 15.4% of all mosquitoes were infected, after DMFA experiments with blood samples from gametocyte carriers. Lastly, in a meta-analysis of mosquito feeding assays conducted in four countries, 14-20% of mosquitoes became infected when feeding on blood with gametocyte densities of 16-100 gametocytes/µL (PMID: 22936993).

An addition has been made in the text to clarify this:

“…, which is similar to other studies assessing transmission via DMFAs in participants with comparable gametocyte densities (31-33).”

Our findings, as the reviewer indicates, also highlight the clear contrast between gametocyte carriage and actual transmission to mosquitoes. This has been highlighted in previous manuscripts (one actually with the title ‘How important is gametocyte clearance after malaria therapy?’ PMID: 27317420) and was the key reason for our laborious but considerably more informative approach where we assess transmission potential by mosquito feeding assays before and after treatment. This is illustrated in our final figure 6 where the time to achieve transmission blockade is contrasted with the time to gametocyte clearance and is discussed in some detail in the discussion section.

-       Figure 3: The 95% confidence intervals are really very large in the non-8-aminoquinoline graphs and they overlap significantly. While the change in means is indicative, the authors should be cautious in drawing conclusions when there is such variability in results. Presumably this is due to small sample size/underpowering, and this limitation should be emphasised further in the Discussion. As described below (see Discussion comments), in L398-406, how do the authors explain the discrepancy between relative reduction in gametocyte density by microscopy (Fig 3C) and PCR (Fig 3D) in SP-AQ and Non-ACT-PQ graphs, where the density increases by microscopy but decreases by PCR?

Response: This is a valid statement and in the network meta-analysis (NMA) approach proposed by one of the reviewers, confidence intervals have not become smaller.

This has now been added to the limitation section in the discussion:

“.. certain treatment groups included in the analysis had small sample sizes, resulting in wide confidence intervals in the relative reductions and limiting the ability to draw robust conclusions for these groups.’

The transient decrease in relative reduction in gametocyte densities by microscopy in the SP-AQ and Non-ACT-PQ groups has large confidence intervals and is not significant. It therefore does not present a substantial difference compared to the PCR results, given that there is no significant difference in the relative reduction in gametocyte density at day 2 compared to baseline with both methods.

-       Figure 4: Again, as in comments below on L398-406, Figure 4B demonstrated a transient increase (albeit again with large confidence intervals) in the proportion of infected mosquitoes on Day 2 for PY-AS ands AS-AQ. This should be clarified and explained in the text.

Response: As the reviewer states, these transient increases in the proportion of infected mosquitoes in certain arms have very large confidence intervals. After implementing an NMA, as the statistical reviewer suggested, these transient increases have become even smaller and the confidence intervals larger. We therefore have not mentioned these in the text, as they are not statistically robust and do not contribute meaningful insights to the overall interpretation of the data. Instead, we focus on evident (and novel) patterns that highlight considerable variation in time to full prevention of transmission between different ACTs.

-       Figure 6: This is a clearly presented Figure, nicely highlighting and summarising the key finding of the paper.
Response: We thank the reviewer for this positive feedback.


Discussion:
General: The authors do not expound upon their finding of a decrease in gametocytes with AL compared to other ACTs (which is surprising given neither drug is meant to be very efficacious against late-stage gametocytes, as they describe in their Introduction). More emphasis should be given to this in the Discussion, including possible explanations for this finding and a review of literature on the subject. For example, PMID 22363211 describes lumefantrine and the related compound halofantrine inhibiting sporogony but not significantly impacting exflagellation, suggesting that these compounds may act on oocysts. Likewise, PMID 22042867 describes reduced oocyst numbers and activity against early (stage I-III) gametocytes in the presence of 5X IC50 lumefantrine concentrations.

Response: After changing the analysis to a network meta-analysis, as suggested by the statistical reviewer, the differences in gametocyte density reduction between the AL group and other ACTs have become smaller. Nevertheless, the superior transmission-blocking effect of AL remains evident. To help interpret this striking finding, we have extended our discussion section to examine possible mechanisms of transmission-blocking activity of AL:

“AL led to significantly larger reductions in mosquito infection rate on day 2 after initiation of treatment compared to DHA-PPQ, PY-AS, SP-AQ and AS-AQ. Whilst superior gametocyte-clearing effects of AL have previously been demonstrated when compared with DHA-PPQ (40) and PY-AS (41), the reason is not entirely clear since lumefantrine only exhibits an effect on gametocytes and transmission at concentrations higher than therapeutic levels (2,5–7). The total dose of the artemisinin component may be higher in AL compared to DHA-PPQ. In addition, lumefantrine may exert an effect on oocysts by impairing sporogony without significantly affecting male gamete exflagellation (42). Similar observations have been reported in another study, which demonstrated a reduction in oocyst numbers at the lumefantrine IC50 concentration, while effects on early-stage gametocytes were only evident at concentrations five times the IC50 (2).”

We are therefore confident that our findings are robust and agree with the reviewer that the mode of action by which AL exerts this stronger gametocytocidal and transmission-blocking effects remain to be formally demonstrated. That is, for obvious reasons, beyond the scope of a meta-analysis of clinical trial data.

L398-406: The authors discuss the previous literature in terms of whether some drug treatments transiently increase infectivity. The authors comment that their analysis "did not find evidence for post-treatment enhancement of infectivity" but their exploration does not seem to be fully reconciled with their data. Do some graphs (Figures 3C and especially Figure 4D) not suggest a relative increase in gametocyte density by microscopy and proportion of mosquitos infected on Day 2, before reducing by Day 7? Further, how do the authors explain the discrepancy between relative reduction in gametocyte density by microscopy (Fig 3C) and PCR (Fig 3D) in SP-AQ and Non-ACT-PQ graphs, where the density increases by microscopy but decreases by PCR?

Response: We have rephrased this since we agree with the reviewer that a transient increase in transmission potential cannot be ruled out. However, any transient increases in infectivity after treatment we observed were very small and had large confidence intervals, especially after the network meta-analysis (NMA) that has now been conducted.

Similarly, the apparent discrepancy between microscopy and PCR has large confidence intervals. Transient decreases in relative reduction in gametocyte densities by microscopy in the SP-AQ and Non-ACT-PQ groups therefore do not present a substantial difference compared to the PCR results. Neither method shows a statistically significant reduction (or increase) in gametocyte density in these treatment groups at day 2 compared to baseline.

We rephrased our statement that we “did not find evidence for a statistically significant enhancement of infectivity post-treatment” to “Our analysis, almost exclusively examining ACTs, could not rule out a certain degree of post-treatment enhancement of infectivity. Our best estimates indicate a small and transient enhancement in infectivity that was non-significant.”

L430: The authors state that they observed "a strong association between oocyst prevalence and oocyst densities, indicating that a higher proportion of infected mosquitoes reflects a greater transmission potential". While Supplementary Tables 18-23 and Figure 9 describe infection of mosquitoes and then oocyst density, I could not see this association clearly described. A supplementary figure clarifying this would be beneficial for the reader.

Response: This data is being presented in Supplementary Figure 2. This was indicated in the relevant results section, but we inadvertently forgot to reference this figure in the discussion. This oversight has now been corrected, and the text now refers to Supplementary Figure 2 at the end of this sentence. We consider this a relevant addition to the results that are largely based on the proportion infected mosquitoes since there is recent evidence that sporozoite density (and thus oocyst density) is a relevant factor in determining the infectivity of Plasmodium-infected mosquitoes.

L432: The authors state that "this pooled analysis set out to assess the ability of antimalarial treatment to prevent the transmission of drug-resistant parasites." How did they aim to do this? This is not clearly mentioned previously (e.g. in the Introduction or the Methods) and no information on drug resistance (e.g. genotyping) of the mono-infections from the clinical studies is described in this study. I am not sure the study successfully achieved this objective, nor whether it was designed to do so, and therefore this sentence should be clarified.

Response: We agree with the reviewer that our study did not specifically look at this and we have removed reference to drug-resistance in the description of our aims and findings. The use of transmission-blocking drugs is recommended in areas threatened by drug resistance, but our study does not allow a specific assessment of the efficacy of antimalarial drugs to block resistant parasites.

***** Reviewer #2:** 

Vanheer et al conducted a combined analysis of six trials at a single study site using consistent methodologies to compare the gametocytocidal and transmission blocking activities of different antimalarial dosing schedules and regimens.

The authors found substantial differences in gametocytocidal and TB activities of ACTs with AL being more potent than DHA-PPA, AS-AQ and PY-AS. They also observed that SLD improved potency of each ACT and may be beneficial in community treatment campaigns. Assessing and comparing the transmission blocking properties of different antimalarial regimens is an important research question and could inform treatment guidelines.

Whilst this study is using results and existing knowledge from conducted trials, the analysis on these combined studies generates new knowledge that is quite useful and powerful. Results presented in this paper demonstrate the effectiveness of pooled analysis and are informative to malarial treatment programs.

Response: We appreciate these positive considerations of our methodology and the relevance of our work.

As stated by the authors there are a few limitations of the study - sub microscopic gametocytes and their contribution to ongoing transmission post treatment, the use of SMFAs rather than DFAs, and the measurement of oocysts and not sporozoites. However, despite these limitations which are out of the authors control, the authors do not overstate or misrepresent the data presented.

As outlined in the manuscript it would be helpful to extend these pool studies to other malaria endemic locations with different transmission settings and drug-resistant mutations in the future.

Response: We appreciate this comment and one of the reasons for this analysis was to summarize the findings in an area without reported artemisinin, piperaquine or lumefantrine resistance. We are currently undertaking studies in areas where partial resistance to artemisinins has been reported (i.e. Uganda and Ethiopia) with results expected by the end of 2026.

Overall, I think this is a great study that presents important results in a balanced and measured way.

We thank the reviewer for their positive feedback.

**Reviewer #3:** 

*Points of Detail*

1. I am afraid correlation or regression does not measure agreement. (1, 2, 3) is perfectly correlated with (100, 200, 300) but they clearly do not agree. The canonical way to assess and present agreement be- tween two continuous measurement systems is due to Bland and Alt- man (Altman and Bland, 1983; Bland and Altman, 1986). There are several packages on CRAN which implement this and provide the corre- sponding plot. I have used https://cran.r-project.org/package= BlandAltmanLeh which has a very helpful vignette but there are several other options (as the vignette points out).

Response: We appreciate this suggestion. We have now used the Bland-Altman method to better assess agreement between measurement systems, using the BlandAltmanLeh R package. A corresponding Bland-Altman plot has been added to the figures, as per the recommendation. Details on the methods used are described in the Methods section.

2. Page 12: It would be good to have a proper citation for the package used. It is the only credit CRAN authors get. (Disclaimer: I am such an author although not of this package.)

Response: We have added the appropriate citation for the statistical packages to ensure CRAN authors receive proper credit.

3. Figures 3 and 4: The confidence intervals are not clearly visible in these. Perhaps desaturate the colours to correct. In fact the bars are redundant as all the information is contained in the central estimate and the end–points of the interval.

Response: We have made the error bars more visible now. As the confidence interval is not symmetric around the point estimate and it’s impossible to interpolate the point estimate from the confidence intervals. Therefore, we have not removed the bars representing the point estimate.

4. Appendix 2: Putting something on a pre–print server seems to me to fall within the definition of publication.

Response: This paper has now been published, and this has been adjusted in the revised appendix 2.

*Points of More Substance*

5. Combining arms: Concerns raised about combining ACT+PQ arms across different trials, as it compromises randomization benefits and overlooks ACT differences.

Response: We acknowledge the concerns regarding the combination of ACT+PQ arms and recognize the potential biases introduced by aggregating data across trials, however, we believe our approach is valid because of the following:

**1. Scientific and Statistical Rationale for Combining Arms**
The decision to combine treatment arms was driven by the specific characteristics of our data and study objectives:

- **Focus on Within-Arm Reductions**: Our primary interest is in reductions from baseline (day 0) for each treatment. This choice ensures that each treatment arm acts as its own control, reducing the influence of between-arm differences at baseline. By analyzing reductions within arms and comparing these reductions between treatments, the need to preserve strict randomization across arms is mitigated.
- **Statistical Efficiency**: Combining arms was necessary to improve statistical power and reduce variability in effect estimates. Treating each arm separately in the presence of sparse data in a full interaction model can lead to unstable estimates with wide confidence intervals. Combining arms with clinically and statistically similar profiles helps address this issue and provides more reliable results.

**2. Sensitivity Analyses to Assess Robustness**
To address concerns about the impact of combining arms, we conducted additional analyses by including all treatment arms without combining any, allowing for a direct comparison of composite versus disaggregated arms.

These sensitivity analyses, detailed in the supplementary materials, confirm that the results of the primary analysis are consistent with those observed in the disaggregated analyses. The composite arms exhibit similar patterns to the distinct-arm analyses, suggesting that combining arms does not materially alter the interpretation of results.

**3.** **Minimizing Potential Bias**
We recognize the statistical concern that combining arms might disrupt randomization. However, this concern is reduced in our analysis by the focus on reductions from baseline, which inherently controls for between-arm variability. Additionally:

- The arms combined in our analysis are clinically coherent and aligned in their mechanisms of action, treatment regimens, and baseline characteristics.
- The robustness of our findings across sensitivity analyses provides reassurance that combining arms has not introduced systematic bias.

**4.**  **Network Meta-Analysis as a Complementary Approach**
While our initial analysis did not employ a network meta-analysis (NMA), the NMA allows for further justification of creating combined arms. Combining arms strengthens the connections in the treatment network, thereby providing more precise estimates of indirect treatment effects. Sparse or disconnected nodes in the network can result in unstable or non-identifiable comparisons, whereas combined arms contribute to the network's overall stability. For example, for relative reductions in oocyst density, studies NECTAR1 and NECTAR2 had no overlapping treatments with NECTAR3 and NECTAR4, hence indirect effects for those treatment arms could not be computed.

6. Direct and indirect comparison: Highlights need to recognize and handle indirect comparisons separately.

We have now implemented a NMA to combine direct and indirect effects of treatment arms across studies. To apply the NMA, we ran the regression models for each study and extracted the treatment effects and standard errors. Thereafter, a random effects NMA was applied. For sensitivity, we also applied the NMA to all treatment arms, which is presented in supplementary figures 3-5, 8 and 10. The R package netmeta was used for the NMA analysis and now mentioned in the Methods section.

**Reviewer 1 Rapid Reviews/Infectious Diseases**

**Dr. Leah Moriarty:**

The study is reliable. The main study claims are generally justified by its methods and data. The results and conclusions are likely to be similar to the hypothetical ideal study. There are some minor caveats or limitations, but they would/do not change the major claims of the study. The study provides sufficient strength of evidence on its own that its main claims should be considered actionable, with some room for future revision

Researchers examined data from 6 repeat studies to examine the transmission-blocking activities of several antimalarial medications in Mali. This is an important study in light of recent evidence of partial artemisinin resistance and low partner drug efficacy in Africa. National Malaria Programs should consider the transmission blocking effect on treatment regimens to slow the spread of resistant parasites in setting antimalarial drug policy. This well-written and described paper provides important evidence to inform these policies.

Response: we appreciate this positive feedback.

Methods. There is no mention of sample size for this study- understanding this is a secondary analysis so the sample size wasn't likely powered for this study, but it is still important to contextualize the estimations being made.

Response: we have included a description of the sample size of individual studies. These were based on an online tool to calculate study power (presented in PMID: 35720362) and all individual study arms in the individual studies had >80% power to detect a reduction in transmission of >90% on either day 2 (studies with primaquine, studies with artemether-lumefantrine) or day 7 (other study arms).

Results

Line 263: Suggest to remove "depended strongly" and replace with a quantified measure

Response: we updated this statement. We removed ‘strongly’ and indicate the quantitative estimates of gametocyte prevalence of density reductions in the sentences following this updated statement.

The results section could use some sub-headers. Authors may also consider re-arranging to organize the results by treatment instead of outcome. As it is currently written it is hard to follow.

Response: we appreciate this comment and have added sub-headers to improve the structure of our Results section.

Discussion

Line 440- not just artemisinin-resistant malaria infections but also infections less susceptible to lumefantrine, which is the case in several countries in Africa.

Response: we agree that susceptibility to lumefantrine is a major concern. Whilst there currently is no confirmed marker for lumefantrine resistance, there are indeed several reports that indicate increased IC50 values for Lumefantrine. We added a sentence and reference (PMID: 34553183) to highlight indications for changes in lumefantrine susceptibility that may affect the efficacy of ACTs.

This reviewer recommends adding small sample size as a potential limitation- there is a lot of statistical inference here with a relatively small number of patients.

Response: The second reviewer also comments on the sample size of the original studies but in a positive way, she indicates that the power has improved due to our combination of individual studies. In addition to mentioning the sample size considerations of the original studies in the Methods section, we highlight in the Discussion section that the sample size of original studies was modest but studies were designed to have appropriate power to quantify the transmission blocking activity of medication in a population who was highly infectious prior to treatment (PMID: 35720362).

**Reviewer 2: Dr. Patricia Graves**

This study is reliable. The main study claims are generally justified by its methods and data. The results and conclusions are likely to be similar to the hypothetical ideal study. There are some minor caveats or limitations, but they would/do not change the major claims of the study. The study provides sufficient strength of evidence on its own that its main claims should be considered actionable, with some room for future revision.

***************************************

Review: It is interesting and useful to combine the recent trials of the effect of different ACTs on gametocyte prevalence and density, proportion of infected mosquitoes and mean oocyst density. The number of participants per study was relatively low, so combining them improves the power of the findings. The paper has been carefully compiled with explanations of the similarities and differences between the trials, with useful graphics such as Figure 1 to show when outcomes were assessed. The trials were done in similar fashion with consistent methods for assessing outcomes such as infectivity.  In fact all the graphics used are very clear and show the differences between the ACTs.  There are apparently a large number of supplementary files which I wanted to consult but could not find a link to.

Response: We appreciate the appreciation of the analysis and the graphics. For some reason the supplemental information was not uploaded on MedRxiv, uploading occurred to the journal PLoS Medicine. We have now uploaded the supplemental information manually and it is now available.

The results of the comparisons between DHA-PPQ, SP-AQ, PY-AS, AS-AQ and AL are all clear, especially in Fig 6. I don't find it valid to combine all the Non-ACT-PQ and compare with ACT-PQ.  Non-ACT-PQ includes only SP-AQ with PQ (N=20) if I am not mistaken (Table 1).  ACT-PQ includes AL, AL-AQ, DHA-PPQ, AS-AQ and PY-AS with high heterogeneity between their effects (without PQ) and many more participants (N=142). I don't think that you can conclude from this that PQ should be added to 'ACT'. The statement in lines 364 to 366 that "adding a single low dose of PQ ...to any ACT accelerated the clearance of gametocytes and led to significantly greater reduction in mosquito infection rate..." needs more statistical justification. It likely depends on the ACT, but without access to the data on separate ACTs with and without PQ in valid comparisons I cannot assess.  Similarly calling the last graph in Fig 6 "ACT-TQ" is a bit misleading when it is only DHA-PPQ in this group.

Response: We appreciate that some of the data of individual study arms were not visible in the original submission. Because all ACTs show highly similar/identical rapid reductions in transmission potential, we feel it is justifiable to combine them. We do, however, agree that it is valuable for the reader to see this evidence in more detail. In the revised manuscript, we have thus provided data of the individual study arms in the supplemental information.

We have added a clarification in the discussion to highlight that TQ was only tested in combination with DHA-PPQ (line 667): “It is important to note that TQ was evaluated only in combination with a single ACT, DHA-PPQ, in these studies, whereas PQ was assessed in combination with multiple ACTs, including DHA-PPQ, AL, PY-AS, and AS-AQ.”

There is so much valuable information in this paper, much of which is apparently in supplementary files. I think it could be split into two or more papers, with the first main findings comparing the ACTs, and the PQ and TQ addition in a separate analysis.  This would allow inclusion of more of the relevant data in the main text, such as the mosquito infection data (lines 296 to 298).

Response: we believe that with the improved and better structured Results section and the extensive Supplemental information, the current manuscript does justice to the wealth of underlying data and has the advantage of having a single paper that compares the transmission-blocking properties of all currently considered malaria treatments that have available data on this outcome.
