# Supplementary material for: The transmission blocking activity of artemisinin-combination, non-artemisinin, and 8-aminoquinoline antimalarial therapies: A pooled analysis of individual participant data
Source: PLoS Med. 2025 Aug 14;22(8):e1004683. doi: 10.1371/journal.pmed.1004683 (PMC12352847; doi:10.1371/journal.pmed.1004683)
Supplement: Attachment — Submitted filename: 090625_Response_reviewers_revision.docx [file pmed.1004683.s007.docx]

**Summarized comments from the academic editor:** 
1. The studies included in this analysis were conducted for purposes other than future metanalysis. If a comparison of all study arms were intended the design of the studies would have been different. The authors are reusing study data for a purpose other than answering the original research questions. Please acknowledge this inherent limitation of data re-use.

Response: We agree and at the same time it is evident that very few meta-analyses are done on studies that were designed to become part of a meta-analysis. The near identical enrolment criteria and identical methodology in our studies actually make the studies very suitable for a meta-analysis. We have now added this to the study limitations section in the discussion.

*“Secondly, this analysis reuses data from studies that were highly similar in enrolment criteria and methodology but not originally designed for the conduction of a pooled analysis.”*

2. In general, the reporting of the study limitations should be expanded.  For example, the enrollment in some studies is a decade apart. Considering that the susceptibility of parasites to antimalarials is changing over time, and changes in the susceptibility of gametocytes precedes other markers of resistance, the authors are not comparing same with same. This should be acknowledged.

Response: We agree with the editor that the long time period could be mentioned and have now added this to the study limitations section in the discussion:

*“Furthermore, the studies included in this analysis were conducted over the course of a decade, and susceptibility of parasites to antimalarials may have changed over time.”*
 
3. A reduction in infectivity is not the same as a reduction in transmission. To my knowledge no empirical evidence that such a reduction in infectivity equals a reduction in malaria transmission. It is not difficult to imagine a situation in which most members of a population carry subclinical infections and continue to infect mosquitoes thus contributing to transmission. Individuals treated for clinical malaria make up only a small fraction of the overall population. Treating such a small sub-population will probably make no meaningful difference in the overall transmission prevalence of infections, morbidity, or mortality. This should be acknowledged.

Response: We have now added an acknowledgement of this to the discussion:

*“While reductions in transmission potential among treated individuals are encouraging, they may not directly translate to reductions in overall malaria transmission in communities and this depends on the populations in which antimalarials are deployed. Asymptomatic and untreated infections likely contribute substantially to ongoing transmission in many settings. Such infections may be targeted through mass drug administration with non-artemisinin-based treatments such as SP-AQ, for example in the context of SMC; our data suggest that adding a gametocytocide to this combination could provide additional benefits.”*

4. The authors state, ”Our data on SLD gametocytocides in combination with ACTs therefore supports the 2023 advice from the WHO malaria policy and advisory group to expand the focus on reducing parasite transmission with SLD PQ in areas where partial artemisinin resistance has been detected (20).” The authors need to acknowledge the need to support the expert advice with evidence. Moreover, if one or more of the authors were part of the WHO expert group that provided this advice, or participated in a WHO malaria policy and advisory group, the authors should declare potential for conflicting interests to ensure transparency and accountability.

Response: We have now added to the Competing Interests section that author CD is a member of the Malaria Policy Advisory Group (MPAG). TB contributed to a Guideline Development Group of the World Health Organization for recommendations for the final phase of elimination and prevention of re-establishment of malaria.

No controlled trial exists comparing evolution of drug resistance in areas where SLD PQ is used compared to areas where SLD PQ is not used. Whilst gametocytes are the only parasite blood stages capable of transmitting, and targeting and inhibiting these stages is likely to reduce the spread of drug resistant parasites, this has not been formally demonstrated. Here, one may argue that the threat of drug resistance is sufficiently large to warrant the use of PQ even in the absence of further evidence. We added a few words to indicate limited evidence in the Discussion section:

*“Of note, evidence is needed to support this expert advice on the utility of SLD PQ.”*

**Reviewer #1:** This is an excellent revision by Dr. Bousema and colleagues. I was the first reviewer and had quite detailed comments that were fully addressed. In response to the statistical reviewer, the authors now include a network meta-analysis that has helped strengthen the statistical analysis while not changing core findings from the comparative analysis across the six clinical data sets (five of which have been published as peer-reviewed articles with the sixth being on a preprint archive). 

The study contains a wealth of important information about the effects of the different artemisinin-based combination therapies (ACTs) on Plasmodium falciparum transmissibility. The findings of superior transmission-blocking activity of AL (artemether-lumefantrine) compared with other ACTs, and the analysis of transmission-blocking by single low dose 8-aminoaquinolines, are very valuable in the context of current discussions about the need to diversity ACTs across Africa to reduce selective pressure on lumefantrine. This is especially relevant given the increasing number of African countries where artemisinin partial resistance has been identified.  

Overall, I find this to be a very high quality and timely addition to the literature that will generate considerable interest in the malaria research and treatment and control communities.

Response: We thank the reviewer for this positive feedback.

**Reviewer #2**

Points of detail

Page 10 Choosing not to evaluate arms which do not correspond to current practice risks missing some novel effect. Conventional wisdom is not always right. I think this is more an issue for the clinical reviewers. If they agree then omitting those arms is fine.

Response: These are valid concerns, but we feel that not evaluating these arms is the correct decision because they are not currently considered as therapeutic treatments. In addition, they were already examined in the original clinical trials and therefore are not likely to show any surprising effects in this pooled analysis. The clinical reviewer has no objections. Of note, we share all available data online to allow researchers to conduct their own analyses if they wish.

Page 13 and Figures 2A and B I do not understand why some of these are truncated below and indeed in 2A why the first one is truncated above.

Response: The reviewer makes a valid observation that our data in Figure 2A and B appears truncated. However, the apparent truncation in Figure 2A and 2B reflects features of the data collection process rather than graphical omission. In Figure 2A, the lower boundaries of the asexual parasite density distribution appear flat because parasite counts were based on 200 white blood cells; thus, the lowest detectable value corresponds to 1 parasite per 200 WBCs—equating to 40 parasites/µL (assuming 8000 WBCs/µL)—with a wide dynamic range above that threshold. The 2013–2014 density curve appears flat and abruptly truncated at the top due to the nearly uniform distribution of the log-transformed data, with observed points sparsely spread across the full range. For Figure 2B, the lower end of the gametocyte density distribution is shaped by study-specific inclusion criteria: a minimum of approximately 16 gametocytes/µL in 2013–2014 and 32 gametocytes/µL in all subsequent studies. Moreover, gametocyte densities were derived from microscopy counts over a fixed number of white blood cells, resulting in multiplication factors that ‘bin’ values and produce apparent steps or gaps at the lower end of the distribution. These effects are visual artefacts of biological thresholds and quantification methodology rather than imposed truncation.

Since the impact of these factors on the graphical presentation was not immediately clear, we have added a sentence to the figure legend to clarify the apparent truncation:

*“Apparent truncations in panels A and B reflect characteristics of the data collection process rather than omissions. These arise from (i) inclusion criteria, and (ii) parasite quantification based on reading a fixed fraction of a microliter, which introduces a multiplication factor and results in 'binning' of similar parasite densities.”*

Page 13 and Figure 2C I find it hard to see what I am being told here. If this is just presenting proportion infected per participant why was a logistic regression needed? Given the numbers of participants per trial why not just plot the proportions per participant?

Response: In Figure 2C, we used a logistic regression model rather than directly plotting individual-level proportions to account for heterogeneity in the number of mosquitoes dissected per participant. The proportion of infected mosquitoes per individual carries uncertainty that depends on the number of mosquitoes tested; for example, infecting 2 out of 5 mosquitoes provides less certainty than 20 out of 50. By using a binomial logistic model with participant-level random effects, we could appropriately account for this variation in precision across individuals and avoid overinterpreting noisy estimates based on small sample sizes. Averaging raw proportions without accounting for this uncertainty would give disproportionate weight to participants with very few mosquitoes dissected. The model therefore provides more stable and reliable summary estimates across time periods. Although these features of transmission data will be familiar to researchers in this subject area, the availability of all of our data online allows anyone to see the raw mosquito sample size and infection prevalence data, if they wish.

Page 14 and Figure 3A I agree this shows excellent agreement between the two methods.

Page 14 ‘[. . . ] could be observed in the ACT–PQ group only at seven days [...]’ is ambiguous. Does it mean that was the only time that group achieved statistical significance or does it mean that was the only group that achieved statistical significance at that time point. The English word ‘only’ is a slippery customer. Perhaps re–write the sentence?

Response: this paragraph has been re-written given the revision in methodology.

Page 14 and Figures 3B to D Are the authors trying, by comparing above and below some level of statistical significance, to imply that there is a difference? If so that is not the way to do it (Gelman and Stern, 2006).

Response: We appreciate the reviewer’s comment and agree that statistical significance alone should not be used to imply differences between groups (Gelman & Stern, 2006). In our revised interpretation of Figures 3B–D, we have moved away from dichotomising results based on statistical significance and instead focus on reporting and interpreting the magnitude and uncertainty (95% confidence intervals) of within-arm reductions. The intent of these figures is not to make between-arm comparisons, but to illustrate the trajectory of reductions from baseline within each treatment group over time.

Page 14 and Figures 3B to D I do not understand the comment in the caption about truncating the y–axis. Why were the standard errors inflated? This must tell us something about either the data or the model.

Response: In Figures 3B–D, the reductions in gametocyte prevalence and density are expressed as relative changes from baseline, which, by definition, can theoretically range from 100% to –∞. In cases where reductions are close to zero or where measurement uncertainty is high, confidence intervals can extend far into negative values. For visual clarity, we truncated the y-axis at –50% in these figures. This does not affect the underlying estimates or inference but helps focus the reader’s attention on the main comparison range. We also note that reductions at day 2 are accompanied by greater uncertainty. This is expected, as the biological effects of most treatments on gametocyte clearance are likely delayed, leading to substantial inter-individual variability early on. Additionally, for gametocyte prevalence (a binary outcome), we acknowledge that reduced statistical power and broader confidence intervals are to be expected due to the dichotomous nature of the measurement.

We have added a sentence to the figure legend to acknowledge this:

*“The y-axis was cut off below -50 due to inflated standard errors, as a result of reductions from baseline close to zero or high measurement uncertainty”*

Page 16 and Figure 4B I do not see the PY–AS group failing to show a difference at day 14 as the confidence interval does not include 0.

Response: this section has been revised.

Pages 16 and 17 Is this supposed to be a report of the NMA? I would make that explicit. In Figure 5 it would be much better to have the effect size in the cells rather than a p–value. Are these the P–scores produced by the netrank function in netmeta perhaps?

Response: Figure 5 has now been revised to include the NMA network in A and the p-scores in B. The heatmaps in C and D now show the effect size, CI and p-values in the cells.

Page 19 The NMA is discussed here but it should have been presented in the results section.

Response: The NMA results have been added to the results section now.

*“Validity of the network meta-analysis*

*The NMA assumption of transitivity (see supplemental Methods 1, appendix 1, p 12) was supported by the general similarity in participant characteristics and pre-treatment transmission potential across the included studies. Importantly, for longitudinal outcomes, the primary outcomes were defined as relative reductions from baseline within each individual, which further mitigates concerns related to baseline heterogeneity across studies. These factors support transitivity across the network of treatment comparisons. Visual inspection of the consistency showed that direct and indirect estimates were generally in agreement, with overlapping confidence intervals (supplementary figure 21, appendix 1, p 43). This suggests that the consistency assumption was reasonably upheld across the treatment networks included in this analysis. In general, the network of treatment comparisons was relatively sparse, with most treatment pairs compared in only one or two studies (Figure 5A). This limited connectivity likely contributed to the wide confidence intervals observed for some indirect comparisons, particularly where few or no direct comparisons were available.”*

Point of more substance

As it stands the paper does not seem to have taken full advantage of the NMA unless I have missed something. It is discussed but without any attempt at presenting it fully. Since comparing the treatment regimes and recommending a best buy seems a goal I would have thought the analysis known in the Bayesain community as SUCRA was almost mandatory. A frequentist analogue is provided in netmeta.

In addition for an example of the sort of plots I would have expected to see one might look at Barth et al. (2013). The relevant PRISMA guidelines (Hutton et al., 2015) are also relevant.

Response: Thank you for your thoughtful comment. We fully agree that network meta-analysis (NMA) should be leveraged to its full potential, and we have made substantial updates to the analysis and presentation in the revised manuscript to ensure this. In the original submission, we had implemented a single-stage NMA using raw outcome values across all timepoints. However, this approach had several limitations: (i) it resulted in overly complex treatment networks due to the inclusion of all arm-by-time combinations (e.g., ACT-PQ day 0 vs. AL day 7), making network structure, forest plots, consistency assessments, and treatment rankings difficult to interpret; and (ii) it generated potentially misleading significance for comparisons with negligible clinical differences (e.g., 97% vs. 99% reduction), due to the exponential transformation of large β estimates with small standard errors when calculating relative reductions, i.e. RR = 1-exp(\beta).

In response to this and other reviewer feedback, and after consultation with meta-analysis expert Thomas Debray, we have adopted a more interpretable and rigorous two-stage NMA approach. In the first stage, we modelled individual-level data to estimate the reduction from baseline for each treatment arm and timepoint relative to baseline (day 0) for each study. We then calculated all pairwise differences in reductions (and their standard errors, using the delta method), which formed the input to separate NMAs at each timepoint. This allowed us to use network plots, consistency assessments, forest plots, and treatment rankings (via P-scores, the frequentist analogue of SUCRA) in a transparent and clinically interpretable way.

These updates now directly address the points raised in PRISMA-NMA guidelines (Hutton et al., 2015), and we believe this current framework more appropriately supports comparisons across treatments and identification of optimal regimens. The revised manuscript now includes a network diagram, as well as forest plots visualising comparisons with the reference arm ACT-PQ for all each outcome and timepoint. P-score based treatment ranking and consistency assessment, in line with the expectations outlined in Barth et al. (2013), are included for the proportion infected mosquitoes, as this is the endpoint with the largest public health relevance.

In a separate file, we have provided the consistency assessment figures and P-score plots for all outcomes that were considered in this analysis.

The methods section has been revised to include these substantial updates to the methodology.

In addition to the paragraph added to the results section, a supplementary methods section has been added, as well as an updated discussion of the NMA:

*“The use of network meta-analysis in this pooled analysis enabled comparisons across a broad set of treatment regimens, including many that were not directly evaluated against each other within individual trials. By estimating reductions from baseline within each treatment arm and comparing these reductions across arms, we synthesized both direct and indirect evidence for how the different treatment regimens compare in terms of their gametocytocidal and transmission-blocking activities. While this approach allowed for a more comprehensive assessment of relative efficacy, the network was relatively sparse, with most comparisons informed by only one or two studies. This contributed to wide confidence intervals for some contrasts and added uncertainty to the treatment rankings. These limitations highlight the impact of network sparsity and reinforce the importance of both network structure and statistical precision when interpreting relative treatment rankings. Nonetheless, the consistency observed between direct and indirect estimates, combined with standardized trial procedures, supports the validity of the network-based comparisons.”*

**Journal Requirements:**

1)  Please ensure that the funders and grant numbers match between the Financial Disclosure field and the Funding Information tab in your submission form. Note that the funders must be provided in the same order in both places as well.
 Currently, (Jordache Ramjith and Teun Bousema by a VICI fellowship from the Dutch Research Council (NWO; grant number 09150182210039))is missing from the Funding Information.

We have now ensured that the information presented in the Financial Disclosure field and Funding Information tab are the same.

2) Please ensure your short title is correct in the submission form, including symbols, italicisation etc. Your short title will be typeset from the submission form directly.

We confirm that the short title is correct:

“Transmission blocking activity of antimalarial regimens: a pooled analysis of individual participant data.”

3) Please provide separate figure files in .tif or .eps format.

For more information about figure files please see our guidelines:
 
[https://journals.plos.org/plosmedicine/s/figures](https://url.uk.m.mimecastprotect.com/s/VaLlC8EVLUknzPNCnfxcylfJR?domain=journals.plos.org) 

[https://journals.plos.org/plosmedicine/s/figures#loc-file-requirements](https://url.uk.m.mimecastprotect.com/s/LWvoC9gWMIOoMN4UEhPcqMQnt?domain=journals.plos.org)

The figures are now provided in separate .tif files.

4) We have noticed that you have uploaded Supporting Information files, but you have not included a list of legends. Please add a full list of legends for your Supporting Information files after the references list.

A list of legends for the Supporting Information is now included after the references.

5) Some material included in your submission may be copyrighted. According to PLOS’s copyright policy, authors who use figures or other material (e.g., graphics, clipart, maps) from another author or copyright holder must demonstrate or obtain permission to publish this material under the Creative Commons Attribution 4.0 International (CC BY 4.0) License used by PLOS journals. Please closely review the details of PLOS’s copyright requirements here: PLOS Licenses and Copyright. If you need to request permissions from a copyright holder, you may use PLOS's Copyright Content Permission form.

Please respond directly to this email and provide any known details concerning your material's license terms and permissions required for reuse, even if you have not yet obtained copyright permissions or are unsure of your material's copyright compatibility. Once you have responded and addressed all other outstanding technical requirements, you may resubmit your manuscript within Editorial Manager.

Potential Copyright Issues:

1) "[Figure 1]: Please confirm whether you drew the images / clip-art within the figure panels by hand. If you did not draw the images, please provide (a) a link to the source of the images or icons and their license / terms of use; or (b) written permission from the copyright holder to publish the images or icons under our CC BY 4.0 license. Alternatively, you may replace the images with open source alternatives. See these open source resources you may use to replace images / clip-art:
- [https://commons.wikimedia.org](https://url.uk.m.mimecastprotect.com/s/3_VNC0LKzCy6MgNt2i2c9VCF1?domain=commons.wikimedia.org)
- [https://openclipart.org/"](https://url.uk.m.mimecastprotect.com/s/diwOCg204iBVmwEU3s4c4chY9?domain=openclipart.org)

The mosquito image has now been replaced with an image from <https://openclipart.org/detail/233084/mosquito>.

**General editorial requests:**
(Note: not all will apply to your paper, but please check each item carefully)

*       We ask every co-author listed on the manuscript to fill in a contributing author statement, making sure to declare all competing interests. If any of the co-authors have not filled in the statement, we will remind them to do so when the paper is revised. If all statements are not completed in a timely fashion this could hold up the re-review process. If new competing interests are declared later in the revision process, this may also hold up the submission. Should there be a problem getting one of your co-authors to fill in a statement we will be in contact. Please do not add or remove authors without first discussing this with the handling editor. You can see our competing interests policy here: [http://journals.plos.org/plosmedicine/s/competing-interests](http://url.uk.m.mimecastprotect.com/s/7pXtCk714Tw1vwRhQiBcGMWcT?domain=journals.plos.org).

We are currently compiling forms for all authors and understand that these can be submitted after resubmission of this manuscript. Please let us know if this is not the case.

*       Please upload any figures associated with your paper as individual TIF or EPS files with 300dpi resolution at resubmission; please read our figure guidelines for more information on our requirements: [http://journals.plos.org/plosmedicine/s/figures](http://url.uk.m.mimecastprotect.com/s/m_bLCl714TMplM3Fqs6cz8XSr?domain=journals.plos.org). While revising your submission, please upload your figure files to the PACE digital diagnostic tool, [https://pacev2.apexcovantage.com/](https://url.uk.m.mimecastprotect.com/s/y5caCmy54uMDqM7FQtZcRkjwe?domain=pacev2.apexcovantage.com). PACE helps ensure that figures meet PLOS requirements. To use PACE, you must first register as a user. Then, login and navigate to the UPLOAD tab, where you will find detailed instructions on how to use the tool. If you encounter any issues or have any questions when using PACE, please email us at [PLOSMedicine@plos.org](mailto:PLOSMedicine@plos.org).

The figures are now provided in separate .tif files with high resolution.

*       Please ensure that the paper adheres to the PLOS Data Availability Policy (see [http://journals.plos.org/plosmedicine/s/data-availability)](http://url.uk.m.mimecastprotect.com/s/OL0wCnO14cWjVW1FEugcJ-YNz?domain=journals.plos.org), which requires that all data underlying the study's findings be provided in a repository or as Supporting Information. For data residing with a third party, authors are required to provide instructions with contact information (web or email address) for obtaining the data. Please note that a study author cannot be the contact person for the data. PLOS journals do not allow statements supported by "data not shown" or "unpublished results." For such statements, authors must provide supporting data or cite public sources that include it.

Trial data for NECTAR1-4 studies are available through a novel open-access clinical epidemiology database resource, ClinEpiDB (https://clinepidb.org); The selection of data used for this analysis can be found on https://github.com/leenvh/NECTAR-pooled-analysis.

*       We expect all researchers with submissions to PLOS in which author-generated code underpins the findings in the manuscript to make all author-generated code available without restrictions upon publication of the work. In cases where code is central to the manuscript, we may require the code to be made available as a condition of publication. Authors are responsible for ensuring that the code is reusable and well documented. Please make any custom code available, either as part of your data deposition or as a supplementary file. Please add a sentence to your data availability statement regarding any code used in the study, e.g. "The code used in the analysis is available from Github [URL] and archived in Zenodo [DOI link]" Please review our guidelines at [https://journals.plos.org/plosmedicine/s/materials-software-and-code-sharing](https://url.uk.m.mimecastprotect.com/s/7qBGCoy14uwZmw1hWCncp2Vi1?domain=journals.plos.org) and ensure that your code is shared in a way that follows best practice and facilitates reproducibility and reuse. Because Github depositions can
be readily changed or deleted, we encourage you to make a permanent DOI'd copy (e.g. in Zenodo) and provide the URL. 

All author-generated code used for the analysis in this manuscript can be found on https://github.com/leenvh/NECTAR-pooled-analysis.

*       Please ensure that the study is reported according to the appropriate guideline and include the relevant completed checklist as Supporting Information. When completing the checklist, please use section and paragraph numbers, rather than page numbers. Please add the following statement, or similar, to the Methods: "This study is reported as per [XXXX] guideline (S1 Checklist)."

FORMATTING - GENERAL
*       Abstract: Please structure your abstract using the PLOS Medicine headings (Background, Methods and Findings, Conclusions). Please combine the Methods and Findings sections into one section.
We have reviewed our Abstract and believe it aligns with the specified structure.

*       At this stage, we ask that you include a short, non-technical Author Summary of your research to make findings accessible to a wide audience that includes both scientists and non-scientists. The Author Summary should immediately follow the Abstract in your revised manuscript. This text is subject to editorial change and should be distinct from the scientific abstract. Ideally each sub-heading should contain 2-3 single sentence, concise bullet points containing the most salient points from your study. In the final bullet point of 'What Do These Findings Mean?', please include the main limitations of the study in non-technical language. Please see our author guidelines for more information: [https://journals.plos.org/plosmedicine/s/revising-your-manuscript#loc-author-summary](https://url.uk.m.mimecastprotect.com/s/rDFmCp215iwKZwJhkF9cGpPY0?domain=journals.plos.org).

A non-technical Author Summary has now been added to the manuscript.

*       Please express the main results with 95% CIs as well as p values. When reporting p values please report as p<0.001 and where higher as the exact p value p=0.002, for example. Throughout, suggest reporting statistical information as follows to improve clarity for the reader "22% (95% CI [13%,28%]; p</=)". Please be sure to define all numerical values at first use.

We have reviewed our results reporting and believe it aligns with the specified criteria.

*       Please include page numbers and line numbers in the manuscript file. Use continuous line numbers (do not restart the numbering on each page). 
We have inluced page numbers and line numbers.

*       Please cite the reference numbers in square brackets. Citations should precede punctuation.
We have updated the reference style to square brackets.

FIGURES AND TABLES
*       Please provide titles and legends for all figures and tables (including those in Supporting Information files).

*       Please define all abbreviations used in each figure/table (including those in Supporting Information files).

*       Please consider avoiding the use of red and green in order to make your figure more accessible to those with color blindness.

We have reviewed our figures and tables and believe they align with the specified criteria. Legends for all figures, including supporting information files, are now included in the manuscript.

SUPPLEMENTARY MATERIAL
*       Please note that supplementary material will be posted as supplied by the authors. Therefore, please amend it according to the relevant comments outlined here.

*       Please cite your Supporting Information as outlined here: [https://journals.plos.org/plosmedicine/s/supporting-information](https://url.uk.m.mimecastprotect.com/s/YD7YCqZ14t2lv2xhMHMcE3csb?domain=journals.plos.org)

We have reviewed our supplementary materials and believe they align with the specified criteria.

REFERENCES
*       PLOS uses the numbered citation (citation-sequence) method and first six authors, et al.

*       Please ensure that journal name abbreviations match those found in the National Center for Biotechnology Information (NCBI) databases ([http://www.ncbi.nlm.nih.gov/nlmcatalog/journals](http://url.uk.m.mimecastprotect.com/s/pUgfCr914SmoOmzcxIBc4nVM8?domain=ncbi.nlm.nih.gov)), and are appropriately formatted and capitalised.

*       Where website addresses are cited, please include the complete URL and specify the date of access (e.g. [accessed: 12/06/2023]).

*       Please also see [https://journals.plos.org/plosmedicine/s/submission-guidelines#loc-references](https://url.uk.m.mimecastprotect.com/s/PBwPCvZ14tlKZlPIWSncQC2F7?domain=journals.plos.org) for further details on reference formatting.

We have reviewed our references and believe they align with the specified criteria.

*       For all observational studies, in the manuscript text, please indicate: (1) the specific hypotheses you intended to test, (2) the analytical methods by which you planned to test them, (3) the analyses you actually performed, and (4) when reported analyses differ from those that were planned, transparent explanations for differences that affect the reliability of the study's results. If a reported analysis was performed based on an interesting but unanticipated pattern in the data, please be clear that the analysis was data driven.
*       Please state in the Methods section whether the study had a prospective protocol or analysis plan. If a prospective analysis plan (from your funding proposal, IRB or other ethics committee submission, study protocol, or other planning document written before analyzing the data) was used in designing the study, please include the relevant document(s) with your revised manuscript as a Supporting Information file to be published alongside your study and cite it in the Methods section. A legend for this file should be included at the end of your manuscript. If no such document exists, please make sure that the Methods section transparently describes when analyses were planned, and when/why any data-driven changes to analyses took place. Changes in the analysis, including those made in response to peer review comments, should be identified as such in the Methods section of the paper, with rationale.


MODELLING STUDIES

The following list is derived from Geoffrey P Garnett, Simon Cousens, Timothy B Hallett, Richard Steketee, Neff Walker. Mathematical models in the evaluation of health programmes. (2011) Lancet DOI:10.1016/S0140-6736(10)61505-X:    
                
*       If pertinent, please provide a diagram that shows the model structure, including how the natural history of the disease is represented, the process and determinants of disease acquisition, and how the putative intervention could affect the system.

*       Please provide a complete list of model parameters, including clear and precise descriptions of the meaning of each parameter, together with the values or ranges for each, with justification or the primary source cited and important caveats about the use of these values noted.

*       Please provide a clear statement about how the model was fitted to the data, including goodness-of-fit measure, the numerical algorithm used, which parameter varied, constraints imposed on parameter values, and starting conditions.

*       For uncertainty analyses, please state the sources of uncertainties quantified and not quantified [can include parameter, data, and model structure].

*       Please provide sensitivity analyses to identify which parameter values are most important in the model. Uncertainty estimates seek to derive a range of credible results on the basis of an exploration of the range of reasonable parameter values. The choice of method should be presented and justified.

*       Please discuss the scientific rationale for the choice of model structure and identify points where this choice could influence conclusions drawn. Please also describe the strength of the scientific basis underlying the key model assumptions.

*       For studies that develop a prediction model or evaluate its performance, please ensure that the study is reported according to the TRIPOD statement ([https://www.equator-network.org/reporting-guidelines/tripod-statement](https://url.uk.m.mimecastprotect.com/s/H1RcCwr14SN9nN4t4T7cJl8Xe?domain=equator-network.org)) and include the completed checklist as Supporting Information. Please add the following statement, or similar, to the Methods: "This study is reported as per the Transparent Reporting of a Multivariable Prediction Model for Individual Prognosis Or Diagnosis (TRIPOD) statement (S1 Checklist)." For studies using machine learning, please use the TRIPOD-AI checklist. When completing the checklist, please use section and paragraph numbers, rather than page numbers.

We have reviewed our explanation of the statistical analysis and believe they align with the specified criteria. We did not use a mathematical model or a prediction model. The statistical analysis is clearly explained in the methodology and the network showing direct effects is also visualized in Fig 5.
