# Supplementary material for: The transmission blocking activity of artemisinin-combination, non-artemisinin, and 8-aminoquinoline antimalarial therapies: A pooled analysis of individual participant data
Source: PLoS Med. 2025 Aug 14;22(8):e1004683. doi: 10.1371/journal.pmed.1004683 (PMC12352847; doi:10.1371/journal.pmed.1004683)
Supplement: Attachment — Submitted filename: Response_to_Editorial_requests.docx [file pmed.1004683.s008.docx]

Requests from Editors:

* In The Abstract:
Please use active voice (here and throughout)

please qualify the analysis as post hoc or retrospective on line 40.
please specify the trials are of antimalarials on line 43/44
please remove ‘(NMA)’ from the abstract, line 51 (abbreviation can be stated in main text but is unnecessary in the Abstract as is only mentioned once). Similarly is it necessary to abbreviate the antimalarials in the Abstract given that the abbreviations are not used again in the Abstract? Please comment.
please clarify what is meant by ‘but effective response compared to” (lines 62, 63). Was there a or no significant difference?

* In the last sentence of the Abstract Methods and Findings section, please describe the main limitation(s) of the study's methodology.

Response: The language in the abstract has been revised to use active voice. The requested additions have been made and abbreviations that were not used again in the abstract have now been removed. A clarification has been added to lines 62-63. A mention of the main limitations has been added to the abstract.

* In the author summary, in the final bullet point of 'What Do These Findings Mean?', please include the main limitations of the study in non-technical language.

Response: The main limitations of the study have been included in the final bullet point of the author summary.

* In Figure legends, when a p value is given, please specify the statistical test used to determine it.

Response: A clarification has been added to the legend of Figure 5.

* In Figures, please show graph axes beginning at zero. If this is not possible, please show a break in the axis.

Response: We have adjusted the figure axes to start at zero (Figure 2A-B and Figure 4A).

* Please lighten the colour scheme in Figure 5 as the black font is difficult to read on the dark grey shading.

Response: We have adjusted the colour scheme in Figure 5 and added a white box around the numbers in Figure 5B to increase readability.

* In the Kaplan-Meier curve(s) please provide the number at risk for each time interval. Please ensure that the data included in the curves is presented in a supplementary table or Excel spreadsheet.

Response: We have provided the number at risk for each time interval, as well as all the data included in the curves in supplementary table 14. We believe including the number at risk in the figure may compromise the figure’s readability.

* In the Introduction, please very briefly explain what a mosquito feeding assay is and measures, line 124, for clarity for the general reader.

Response: A sentence has been added to the introduction to explain what mosquito feeding assays are to the reader: “These assays involve feeding mosquitoes on blood from infected individuals and allow for measurement of mosquito infection rate, oocyst density, and the proportion of individuals who are infectious.”

* Please clarify in the methods the consent for the clinical studies so that readers don’t need to go to the primary sources—was written informed consent obtained for all study participants?

Response: A clarification about consent in the clinical studies has been added to the methods: “Before screening and before study enrolment, participants provided written informed consent (if they were aged ≥18 years) or written parental consent for participants younger than 18 years. In addition to parental consent, oral assent was sought for individuals aged 12–17 years in PQ01, PQ03, NECTAR1-2 and 10-17 years in NECTAR3-4.”

* Line 303—please define DMFA

Response: “direct membrane feeding assays” has been added to the text.

* Please ensure that all antimalarial abbreviations are spelled out in the main text (i.e. after the Abstract, even if used in the Abstract).

Response: We have checked this and confirm that all antimalarial abbreviations are spelled out in the procedures paragraph of the methods section.

* Line 355—microscopic rather than microscopical?

Response: this has been replaced with microscopy-detected.

* Line 412—abrogated rather than annulled?

Response: this has been replaced with abrogated.

* Please check for use of patient-centered language. Please note that patient-centered language is constructed with the use of post-modified nouns (e.g. 'patients with psoriasis’ (or similar) instead of ‘psoriasis patients’) putting the person first in the sentence structure. Please consider whether use of ‘carriers’ is patient-centered.

Response: ‘carrier’ has now been replaced with:

- Line 308: asymptomatic parasite carriers: individuals with an asymptomatic infection
- Line 313: gametocyte carriers: participants with detectable gametocytes
- Line 465: gametocyte carriers: individuals with *P. falciparum* gametocytaemia

* Lines 450, 488—qualify claim of primacy with ‘to our knowledge’.

Response: ‘To our knowledge’ has been added to both sentences.

* Last paragraph of Discussion—please find a synonym/rephrase one of the uses of ‘confirm’. Paragraph should focus on the novel findings of this manuscript, unless the confirmation of prior findings sheds new light/points in a new direction, in which case please discuss that aspect of the work.

Response: One use of ‘confirm’ has been replaced with ‘emphasize’.

* Please include the appropriate reporting checklist for the NMA and include it as supplementary information.

Response: A completed PRISMA checklist has been added as supplementary information (appendix 3).
